# ﻿DNA barcode library of Portuguese water mites, with the descriptions of two new species (Acari, Hydrachnidia)

**DOI:** 10.3897/zookeys.1217.131730

**Published:** 2024-10-31

**Authors:** Vladimir Pešić, Andrzej Zawal, Sónia Ferreira, Laura Benitez-Bosco, Ana Cruz-Oliveira, Dinis Girão, Adriana Padilha, Paolo Turaccio, Samantha Rossini, Lorenzo Ballini, Giorgia Staffoni, Sara Fratini, Claudio Ciofi, Alessio Iannucci, Torbjørn Ekrem, Elisabeth Stur

**Affiliations:** 1 Department of Biology, University of Montenegro, Cetinjski put b.b., 81000 Podgorica, Montenegro University of Montenegro Podgorica Montenegro; 2 Institute of Marine and Environmental Sciences, Center of Molecular Biology and Biotechnology, University of Szczecin, Wąska 13, 71–415 Szczecin, Poland University of Szczecin Szczecin Poland; 3 CIBIO, Centro de Investigação em Biodiversidade e Recursos Genéticos, InBIO Laboratório Associado, Campus de Vairão, Universidade do Porto, 4485-661 Vairão, Vila do Conde, Portugal Universidade do Porto Vila do Conde Portugal; 4 BIOPOLIS Program in Genomics, Biodiversity and Land Planning, CIBIO, Campus de Vairão, 4485-661 Vairão, Vila do Conde, Portugal BIOPOLIS Program in Genomics, Biodiversity and Land Planning Vila do Conde Portugal; 5 Departamento de Biologia, Faculdade de Ciências, Universidade do Porto, 4099-002 Porto, Portugal EBM, Estação Biológica de Mértola Mértola Portugal; 6 EBM, Estação Biológica de Mértola, Praça Luís de Camões, Mértola, Portugal EBM, Estação Biológica de Mértola, Praça Luís de Camões Mértola Portugal; 7 Department of Biology, University of Florence, Sesto Fiorentino, Italy University of Florence Sesto Fiorentino Italy; 8 Department of Natural History, NTNU University Museum, Norwegian University of Science and Technology, Trondheim, Norway Norwegian University of Science and Technology Trondheim Norway

**Keywords:** Cytochrome c oxidase subunit I (COI), DNA barcoding, Iberian Peninsula, integrative taxonomy, Portugal, systematics

## Abstract

This study presents the first results from the analysis of water mites collected in Portugal as part of the Biodiversity Genomics Europe project. 307 COI DNA barcodes clustered into 75 BINs are provided, with 38 BINs being unique and deposited for the first time in the Barcode of Life Data Systems (BOLD). 65 species have been identified, of which 36 are new to the water mite fauna of Portugal. Two species, *Torrenticolasoniae* Pešić, **sp. nov.** and *T.elisabethae* Pešić, **sp. nov.** (Torrenticolidae), are described as new to science. 47% of the water mite species currently known from Portugal now have reference barcodes in BOLD. High intraspecific distances were recorded for some species, suggesting the presence of cryptic diversity and species complexes that needs further study. Our results improve the DNA barcode reference database for Portuguese water mites, enhancing species identification accuracy and stimulating future investigation.

## ﻿Introduction

With nearly 7,500 species grouped into 550 genera (Smit 2020), water mites (Hydrachnidia) are the most diverse and abundant group of arachnids in freshwater habitats (Davids et al. 2007). They inhabit a wide range of aquatic habitats, including lotic, lentic, temporary, and interstitial waters (Davids et al. 2007). Despite the fact that water mites can be good indicators of ecosystem health, especially for groundwater dependent ecosystems, such as springs, the more significant involvement of this limnofaunistic group in rapid assessment programs is still limited by their often time-consuming taxonomic identification (Pešić et al. 2021a).

Knowledge of water mites in Portugal is still insufficient. The checklist published by Cantallo et al. (2022) listed 93 species from 34 genera and 16 families for Portugal and summarized all previous research on water mites in Portugal and its archipelagos (Madeira and Azores). Recently, Pešić et al. (2023b) added seven more species new for the water mite fauna of Portugal, one of which, *Atractidesmarizae* Pešić, 2023, was new to science.

In recent years, the use of the DNA barcode fragment of the mitochondrial cytochrome c oxidase subunit I (COI) gene has proven to be a very effective tool for the identification of water mites (e.g., Pešić et al. 2017, 2022b, 2023a; Blattner et al. 2019). A large number of regional DNA barcoding initiatives resulted in the formation of national and regional water mites DNA barcode libraries in some parts of Europe, such as, for example, Montenegro (http://dx.doi.org/10.5883/DS-MNEHYD; Pešić et al. 2021a), Corsica (http://dx.doi.org/10.5883/DS-CORHYD; Pešić and Smit 2022), Iran and Turkey (http://dx.doi.org/10.5883/DS-TRIRHYD; Pešić et al. 2023c), Norway (http://dx.doi.org/10.5883/DS-NOHYD; Gerecke et al. 2022). However, extensive sampling for DNA barcode reference library building is yet to be performed for a large part of Europe, including for countries like Portugal which have only been partially sampled (Pešić et al. 2023b).

In 2015, the Research Network in Biodiversity and Evolutionary Biology (InBIO) launched the DNA Barcoding Initiative (IBI) with the aim of forming a reference collection of DNA barcodes, focusing on Portuguese invertebrate taxa (Ferreira et al. 2020a). As a result, several datasets holding DNA barcodes of freshwater organisms have been published, namely on Megaloptera, Plecoptera and Trichoptera (Ferreira et al. 2019; Ferreira et al. 2020b; Pauperio et al. 2023). More recently the local efforts to represent countries’ biodiversity in DNA barcode reference collections have been combined with wider endeavors. The implementation of the project Biodiversity Genomics Europe (BGE, https://biodiversitygenomics.eu/) aims to address the global biodiversity crisis by providing a deeper understanding of the diversity of life on Earth through genomics. Additional goals are to develop and strengthen functioning communities of practice, and to build capacity and complementarity across activities and borders. The barcode stream of the project focuses on increasing the geographic and taxonomic representation of understudied organisms in publicly accessible databases. By contributing to the DNA barcode reference library of invertebrate taxa, the project promotes improved assessment and monitoring of macroinvertebrates, including water mites. Moreover, the DNA barcode data provides valuable input for integrative taxonomic research of challenging species groups, especially of cryptic or pseudocryptic species that likely would remain undiscovered using only morphological features.

This is the first in a series of papers related to the diversity of Portuguese water mites that will present the results and ongoing public releases of the DNA barcodes in BOLD. The paper is based on specimens collected in continental Portugal in 2023 and COI records that are publicly available in the Barcode of Life Data Systems (BOLD). As a result of this investigation, we describe two new species to science, and report 36 species of water mites from Portugal for the first time.

## ﻿Material and methods

Water mites were collected with kick nets and immediately preserved in 96% ethanol for the purpose of molecular analyses. Water mite specimens used for the molecular study are listed in Table 1. After non-destructive, whole-body DNA extraction, the specimen vouchers were stored in 96% ethanol and morphologically examined. Some of these vouchers were dissected and slide mounted in Faure’s medium, while the rest was transferred to Koenike’s fluid.

**Table 1.** Details of DNA barcoded specimens, including localities and coordinates of sampling sites, sample codes, and the barcode index number codes (^N^ indicates a new BIN that contains only sequences from this study). BOLD data presented here was last accessed on 10 May 2024.

Taxa	Locality	Coordinates	Sample ID	Process ID	BIN
**Eylaidae**					
*Eylaistantilla*	Beja, São João dos Caldeireiros (stream)	37.625°N, 7.810°W	BGE_00110_B03	BSNTN490-23	^N^ BOLD:AFP3591
			BGE_00110_B04	BSNTN491-23	
	Beja, Herdade de Alagães (stream)	37.676°N, 7.853°W	BGE_00110_D01	BSNTN512-23	
			BGE_00110_D02	BSNTN513-23	
			BGE_00110_D03	BSNTN514-23	
	Beja, Herdade de Alagães (pond)	37.673°N, 7.848°W	BGE_00110_D08	BSNTN519-23	
	Beja, Moinho de Alferes 1	37.502°N, 7.690°W	BGE_00228_C09	BSNTN888-23	
	Beja, São Miguel do Pinheiro	37.552°N, 7.850°W	BGE_00228_G01	BSNTN928-23	
	Beja, Herdade de Alagães	37.676°N, 7.853°W	BGE_00228_H01	BSNTN940-23	
**Limnocharidae**					
*Limnocharesaquatica*	Guarda, Poço do Inferno	40.373°N, 7.516°W	BGE_00227_B10	BSNTN972-23	^N^ BOLD:AFV0270
			BGE_00108_B03	BBIOP110-24	
			BGE_00108_B04	BBIOP111-24	
	Guarda, Casa do Loureiro	40.433°N, 7.701°W	BGE_00109_D05	BBIOP041-24	
**Hydryphantidae**					
*Hydrodromadespiciens*	Guarda, Covão do Forno	40.369°N, 7.638°W	BGE_00227_D11	BSNTN997-23	BOLD:ACS0426
			BGE_00108_A02	BBIOP097-24	
*Protziaannularis*	Faro, Parque do Barranco dos Pisões	37.333°N, 8.567°W	BGE_00228_E09	BSNTN912-23	^N^ BOLD:AFX2700
**Lebertiidae**					
*Lebertiafimbriata*	Beja, São João dos Caldeireiros	37.626°N, 7.810°W	BGE_00110_A02	BSNTN477-23	BOLD:AEI5359
			BGE_00110_B02	BSNTN489-23	
*Lebertiasparsicapillata*	Beja, Zambujeira do Mar	37.399°N, 8.723°W	BGE_00110_F01	BSNTN536-23	BOLD:AFN4501
	Guarda, Casais de Folgosinho	40.454°N, 7.493°W	BGE_00227_F12	BSNTN1022-23	
	Guarda, Praia fluvial de Vila Cova a Coelheira	40.379°N, 7.736°W	BGE_00228_A10	BSNTN865-23	
	Guarda, Casa do Loureiro	40.433°N, 7.701°W	BGE_00109_H05	BBIOP089-24	
*Lebertiavariolata*	Beja, Zambujeira do Mar	37.398°N, 8.68°W	BGE_00228_D06	BSNTN897-23	BOLD:ADK0996
	Faro, Portimão	37.237°N, 8.546°W	BGE_00228_F08	BSNTN923-23	
*Lebertiapilosa*	Beja, Zambujeira do Mar	37.399°N, 8.723°W	BGE_00110_E11	BSNTN534-23	BOLD:AEJ2601
			BGE_00110_E12	BSNTN535-23	
			BGE_00110_F07	BSNTN542-23	
			BGE_00228_F01	BSNTN916-23	
*Lebertiagibbosa*	Guarda, Praia Fluvial de Sabugueiro	40.401°N, 7.640°W	BGE_00227_A08	BSNTN958-23	BOLD:ACR9744
			BGE_00227_B03	BSNTN965-23	
	Guarda, Ponte dos Frades	40.403°N, 7.526°W	BGE_00108_D12	BBIOP143-24	
	Guarda, Central hidroelétrica de Ponte dos Jugai	40.385°N, 7.706°W	BGE_00108_F01	BBIOP156-24	
	Guarda, Nossa Senhora do Desterro	40.395°N, 7.694°W	BGE_00108_F06	BBIOP161-24	
	Guarda, Covão da ponte	40.443°N, 7.514°W	BGE_00108_G06	BBIOP173-24	
	Guarda, Casais de Folgosinho	40.454°N, 7.493°W	BGE_00108_G11	BBIOP178-24	
	Porto, Moinho da Tapada	41.263°N, 8.307°W	BGE_00109_F05	BBIOP065-24	
*Lebertiaalgeriensis*	Guarda, Casais de Folgosinho	40.454°N, 7.493°W	BGE_00227_F07	BSNTN1017-23	^N^ BOLD:AFV0271
	Guardia, Praia fluvial de Vila Cova a Coelheira	40.379°N, 7.736°W	BGE_00228_A03	BSNTN858-23	
			BGE_00108_E09	BBIOP152-24	
*Lebertiainsignis*	Guarda, Poço do Inferno	40.373°N, 7.516°W	BGE_00227_D05	BSNTN991-23	BOLD:AEB9107
	Porto, Moinho da Tapada	41.263°N, 8.307°W	BGE_00109_E11	BBIOP059-24	
	Guarda, Praia fluvial de Vila Cova a Coelheira	40.379°N, 7.736°W	BGE_00228_A04	BSNTN859-23	^N^ BOLD:AFW6960
*Lebertiaporosa* aggr. sp. A	Guarda, Praia Fluvial de Sabugueiro	40.401°N, 7.640°W	BGE_00227_A10	BSNTN960-23	BOLD:ACP5319
			BGE_00109_A02	BBIOP002-24	
	Guarda, Poço do Inferno	40.373°N, 7.516°W	BGE_00227_D01	BSNTN987-23	
			BGE_00108_B08	BBIOP115-24	
	Guarda, Nossa Senhora do Desterro	40.395°N, 7.694°W	BGE_00228_C02	BSNTN881-23	
	Guarda, Praia fluvial de Vila Cova a Coelheira	40.379°N, 7.736°W	BGE_00108_E06	BBIOP149-24	
			BGE_00108_E08	BBIOP151-24	
	Guarda, Covão da ponte	40.443°N, 7.514°W	BGE_00108_F11	BBIOP166-24	
			BGE_00108_G03	BBIOP170-24	
	Guarda, Casais de Folgosinho	40.454°N, 7.493°W	BGE_00108_G10	BBIOP177-24	
*Lebertiaporosa* aggr. sp. ACS0974	Guarda, Praia Fluvial de Sabugueiro	40.401°N, 7.640°W	BGE_00227_B07	BSNTN969-23	BOLD:ACS0974
	Guarda, Casais de Folgosinho	40.454°N, 7.493°W	BGE_00227_E12	BSNTN1010-23	
			BGE_00227_F09	BSNTN1019-23	
	Guarda, Poio do Leão	40.399°N, 7.541°W	BGE_00227_H10	BSNTN1044-23	
	Guarda, Ponte dos Frades	40.403°N, 7.526°W	BGE_00108_D10	BBIOP141-24	
	Guarda, Praia fluvial de Vila Cova a Coelheira	40.379°N, 7.736°W	BGE_00108_E05	BBIOP148-24	
	Guarda, Nossa Senhora do Desterro	40.395°N, 7.694°W	BGE_00108_F08	BBIOP163-24	
	Bragança, Torre de Dona Chama	41.665°N, 7.146°W	BGE_00109_C01	BBIOP025-24	
	Bragança, Gasparona	41.85°N, 7.013°W	BGE_00109_C12	BBIOP036-24	
	Porto, Moinho da Tapada	41.263°N, 8.307°W	BGE_00109_F01	BBIOP061-24	
*Lebertiapusilla*	Porto, Moinho da Tapada	41.263°N, 8.307°W	BGE_00109_E09	BBIOP057-24	BOLD:AFW6961
**Oxidae**					
*Oxusangustipositus*	Guarda, Poço do Inferno	40.373°N, 7.516°W	BGE_00227_B12	BSNTN974-23	BOLD:AET9442
	Guarda, Casa do Cantoneiro	40.418°N, 7.603°W	BGE_00227_E02	BSNTN1000-23	
			BGE_00108_H04	BBIOP183-24	
*Oxuslusitanicus*	Guarda, Casais de Folgosinho	40.454°N, 7.493°W	BGE_00227_E09	BSNTN1007-23	^N^ BOLD:AFX3224
			BGE_00227_E11	BSNTN1009-23	
	Beja, Moinho de Alferes 2	37.503°N, 7.687°W	BGE_00228_E05	BSNTN908-23	
			BGE_00228_E06	BSNTN909-23	
	Bragança, Torre de Dona Chama	41.665°N, 7.146°W	BGE_00109_A07	BBIOP007-24	
			BGE_00109_A08	BBIOP008-24	
	Vila Real, Noura stream	41.409°N, 7.417°W	BGE_00109_D11	BBIOP047-24	
*Oxusmusculus*	Beja, Moinho de Alferes 1	37.502°N, 7.690°W	BGE_00228_C10	BSNTN889-23	BOLD:AFC2154
*Oxusovalis*	Beja, Zambujeira do Mar	37.399°N, 8.723°W	BGE_00110_F08	BSNTN543-23	^N^ BOLD:AFP5747
			BGE_00228_F02	BSNTN917-23	
*Oxussetosus*	Guarda, Casais de Folgosinho	40.454°N, 7.493°W	BGE_00227_E10	BSNTN1008-23	BOLD:ACS0808
	Guarda, Covão da ponte	40.443°N, 7.514°W	BGE_00228_C03	BSNTN882-23	
**Sperchontidae**					
*Sperchonalgeriensis*	Faro, Portimão	37.237°N, 8.546°W	BGE_00228_F12	BSNTN927-23	BOLD:AES2436
*Sperchonclupeifer*	Guarda, Casais de Folgosinho	40.454°N, 7.493°W	BGE_00227_F04	BSNTN1014-23	^N^ BOLD:AFX0389
*Sperchoncompactilis*	Faro, Portimão	37.237°N, 8.546°W	BGE_00110_E03	BSNTN526-23	BOLD:AER7687
	Guarda, Praia fluvial de Vila Cova a Coelheira	40.379°N, 7.736°W	BGE_00228_A07	BSNTN862-23	
**Torrenticolidae**					
*Monatractidesmadritensis*	Guarda, Praia Fluvial de Sabugueiro	40.401°N, 7.640°W	BGE_00227_A12	BSNTN962-23	BOLD:AED3803
	Guarda, Poço do Inferno	40.373°N, 7.516°W	BGE_00227_C09	BSNTN983-23	
	Guarda, Poio do Leão	40.399°N, 7.541°W	BGE_00227_H07	BSNTN1041-23	
			BGE_00108_D02	BBIOP133-24	
	Bragança, Gasparona	41.850°N, 7.013°W	BGE_00109_C06	BBIOP030-24	
*Monatractidesstadleri*	Faro, Parque do Barranco dos Pisões	37.333°N, 8.567°W	BGE_00110_G03	BSNTN550-23	BOLD:AEU1504
	Guarda, Casais de Folgosinho	40.454°N, 7.493°W	BGE_00227_F01	BSNTN1011-23	
	Guarda, Praia fluvial de Vila Cova a Coelheira	40.379°N, 7.736°W	BGE_00228_A06	BSNTN861-23	
			BGE_00108_E01	BBIOP144-24	
	Guarda, Central hidroelétrica de Ponte dos Jugais	40.385°N, 7.706°W	BGE_00228_B01	BSNTN868-23	
	Porto, Moinho da Tapada	41.263°N, 8.307°W	BGE_00109_E07	BBIOP055-24	
	Guarda, Casa do Loureiro	40.433°N, 7.701°W	BGE_00109_G11	BBIOP083-24	
*Monatractidesstenostomus*	Beja, Corte do Pinto	37.682°N, 7.512°W	BGE_00228_G08	BSNTN935-23	^N^ BOLD:AFU3082
*Torrenticolaelliptica*	Bragança, Gasparona	41.850°N, 7.013°W	BGE_00109_C09	BBIOP033-24	BOLD:AEI9183
*Torrenticolatenuipalpis*	Guarda, Casais de Folgosinho	40.454°N, 7.493°W	BGE_00227_F03	BSNTN1013-23	^N^ BOLD:AFV2021
*Torrenticolasoniae* sp. nov.	Guarda, Praia Fluvial de Sabugueiro	40.401°N, 7.640°W	BGE_00227_A11	BSNTN961-23	^N^ BOLD:AFW5337
	Guarda, Casais de Folgosinho	40.454°N, 7.493°W	BGE_00227_F02	BSNTN1012-23	
	Guarda, Ponte dos Frades	40.403°N, 7.526°W	BGE_00108_D09	BBIOP140-24	
	Guarda, Praia fluvial de Vila Cova a Coelheira	40.379°N, 7.736°W	BGE_00108_E02	BBIOP145-24	
			BGE_00108_E07	BBIOP150-24	
	Bragança, Torre de Dona Chama	41.665°N, 7.146°W	BGE_00109_C03	BBIOP027-24	
*Torrenticolaelisabethae* sp. nov.	Guarda, Poço do Inferno	40.373°N, 7.516°W	BGE_00227_C10	BSNTN984-23	^N^ BOLD:AFW5336
	Guarda, Poio do Leão	40.399°N, 7.541°W	BGE_00227_H06	BSNTN1040-23	
			BGE_00108_D04	BBIOP135-24	
**Limnesiidae**					
*Limnesiaacuminata*	Beja, Moinho de Alferes 1	37.502°N, 7.690°W	BGE_00228_C12	BSNTN891-23	^N^ BOLD:AFU7587
	Bragança, Torre de Dona Chama	41.665°N, 7.146°W	BGE_00109_B01	BBIOP013-24	
			BGE_00109_B03	BBIOP015-24	
			BGE_00109_B04	BBIOP016-24	
*Limnesiaiberica*	Beja, São João dos Caldeireiros	37.626°N, 7.810°W	BGE_00110_A03	BSNTN478-23	^N^ BOLD:AFN8367
			BGE_00110_A04	BSNTN479-23	
			BGE_00110_A05	BSNTN480-23	
			BGE_00110_A06	BSNTN481-23	
			BGE_00110_A11	BSNTN486-23	
			BGE_00110_A12	BSNTN487-23	
			BGE_00110_H08	BSNTN567-23	
			BGE_00110_H09	BSNTN568-23	
			BGE_00110_H11	BSNTN570-23	
*Limnesiakoenikei*	Guarda, Covão do Forno	40.369°N, 7.638°W	BGE_00227_D09	BSNTN995-23	BOLD:ADF6559
			BGE_00108_A01	BBIOP096-24	
			BGE_00108_A03	BBIOP098-24	
	Guarda, Central hidroelétrica de Ponte dos Jugais	40.385°N, 7.706°W	BGE_00228_B02	BSNTN869-23	
*Limnesiamaculata*	Guarda, Praia Fluvial de Sabugueiro	40.401°N, 7.640°W	BGE_00227_B05	BSNTN967-23	^N^ BOLD:AFW6935
	Guarda, Barragem do Lagoacho	40.385°N, 7.618°W	BGE_00108_C07	BBIOP126-24	
	Beja, Moinho de Alferes 2	37.503°N, 7.687°W	BGE_00228_E03	BSNTN906-23	
	Beja, Herdade de Alagães	37.673°N, 7.848°W	BGE_00228_H04	BSNTN943-23	
			BGE_00228_H06	BSNTN945-23	
*Limnesiawalteri*	Beja, Corte do Pinto	37.682°N, 7.512°W	BGE_00110_B11	BSNTN498-23	^N^ BOLD:AFO9873
			BGE_00110_C02	BSNTN501-23	
			BGE_00228_G09	BSNTN936-23	
	Bragança, Torre de Dona Chama	41.665°N, 7.146°W	BGE_00109_B09	BBIOP021-24	
**Hygrobatidae**					
*Atractidesinflatus*	Beja, Zambujeira do Mar	37.398°N, 8.680°W	BGE_00110_E05	BSNTN528-23	BOLD:AFI9009
			BGE_00228_D07	BSNTN898-23	BOLD:ACB4677
	Vila Real, Noura stream	41.409°N, 7.417°W	BGE_00109_D07	BBIOP043-24	
*Atractidesmarizae*	Guarda, Casais de Folgosinho	40.454°N, 7.493°W	BGE_00227_F11	BSNTN1021-23	BOLD:AER7878
	Guarda, Praia fluvial de Vila Cova a Coelheira	40.379°N, 7.736°W	BGE_00228_A08	BSNTN863-23	
	Faro, Caldas de Monchique	37.287°N, 8.554°W	BGE_00228_D12	BSNTN903-23	
	Odeceixe, Covão da Serva	37.374°N, 8.642°W	BGE_00228_E11	BSNTN914-23	
	Faro, Portimão	37.237°N, 8.546°W	BGE_00228_F07	BSNTN922-23	
*Atractidesnodipalpis*	Guarda, Praia fluvial de Vila Cova a Coelheira	40.379°N, 7.736°W	BGE_00228_A01	BSNTN856-23	^N^ BOLD:AFV2009
*Atractidesrobustus*	Guarda, Covão da Ametade	40.328°N, 7.587°W	BGE_00227_G07	BSNTN1029-23	BOLD:AFF2463
*Hygrobatesbalcanicus*	Faro, Portimão	37.237°N, 8.546°W	BGE_00110_E01	BSNTN524-23	BOLD:AEG3198
			BGE_00110_E02	BSNTN525-23	
			BGE_00228_F06	BSNTN921-23	
	Porto, Moinho da Tapada	41.263°N, 8.307°W	BGE_00109_D12	BBIOP048-24	
			BGE_00109_E10	BBIOP058-24	
	Porto, Parque Molinológico e Florestal de Pias	41.268°N, 8.256°W	BGE_00109_F09	BBIOP069-24	
	Porto, Rio Este	41.378°N, 8.695°W	BGE_00109_G07	BBIOP079-24	
			BGE_00109_G08	BBIOP080-24	
			BGE_00109_G09	BBIOP081-24	
			BGE_00109_H11	BBIOP095-24	
*Hygrobatesfluviatilis*	Guarda, Casa do Cantoneiro	40.418°N, 7.603°W	BGE_00227_E03	BSNTN1001-23	BOLD:ACB4846
	Guarda, Casais de Folgosinho	40.454°N, 7.493°W	BGE_00227_F10	BSNTN1020-23	
	Guarda, Praia fluvial de Vila Cova a Coelheira	40.379°N, 7.736°W	BGE_00228_A09	BSNTN864-23	
			BGE_00108_E03	BBIOP146-24	
	Guarda, Praia Fluvial de Sabugueiro	40.401°N, 7.640°W	BGE_00109_A04	BBIOP004-24	
*Hygrobateslongiporus* complex	Guarda, Praia fluvial de Vila Cova a Coelheira	40.379°N, 7.736°W	BGE_00228_A02	BSNTN857-23	^N^ BOLD:AFV9997
		40.379°N, 7.736°W	BGE_00108_E04	BBIOP147-24	
	Guarda, Ponte dos Frades	40.403°N, 7.526 °W	BGE_00108_D11	BBIOP142-24	
	Gurad, Covão da ponte	40.443°N, 7.514°W	BGE_00108_G04	BBIOP171-24	
	Guarda, Praia Fluvial de Sabugueiro	40.401°N, 7.640°W	BGE_00109_A03	BBIOP003-24	
	Guarda, Casa do Loureiro	40.433°N, 7.701°W	BGE_00109_H07	BBIOP091-24	
			BGE_00109_H08	BBIOP092-24	
	Bragança, Torre de Dona Chama	41.665°N, 7.146°W	BGE_00109_B05	BBIOP017-24	^N^ BOLD:AFW1423
	Vila Real, Noura stream	41.409°N, 7.417°W	BGE_00109_D09	BBIOP045-24	
	Guarda, Praia Fluvial de Sabugueiro	40.401°N, 7.640°W	BGE_00227_A09	BSNTN959-23	^N^ BOLD:AFV9998
			BGE_00227_B01	BSNTN963-23	
			BGE_00227_B02	BSNTN964-23	
			BGE_00109_A01	BBIOP001-24	
	Guarda, Poço do Inferno	40.373°N, 7.516°W	BGE_00227_D03	BSNTN989-23	
			BGE_00227_D04	BSNTN990-23	
			BGE_00108_B07	BBIOP114-24	
	Guarda, Casais de Folgosinho	40.454°N, 7.493°W	BGE_00227_F06	BSNTN1016-23	
			BGE_00227_F08	BSNTN1018-23	
	Guarda, Poio do Leão	40.399°N, 7.541°W	BGE_00108_D03	BBIOP134-24	
	Gurada, Central hidroelétrica de Ponte dos Jugais	40.385°N, 7.706°W	BGE_00108_F02	BBIOP157-24	
	Guarda, Nossa Senhora do Desterro	40.395°N, 7.694°W	BGE_00108_F10	BBIOP165-24	
	Guarda, Covão da ponte	40.443°N, 7.514°W	BGE_00108_F12	BBIOP167-24	
			BGE_00108_G05	BBIOP172-24	
			BGE_00108_G07	BBIOP174-24	
			BGE_00108_G08	BBIOP175-24	
			BGE_00108_G09	BBIOP176-24	
**Unionicolidae**					
*Neumaniaelliptica*	Guarda, Poço do Inferno	40.373°N, 7.516°W	BGE_00227_D02	BSNTN988-23	^N^ BOLD:AFU2122
	Guarda, Nossa Senhora do Desterro	40.395°N, 7.694°W	BGE_00228_B12	BSNTN879-23	
*Neumaniaimitata*	Porto, Parque Molinológico e Florestal de Pias	41.268°N, 8.256°W	BGE_00109_G02	BBIOP074-24	^N^ BOLD:AFV0268
*Neumanialimosa*	Beja, Herdade de Alagães	37.673°N, 7.848°W	BGE_00110_D06	BSNTN517-23	BOLD:ACS0551
			BGE_00110_D07	BSNTN518-23	
			BGE_00110_D09	BSNTN520-23	
			BGE_00228_H05	BSNTN944-23	
	Guarda, Lagoa	40.350°N, 7.549°W	BGE_00227_G01	BSNTN1023-23	
			BGE_00227_G02	BSNTN1024-23	
			BGE_00108_B01	BBIOP108-24	
			BGE_00108_B02	BBIOP109-24	
	Guarda, Barragem do Lagoacho	40.385°N, 7.618°W	BGE_00227_H04	BSNTN1038-23	
*Neumaniauncinata*	Faro, Caldas de Monchique	37.287°N, 8.554°W	BGE_00228_E01	BSNTN904-23	^N^ BOLD:AFV0253
	Porto, Rio Este	41.378°N, 8.695°W	BGE_00109_G04	BBIOP076-24	^N^ BOLD:AFV0269
*Neumaniapapillosa*	Beja, Corte do Pinto	37.682°N, 7.512°W	BGE_00110_C01	BSNTN500-23	^N^ BOLD:AFO2116
			BGE_00228_G10	BSNTN937-23	
			BGE_00228_G11	BSNTN938-23	
	Guarda, Praia Fluvial de Sabugueiro	40.401°N, 7.640°W	BGE_00227_A05	BSNTN955-23	
*Unionicolaminor*	Beja, São João dos Caldeireiros	37.626°N, 7.810°W	BGE_00110_H06	BSNTN565-23	^N^ BOLD:AFO2171
	Beja, Moinho de Alferes 2	37.503°N, 7.687°W	BGE_00228_E02	BSNTN905-23	
**Pionidae**					
*Forelialongipalpis*	Guarda, Nossa Senhora do Desterro	40.395°N, 7.694°W	BGE_00228_C01	BSNTN880-23	^N^ BOLD:AFV3893
	Guarda, Covão da ponte	40.443°N, 7.514°W	BGE_00228_C05	BSNTN884-23	
	Guarda, Barragem do Vale do Rossim	40.4°N, 7.589°W	BGE_00108_C02	BBIOP121-24	
			BGE_00108_C03	BBIOP122-24	
			BGE_00227_H02	BSNTN1036-23	
			BGE_00108_C04	BBIOP123-24	
*Foreliavariegator*	Guarda, Central hidroelétrica de Ponte dos Jugais	40.385°N, 7.706°W	BGE_00228_B06	BSNTN873-23	^N^ BOLD:AFU5459
	Beja, São João dos Caldeireiros	37.626°N, 7.81°W	BGE_00228_H09	BSNTN948-23	
	Porto, Parque Molinológico e Florestal de Pias	41.268°N, 8.256°W	BGE_00109_F12	BBIOP072-24	
			BGE_00109_G01	BBIOP073-24	
	Porto, Rio Este	41.378°N, 8.695°W	BGE_00109_G05	BBIOP077-24	
	Guarda, Nossa Senhora do Desterro	40.395°N, 7.694°W	BGE_00108_F05	BBIOP160-24	
*Hydrochoreuteskrameri*	Beja, Herdade de Alagães (pond)	37.673°N, 7.848°W	BGE_00110_D11	BSNTN522-23	BOLD:ACR9737
	Guarda, Covão do Forno	40.369°N, 7.638°W	BGE_00227_D12	BSNTN998-23	
*Tiphystorris*	Beja, Zambujeira do Mar	37.399°N, 8.723°W	BGE_00110_F02	BSNTN537-23	BOLD:ACR9977
	Guarda, Praia Fluvial de Sabugueiro	40.401°N, 7.64°W	BGE_00227_A06	BSNTN956-23	
	Guarda, Nossa Senhora do Desterro	40.395°N, 7.694°W	BGE_00228_B10	BSNTN877-23	
*Nautarachnacrassa*	Guarda, Casa do Cantoneiro	40.418°N, 7.603°W	BGE_00227_E01	BSNTN999-23	^N^ BOLD:AFV0462
*Pionacarnea*	Guarda, Lagoa	40.350°N, 7.549°W	BGE_00108_A10	BBIOP105-24	BOLD:ACS0622
	Beja, São Sebastião dos Carros	37.598°N, 7.754°W	BGE_00110_G05	BSNTN552-23	BOLD:ACM0527
			BGE_00110_G06	BSNTN553-23	
			BGE_00110_G07	BSNTN554-23	
			BGE_00110_G08	BSNTN555-23	
			BGE_00110_G12	BSNTN559-23	
			BGE_00228_D08	BSNTN899-23	
			BGE_00228_D10	BSNTN901-23	
	Beja, Herdade de Alagães	37.678°N, 7.848°W	BGE_00228_E08	BSNTN911-23	
	Vila Real, Noura stream	41.409°N, 7.417°W	BGE_00109_D10	BBIOP046-24	
	Guarda, Lagoa	40.350°N, 7.549°W	BGE_00108_A10	BBIOP105-24	BOLD:ACS0622
			BGE_00108_A12	BBIOP107-24	
*Pionavariabilis*	Beja, São Sebastião dos Carros	37.598°N, 7.754°W	BGE_00110_H01	BSNTN560-23	BOLD:AAU0701
*Pionopsislutescens*	Porto, Parque Torre de Vilar	41.287°N, 8.210°W	BGE_00109_F06	BBIOP066-24	^N^ BOLD:AFV3897
	Porto, Parque Molinológico e Florestal de Pias	41.268°N, 8.256°W	BGE_00109_F11	BBIOP071-24	
	Guarda, Cise	40.419°N, 7.709°W	BGE_00109_A05	BBIOP005-24	
			BGE_00109_A06	BBIOP006-24	
			BGE_00227_B08	BSNTN970-23	
**Aturidae**					
*Aturusscaber*	Porto, Moinho da Tapada	41.263°N, 8.307°W	BGE_00109_E03	BBIOP051-24	BOLD:ACQ9097
	Porto, Parque Molinológico e Florestal de Pias	41.268°N, 8.256°W	BGE_00109_G03	BBIOP075-24	
**Mideopsidae**					
*Mideopsisroztoczensis*	Beja, Moinho de Alferes 1	37.502°N, 7.690°W	BGE_00110_B10	BSNTN497-23	^N^ BOLD:AFP5421
	Beja, Pulo do Lobo	37.805°N, 7.633°W	BGE_00110_C08	BSNTN507-23	
			BGE_00110_C09	BSNTN508-23	
			BGE_00110_C10	BSNTN509-23	
			BGE_00110_C11	BSNTN510-23	
		37.805°N, 7.633°W	BGE_00110_C12	BSNTN511-23	
			BGE_00228_G12	BSNTN939-23	
	Vila Real, Noura stream	41.409°N, 7.417°W	BGE_00109_D08	BBIOP044-24	
	Beja, Zambujeira do Mar	37.398°N, 8.680°W	BGE_00228_D03	BSNTN894-23	
	Bragança, Gasparona	41.850°N, 7.013°W	BGE_00109_C07	BBIOP031-24	^N^ BOLD:AFU6108
			BGE_00109_C10	BBIOP034-24	^N^ BOLD:AFW3785
	Guarda, Ponte dos Frades	40.403°N, 7.526°W	BGE_00108_D06	BBIOP137-24	
	Guarda, Casa do Loureiro	40.433°N, 7.701°W	BGE_00109_D03	BBIOP039-24	^N^ BOLD:AFU6108
			BGE_00109_D04	BBIOP040-24	
			BGE_00109_G10	BBIOP082-24	
			BGE_00109_G12	BBIOP084-24	^N^ BOLD:AFW3785
			BGE_00109_H02	BBIOP086-24	^N^ BOLD:AFU6108
	Guarda, Covão da Ametade	40.328°N, 7.587°W	BGE_00108_A06	BBIOP101-24	
			BGE_00227_G05	BSNTN1027-23	^N^ BOLD:AFU6108
			BGE_00227_G09	BSNTN1031-23	
	Guarda, Poço do Inferno	40.373°N, 7.516°W	BGE_00108_B09	BBIOP116-24	
			BGE_00227_C04	BSNTN978-23	^N^ BOLD:AEV2909
			BGE_00227_C06	BSNTN980-23	
			BGE_00227_C08	BSNTN982-23	^N^ BOLD:AFU6108
	Guarda, Casais de Folgosinho	40.454°N, 7.493°W	BGE_00108_G12	BBIOP179-24	
			BGE_00227_F05	BSNTN1015-23	
	Guarda, Poio do Leão	40.399°N, 7.541°W	BGE_00108_C12	BBIOP131-24	
			BGE_00227_H08	BSNTN1042-23	
	Guarda, Central hidroelétrica de Ponte dos Jugais	40.385°N, 7.706°W	BGE_00228_B03	BSNTN870-23	
			BGE_00228_B08	BSNTN875-23	
			BGE_00228_B09	BSNTN876-23	
			BGE_00108_E10	BBIOP153-24	
			BGE_00108_E11	BBIOP154-24	
	Guarda, Nossa Senhora do Desterro	40.395°N, 7.694°W	BGE_00108_F04	BBIOP159-24	
			BGE_00108_F09	BBIOP164-24	
	Guarda, Covão da ponte	40.443°N, 7.514°W	BGE_00108_G01	BBIOP168-24	
	Guarda, Casa do Cantoneiro	40.418°N, 7.603°W	BGE_00108_H02	BBIOP181-24	
			BGE_00227_E04	BSNTN1002-23	^N^ BOLD:AFV6334
			BGE_00227_E05	BSNTN1003-23	^N^ BOLD:AFU6108
	Guarda, Praia Fluvial de Sabugueiro	40.401°N, 7.640°W	BGE_00108_H09	BBIOP188-24	
			BGE_00227_A07	BSNTN957-23	
**Momoniidae**					
*Momoniafalcipalpis*	Guarda, Poço do Inferno	40.373°N, 7.516°W	BGE_00108_B10	BBIOP117-24	^N^ BOLD:AFX3396
**Arrenuridae**					
*Arrenurusalbator*	Guarda, Barragem do Vale do Rossim	40.400°N, 7.589°W	BGE_00227_G11	BSNTN1033-23	BOLD:ACR9639
			BGE_00227_G12	BSNTN1034-23	
			BGE_00227_H01	BSNTN1035-23	
			BGE_00108_B11	BBIOP118-24	
	Guarda, Barragem do Lagoacho	40.400°N, 7.589°W	BGE_00108_C08	BBIOP127-24	
*Arrenurusszalayi*	Beja, Moinho de Alferes	37.502°N, 7.690°W	BGE_00110_B05	BSNTN492-23	BOLD:ACS0403
			BGE_00110_B06	BSNTN493-23	
			BGE_00228_C06	BSNTN885-23	
			BGE_00228_C07	BSNTN886-23	
			BGE_00228_C08	BSNTN887-23	
*Arrenurusleuckarti*	Guarda, Poço do Inferno	40.373°N, 7.516°W	BGE_00227_B11	BSNTN973-23	BOLD:ACR9670
	Guarda, Casa do Cantoneiro	40.418°N, 7.603°W	BGE_00108_H03	BBIOP182-24	
*Arrenurusneumani*	Beja, Moinho de Alferes 1	37.502°N, 7.690°W	BGE_00110_B07	BSNTN494-23	^N^ BOLD:AFP6143
			BGE_00110_B08	BSNTN495-23	
			BGE_00228_C11	BSNTN890-23	
			BGE_00228_H10	BSNTN949-23	
*Arrenurustricuspidator*	Beja, Moinho de Alferes 1	37.502°N, 7.690°W	BGE_00228_D01	BSNTN892-23	^N^ BOLD:AFU3639
*Arrenurusglobator*	Beja, Moinho de Alferes 1	37.502°N, 7.690°W	BGE_00110_B09	BSNTN496-23	^N^ BOLD:AFO3503
	Beja, São Sebastião dos Carros	37.598°N, 7.754°W	BGE_00110_H02	BSNTN561-23	
			BGE_00110_H03	BSNTN562-23	
			BGE_00110_H04	BSNTN563-23	
	Beja, São Miguel do Pinheiro	37.552°N, 7.850°W	BGE_00228_G02	BSNTN929-23	
			BGE_00228_G03	BSNTN930-23	
			BGE_00228_G04	BSNTN931-23	
*Arrenuruszachariae*	Bragança, Gasparona	41.850°N, 7.013°W	BGE_00109_C08	BBIOP032-24	^N^ BOLD:AFU0319

Morphological nomenclature follows Gerecke et al. (2016). The distribution data are from Cantallo et al. (2022) unless stated otherwise. The dorsal platelets of *Torrenticola* spp. were measured on both sides, therefore their dimensions were given as a range of values, rather than a single number. The holotype and paratypes of the new species are deposited in the Naturalis Biodiversity Center in Leiden (**RMNH**).

All measurements are given in μm. The photographs of selected structures were made using a camera on Samsung Galaxy smartphone. The following abbreviations are used: **Ac-1** = first acetabulum; **Cx-I** = first coxae; **Cxgl-4** = coxoglandularia 4; **dL** = dorsal length; **H** = height; **I-L-4–6** = fourth-sixth segments of first leg; **L** = length; **mL** = medial length; **P-1**–**P-5** = palp segments 1–5; **Vgl-1** = ventroglandularia 1; **W** = width.

## ﻿Molecular and DNA barcode analyses

The molecular analysis was conducted at the University of Florence (Florence, Italy). DNA was extracted using a non-destructive protocol. Samples were digested using 95 μl of extraction buffer (100 mM Tris-HCl, 5 mM EDTA, 100 mM NaCl, 0.5% SDS, pH 8) and 5 μl of proteinase K. Dilutions (1:10) of crude digested samples were used as template for the amplification of the mitochondrial cytochrome c oxidase subunit I (COI). Amplicons were amplified and barcoded in a single-step PCR using a cocktail of two barcoded primer pairs, namely Folmer primers (LCO1490, HC02198; Folmer et al. 1994) and Lep primers (LepF1, LepR1; Hebert et al. 2004). PCR was performed using the Kapa3G Plant PCR Kit according to the manufacturer’s protocol and with the following thermal profile: initial denaturation step of 3 min at 94 °C, 35 cycles of 20 s at 95 °C, annealing for 15 s at 52 °C and extension for 30 s at 72 °C, and a final extension for 1 min at 72 °C. Amplicons were checked on a 1.2% agarose gel and pooled in a single tube. The amplicon mix was used to prepare a PacBio library with the SMRTbell prep kit 3.0 according to the manufacturer’s protocol. The library was sequenced on a 8M ZMW SMRT cell on a PacBio Sequel IIe platform.

Raw reads were demultiplexed using the Pacific Biosciences SMRT Link software. Consensus sequences were generated with the PacBio Amplicon Analysis (pbaa) tool. Primer trimming, translation and stop codon checking were performed using Geneious Prime 2024.0.1.

Consensus sequences were made available in the Barcode of Life Data Systems (BOLD) (Ratnasingham and Hebert 2007), and the Barcode Index Numbers (BIN) were obtained, grouping DNA sequences based on the Refined Single Linkage (RESL) analysis performed in BOLD (Ratnasingham and Hebert 2013). BINs are often considered proxies for species (e.g., Hebert et al. 2016). Relevant voucher information, photos, and newly generated DNA barcodes are publicly accessible through the Dataset - DS-BGEPL01 BGE Biodiversity Genomics Europe: Portuguese water mites I https://www.boldsystems.org/index.php/MAS_Management_DataConsole?codes=DS-BGEPL01) in BOLD. Data related to each BIN, including the minimum *p*-distance to the nearest neighboring BIN, was estimated using BOLD tools. The dataset consists of 307 sequences generated through this study (Suppl. material 1).

Sequence alignments were performed using MUSCLE (Edgar 2004). Intra- and interspecific genetic distances were calculated based on the Kimura 2-parameter model (K2P; Kimura 1980), using MEGA X (Kumar et al. 2018). The latter software was used to calculate Neighbor-Joining (NJ) trees based on K2P distances (standard for barcoding studies) using pairwise deletion for missing data. Branch support was calculated using nonparametric bootstrap (Felsenstein 1985) with 1000 replicates and shown next to the branches. All codon positions were considered in the analyses.

## ﻿Results and discussion

We generated 307 DNA barcodes from 65 water mite species. The collected water mites represent 15 families of the 16 recorded in Portugal. The most sequence-rich family was Hygrobatidae with 51 sequences (16.7% of total; 10 BINs), followed by Lebertiidae wih 47 sequences (15.4%; 11 BINs), Mideopsidae with 41 sequences (13.4%; 5 BINs), Pionidae with 35 sequences (11.5%; 10 BINs), Limnesiidae with 26 sequences (8.5%; 5 BINs), Arrenuridae with 25 sequences (8.2%; 5 BINs), and Torrenticolidae with 24 sequences (7.9%; 7 BINs). Some families were rare, such as Hydryphantidae and Momoniidae, represented by a single sequence each and corresponding single BIN.

Our findings added the first records of 34 species for Portugal: *Eylaistantilla* Koenike, 1897 (Eylaidae), *Lebertiasparsicapillata* Thor, 1905, *L.variolata* Gerecke, 2009, *L.gibbosa* Lundblad, 1926, *L.algeriensis* Lundblad, 1942, *L.insignis* Neuman, 1880, *L.porosa* aggr. sp. A (Lebertiidae), *Oxusmusculus* (Müller, 1776), *O.ovalis* (Müller, 1776), *O.setosus* (Koenike, 1898) (Oxidae), *Sperchonalgeriensis* Lundblad, 1942, *S.compactilis* Koenike, 1911 (Sperchontidae), *Monatractidesmadritensis* (K. Viets, 1930), *Torrenticolaelliptica* Maglio, 1909 (Torrenticolidae), *Limnesiakoenikei* Piersig, 1894 (Limnesiidae), *Atractidesinflatus* (Walter, 1925), *A.robustus* (Sokolow, 1940), *Hygrobatesbalcanicus* Pešić, 2020 (Hygrobatidae), *Neumaniaelliptica* Walter, 1925, *N.imitata* Koenike, 1908, *N.limosa* (Koch, 1836), *Unionicolaminor* (Soar, 1900) (Unionicolidae), *Forelialongipalpis* Maglio, 1924, *Hydrochoreuteskrameri* Piersig, 1896, *Nautarachnacrassa* (Koenike, 1908), *Pionacarnea* (Müller, 1776), *P.variabilis* (Koch, 1836) (Pionidae), *Aturusscaber* Kramer, 1875 (Aturidae), *Mideopsisroztoczensis* Biesiadka & Kowalik, 1979 (Mideopsidae), *Momoniafalcipalpis* Halbert, 1906 (Momoniidae), *Arrenurusleuckarti* Piersig, 1894, *A.neumani* Piersig, 1895, A.cf.tricuspidator (Müller, 1776) and A.cf.zachariae Koenike, 1886 (Arrenuridae). Two species of the genus *Torrenticola* are described as new to science. Even though sampling was focused on certain districts (i.e., Beja, Bragança, Faro, Guarda, Porto, Vila Real), we recorded specimens from 47.4% of the Portuguese water mite fauna (65 of 137 species including 36 species new to Portugal).

The resulting sequences clustered into 75 BINs, with 38 BINs (51%) being unique and deposited for the first time in BOLD. The number of BINs per species ranged from one (58 species, 89%) to five for *Mideopsisroztoczensis* (BOLD:AFU6108, BOLD:AFP5421, BOLD:AFW3785, BOLD:AFV6334, BOLD:AEV2909). Two BINs were detected for five species, *Lebertiainsignis* (BOLD:AEB9107, BOLD:AFW6960), *Atractidesinflatus* (BOLD:AFI9009, BOLD:ACB4677), *Neumaniauncinata* (BOLD:AFV0253, BOLD:AFV0269), *Forelialongipalpis* (BOLD:AFX2876, BOLD:AFV3893), *Pionacarnea* (BOLD:ACM0527, BOLD:ACS0622), and one species, *Hygrobateslongiporus* (BOLD:AFV9997, BOLD:AFW1423, BOLD:AFV9998), has three BINs.

Our study provided the first DNA barcodes for *Protziaannularis* Lundblad, 1954 (BOLD:AFX2700), *Monatractidesstenostomus* (K. Viets, 1930) (BOLD:AFU3082), *Torrenticolatenuipalpis* (Lundblad, 1956) (BOLD:AFV2021), *Oxuslusitanicus* Lundblad, 1954 (BOLD:AFX3224), *Limnesiaacuminata* Walter, 1925 (BOLD:AFU7587), *L.iberica* Lundblad, 1954 (BOLD:AFN8367), *L.walteri* Migot, 1926 (BOLD:AFO9873), *Neumaniaelliptica* (BOLD:AFU2122), *N.papillosa* (Soar, 1902) (BOLD:AFO2116), *Momoniafalcipalpis* (BOLD:AFX3396) and *Arrenurusszalayi* Lundblad, 1954 (BOLD:ACS0403).

### ﻿Systematics

#### ﻿Family Eylaidae Leach, 1815

##### ﻿Genus Eylais

Taxon classificationAnimaliaHydrachnidiaEylaidae

Latreille, 1796

81AEBB3D-CC1F-558F-AE0A-9C8AEAF4B7EB

###### Note.

Only one species reported from Portugal.

##### ﻿ Eylais tantilla

Taxon classificationAnimaliaHydrachnidiaEylaidae

Koenike, 1897

B5402981-8F6C-5C4F-907B-D112C0CF3E39

###### Material examined.

Portugal, **Beja**: • Mértola, São João dos Caldeireiros, stream, 37.625°N, 7.81°W, 17 May 2023, leg. Ferreira, Benitez-Bosco, Ekrem, Stur & Turaccio, 1♂ (sequenced), dissected and slide mounted (RMNH); • Mértola, Moinho de Alferes 1, 37.502°N, 7.69°W, 19 May 2023, leg. Ferreira, Benitez-Bosco, Ekrem, Stur & Turaccio, 1♀ (sequenced); • Mértola, Herdade de Alagães, dry stream site 1, 37.676°N, 7.853°W, 18 May 2023, leg. Ferreira, Benitez-Bosco, Ekrem, Stur & Turaccio 1♀ (sequenced).

###### Remarks.

As a result of the treatment during the barcoding process, all vouchered individuals except one male and two females were partly or completely destroyed. With regards to the shape of the eye bridge and gnathosoma, the specimens examined in our study matches the description of *E.tantilla* given by K. Viets (1930) for material from Spain. The sequenced specimens from Portugal form a unique BIN (BOLD:AFP3591), with the nearest neighboring BIN being BOLD:ACS1138, which includes three public sequences of specimens from Norway assigned to *E.rimosa* Piersig, 1899, and three unpublished sequences of specimens from the Netherlands, two of them assigned to *E.extendens* and one assigned to *E.setosa* Koenike, 1897. The *p*-distance between these two BINs was estimated at 14.83%.

###### Distribution.

Palaearctic. New for Portugal.

#### ﻿Family Limnocharidae Grube, 1859

##### ﻿Genus Limnochares

Taxon classificationAnimaliaHydrachnidiaLimnocharidae

Latreille, 1796

C17355CF-08A5-5A15-A1CA-970776D74A9F

###### Note.

Only one species reported from Portugal.

##### ﻿ Limnochares aquatica

Taxon classificationAnimaliaHydrachnidiaLimnocharidae

(Linnaeus, 1788)

047C4043-F917-5DAC-8374-C9F384CF6303

###### Material examined.

Portugal, **Guarda**: • Manteigas, Poço do Inferno, 40.373°N, 7.516°W, 1078 m a.s.l., 21 Aug. 2023, leg. Ferreira, Benitez-Bosco & Padilha, 1♀ (sequenced); • Seia, Casa do Loureiro, 40.433°N, 7.701°W, 415 m a.s.l., 19 Jul. 2023 leg. Ferreira & Padilha, 1♀ (sequenced).

###### Remarks.

The examined specimens in our study, keyed to *L.aquatica* following Davids et al. (2007), form a unique BIN (BOLD:AFV0270). The *p*-distance between the latter BIN and its nearest neighbor, BOLD:ACS0438, which includes specimens of *L.aquatica* from the Netherlands, Norway, Montenegro, and Italy, was estimated at 11.72%, indicating the need for taxonomic revision of *L.aquatica* complex to identify possible undescribed cryptic species.

###### Distribution.

Holarctic. In Portugal previously reported from Beira Alta and Alentejo (Lundblad 1956).

#### ﻿Family Hydrodromidae Viets, 1936

##### ﻿Genus Hydrodroma

Taxon classificationAnimaliaHydrachnidiaHydrodromidae

Koch, 1837

90231F63-46BD-56ED-B1F1-1AA6390B2FDE

###### Note.

Only one species reported from Portugal.

##### ﻿ Hydrodroma despiciens

Taxon classificationAnimaliaHydrachnidiaHydrodromidae

(Müller, 1776)

227F79ED-7EC9-5AF5-9DC6-97C5B4EAA692

###### Material examined.

Portugal, **Guarda**: • Seia, Covão do Forno, 40.369°N, 7.638°W, 1574 m a.s.l., 19 Aug. 2023, leg. Ferreira, Benitez-Bosco & Padilha, 1♂, 1 deutonymph (sequenced).

###### Remarks.

The sequences obtained from the specimens from Portugal fall into BOLD:ACS0426, which, in addition to the specimens used in this study for molecular analysis, includes 17 specimens of *H.despiciens* from the Netherlands, Norway, and Poland, available in the BOLD database.

###### Distribution.

Europe. In Portugal previously reported from Alentejo (Lundblad 1956).

#### ﻿Family Hydryphantidae Piersig, 1896

##### ﻿Genus Protzia

Taxon classificationAnimaliaHydrachnidiaHydryphantidae

Piersig, 1896

9AF7AB15-3662-5FE7-8998-48C8901A4F0A

###### Note.

Only three species reported from Portugal.

##### ﻿ Protzia annularis

Taxon classificationAnimaliaHydrachnidiaHydryphantidae

Lundblad, 1954

AF29D7D0-ECFB-5D48-ABC9-100778E44EEF

###### Material examined.

Portugal, **Faro**: • Monchique, Ribeira de Seixe, Parque do Barranco dos Pisões, 37.333°N, 8.567°W, 480 m a.s.l., 23 May 2023, leg. Ekrem & Benitez-Bosco, 1♀ (sequenced).

###### Remarks.

The single examined female from Ribeira de Seixe matches the description of *P.annularis*, a species known from Portugal and Spain (Lundblad 1956). The Portuguese specimen forms a unique BIN (BOLD:AFX2700), with the nearest neighboring BIN being BOLD:AEI5748, which includes specimens of *P.lata* from Corsica. The *p*-distance between these two BINs was estimated at 12.36%.

###### Distribution.

Iberian Peninsula. In Portugal previously reported from Beira Alta (Lundblad 1956).

#### ﻿Family Lebertiidae Thor, 1900

##### ﻿Genus Lebertia

Taxon classificationAnimaliaHydrachnidiaLebertiidae

Neuman, 1880

E40A51FC-954F-5F95-A03F-5F33725458DC

###### Note.

Nine species known from Portugal, two of them, *Lebertiamadericola* (Lundblad, 1942) and *Lebertiamaderigena* (Lundblad, 1942), are endemic for Madeira.

##### ﻿ Lebertia (Lebertia) fimbriata

Taxon classificationAnimaliaHydrachnidiaLebertiidae

Thor, 1899

F58907BE-66B8-5F2C-8C9B-CC564120CF63

###### Material examined.

Portugal, **Beja**: • Mértola, São João dos Caldeireiros, stream, 37.626°N, 7.81°W, 17 May 2023, leg. Ferreira, Benitez-Bosco, Ekrem, Stur & Turaccio, 1♀, 1♂ (sequenced).

###### Remarks.

The specimens from Portugal match the description of *Lebertiafimbriata*, a species widely distributed in the Western Palaearctic (Di Sabatino et al. 2010). The Portuguese specimens were clustered within BOLD:AEI5359, which includes specimens of *L.fimbriata* from Germany, Spain and North Macedonia.

###### Distribution.

Western Palaearctic. In Portugal previously reported from Fonte Fria in Mealhada (Buçaco mountain; Lundblad 1956).

##### ﻿ Lebertia (Lebertia) sparsicapillata

Taxon classificationAnimaliaHydrachnidiaLebertiidae

Thor, 1905

AD28B421-67B1-518E-8B79-C5E911DB435B

###### Material examined.

Portugal, **Beja**: • Odemira, Ribeira de Seixe, Zambujeira do Mar, river, 37.399°N, 8.723°W, 45 m a.s.l., 23 May 2023, leg. Ekrem & Benitez-Bosco, 1♂ (sequenced). **Guarda**: • Gouveia, Rio Mondego, Casais de Folgosinho, 40.454°N, 7.493°W, 976 m a.s.l., 24 Aug. 2023, leg. Ferreira, Benitez-Bosco & Padilha, 1♂ (sequenced); • Seia, Rio Alva, Praia fluvial de Vila Cova a Coelheira, river, 40.379°N, 7.736°W, 312 m a.s.l., 23 Aug. 2023, leg. Ferreira, Benitez-Bosco & Padilha, 1♀ (sequenced); • Seia, Casa do Loureiro, 40.433°N, 7.701°W, 415 m a.s.l., 19 Jul. 2023 leg. Ferreira & Padilha, 1♀ (sequenced).

###### Remarks.

The specimens from Portugal match the description of *L.sparsicapillata*, a species widely distributed in Europe except for the most northern and eastern parts (Di Sabatino et al. 2010). The sequenced specimens cluster within BOLD:AFN4501, which includes two specimens from Germany. The *p*-distance between the latter BIN and its nearest neighboring BOLD:ADF6063, which include specimens of *L.sparsicapillata* from Germany, was estimated at 2%.

###### Distribution.

Europe. New for Portugal.

##### ﻿ Lebertia (Lebertia) variolata

Taxon classificationAnimaliaHydrachnidiaLebertiidae

Gerecke, 2009

E134BC45-EACE-52F1-809F-463022D8019C

###### Material examined.

Portugal, **Beja**: • Odemira, Ribeira de Seixe, Zambujeira do Mar, 37.398°N, 8.68°W, site 32, 75 m a.s.l., 23 May 2023, leg. Ekrem & Benitez-Bosco, 1♀ (sequenced); • Faro, Portimão, 37.237°N, 8.546°W, 23 May 2023, leg. Ferreira & Turaccio 1♀ (sequenced).

###### Remarks.

The sequences obtained from two specimens from Portugal fall into the *Lebertiavariolata* cluster (BOLD:ADK0996). In addition to specimens used in this study, the BIN includes specimens from Montenegro, North Macedonia and Turkey morphologically assigned to *L.variolata*, and one private sequence of a non-identified specimen from France (GBMIN118138-1). *Lebertiavariolata* is a characteristic inhabitant of streams that regularly dry up in the summer (Gerecke 2009).

###### Distribution.

Mediterranean region. New for Portugal.

##### ﻿ Lebertia (Pilolebertia) gibbosa

Taxon classificationAnimaliaHydrachnidiaLebertiidae

Lundblad, 1926

D233F93E-6CDD-5BCB-A570-B274ABF3926E

###### Material examined.

Portugal, **Guarda**: • Seia, Rio Alva, Praia Fluvial de Sabugueiro, river, 40.401°N, 7.64°W, 1021 m a.s.l., 24 Aug. 2023, leg. Ferreira, Benitez-Bosco & Padilha, 2♂ (sequenced); • Manteigas, Zêzere, Ponte dos Frades, 40.403°N, 7.526°W, 672 m a.s.l., 22 Aug. 2023, leg. Ferreira, Benitez-Bosco, Padilha, Andrade & Stur 1♂ (sequenced); • Seia, Rio Alva, Central hidroelétrica de Ponte dos Jugais, river, 40.385°N, 7.706°W, 555 m a.s.l., 23 Aug. 2023, leg. Ferreira, Benitez-Bosco & Padilha, 1♀ (sequenced); • Rio Alva, Nossa Senhora do Desterro, river, 40.395°N, 7.694°W, 791 m a.s.l., 23 Aug. 2023, leg. Ferreira, Benitez-Bosco & Padilha, 1♂ (sequenced); • Gouveia, Rio Mondego Casais de Folgosinho, 40.454°N, 7.493°W, 976 m a.s.l., 24 Aug. 2023, leg. Ferreira, Benitez-Bosco & Padilha, 1♀ (sequenced); • Manteigas, Mondego, Covão da ponte, 40.443°N, 7.514°W, 999 m a.s.l., 24 Aug. 2023, leg. Ferreira, Benitez-Bosco & Padilha, 1 deutonymph (sequenced). **Porto**, • Lousada, Moinho da Tapada, 41.263°N, 8.307°W, 178 m a.s.l., 1 Sep. 2023, Ferreira, Sousa, Cruz-Oliveira & Girão, 1 deutonymph (sequenced).

###### Remarks.

This species was originally described from the island of Gotland, Sweden (Lundblad 1956), but later on synonymized with *L.porosa*. Recently Tyukosova et al. (2022) used material from Norway to redescribe *L.gibbosa* based on morphological and molecular evidence and showed that this species is widely distributed in southern Norway.

The sequenced specimens from Portugal were clustered within BOLD:ACR9744, which, in addition to the specimens from Portugal, includes specimens of *L.gibbosa* from the Netherlands, Norway, Poland, and Germany, available in BOLD.

###### Distribution.

As this species has been widely overlooked with other species of *L.porosa* complex, the full geographical distribution of *L.gibbosa* cannot be defined without additional research.

##### ﻿ Lebertia (Pilolebertia) algeriensis

Taxon classificationAnimaliaHydrachnidiaLebertiidae

Lundblad, 1942

5A96CDD1-A847-59AA-9B13-B83AA53699D1

###### Material examined.

Portugal, **Guarda**: • Gouveia, Rio Mondego Casais de Folgosinho, 40.454°N, 7.493°W, 976 m a.s.l., 24 Aug. 2023, leg. Ferreira, Benitez-Bosco & Padilha, 1♀ (sequenced; Table 1); • Seia, Rio Alva, Praia fluvial de Vila Cova a Coelheira, river, 40.379°N, 7.736°W, 312 m a.s.l., 23 Aug. 2023, leg. Ferreira, Benitez-Bosco & Padilha, 1♂, 1♀ (sequenced).

###### Remarks.

The Portuguese specimens molecularly analyzed in this study match the description of *Lebertiaalgeriensis*. Genetic data indicate that all examined specimens form a unique BIN (BOLD:AFV0271), and the closest neighboring BIN is that of *L.inaequalis* (BOLD:AEF5683) from North Macedonia. The *p*-distance between these two BINs was estimated at 4.53%.

###### Distribution.

Palaearctic. Gerecke (2009) mentioned that several published records of *L.inaequalis* (Koch, 1837) from the Mediterranean region could refer to similar *L.longiseta* or *L.algeriensis*. New for Portugal.

##### ﻿ Lebertia (Pilolebertia) insignis

Taxon classificationAnimaliaHydrachnidiaLebertiidae

Neuman, 1880

3D811FDC-E757-5CBA-83E6-839B9DE383BA

###### Material examined.

Portugal, **Guarda**: • Manteigas, Poço do Inferno, 40.373°N, 7.516°W, 1078 m a.s.l., 21 Aug. 2023, leg. Ferreira, Benitez-Bosco & Padilha, 1♂ (sequenced); • Seia, Rio Alva, Praia fluvial de Vila Cova a Coelheira, river, 40.379°N, 7.736°W, 312 m a.s.l., 23 Aug. 2023, leg. Ferreira, Benitez-Bosco & Padilha, 1♂ (sequenced). **Porto**, • Lousada, Moinho da Tapada, 41.263°N, 8.307°W, 178 m a.s.l., 1 Sep. 2023, Ferreira, Sousa, Cruz-Oliveira & Girão, 1♀ (sequenced).

###### Remarks.

The sequenced specimens from Portugal clustered within two BINs, BOLD:AEB9107, which includes specimens of *L.insignis* from Norway, Montenegro, Poland and Slovakia, and the unique BOLD:AFW6960, which includes one specimen from this study collected in Rio Alva in Guarda Province. The *p*-distance between these two BINs was estimated at 9.79%, indicating the need for taxonomic revision of *L insignis* complex to identify possible undescribed cryptic species.

###### Distribution.

Central, Western and Northern Europe. Rare in the Mediterranean and on the Iberian Peninsula previously known only from Oviedo in Spain (Lundblad 1956). New for Portugal.

##### ﻿ Lebertia (Pilolebertia) porosa

Taxon classificationAnimaliaHydrachnidiaLebertiidae

aggr. sp. A

9778E366-6F01-5875-92C6-1A8D03C4BBA2

###### Material examined.

Portugal, **Guarda**: • Seia, Rio Alva, Praia Fluvial de Sabugueiro, river, 40.401°N, 7.64°W, 1021 m a.s.l., 24 Aug. 2023, leg. Ferreira, Benitez-Bosco & Padilha, 2♀ (sequenced); • Manteigas, Poço do Inferno, 40.373°N, 7.516°W, 1078 m a.s.l., 21 Aug. 2023, leg. Ferreira, Benitez-Bosco & Padilha, 2♀ (sequenced); • Rio Alva, Nossa Senhora do Desterro, river, 40.395°N, 7.694°W, 791 m a.s.l., 23 Aug. 2023, leg. Ferreira, Benitez-Bosco & Padilha, 1♂ (sequenced); • Seia, Rio Alva, Praia fluvial de Vila Cova a Coelheira, river, 40.379°N, 7.736°W, 312 m a.s.l., 23 Aug. 2023, leg. Ferreira, Benitez-Bosco & Padilha, 2♀ (sequenced); • Manteigas, Mondego, Covão da ponte, 40.443°N, 7.514°W, 999 m a.s.l., 24 Aug. 2023, leg. Ferreira, Benitez-Bosco & Padilha, 2♀ (sequenced); • Gouveia, Rio Mondego, Casais de Folgosinho, 40.454°N, 7.493°W, 976 m a.s.l., 24 Aug. 2023, leg. Ferreira, Benitez-Bosco & Padilha, 1♀ (sequenced).

###### Remarks.

The sequenced specimens from Portugal clustered within BOLD:ACP5319, which includes *porosa*-like specimens from Serbia and Spain, and specimens from Germany and Norway provisionally assigned by Tyukosova et al. (2022) to *L.porosa* aggr. sp. A.

###### Distribution.

As this species has been widely overlooked with other species of *L.porosa* complex, the full geographical distribution of *L.porosa* aggr. sp. A. can be defined only on the basis of the records available in BOLD.

##### ﻿ Lebertia (Pilolebertia) porosa

Taxon classificationAnimaliaHydrachnidiaLebertiidae

aggr. sp.

856B45BC-B41B-584D-A102-BDE95944DE9A

###### Material examined.

Portugal, **Guarda**: • Seia, Rio Alva, Praia Fluvial de Sabugueiro, river, 40.401°N, 7.64°W, 1021 m a.s.l., 24 Aug. 2023, leg. Ferreira, Benitez-Bosco & Padilha, 1♀ (sequenced); • Gouveia, Rio Mondego, Casais de Folgosinho, 40.454°N, 7.493°W, 976 m a.s.l., 24 Aug. 2023, leg. Ferreira, Benitez-Bosco & Padilha, 1♂, 1♀ (sequenced); • Manteigas, Poio do Leão, 40.399°N, 7.541°W, 734 m a.s.l., 22 Aug. 2023, leg. Ferreira, Benitez-Bosco & Padilha, 1♀ (sequenced); • Manteigas, Zêzere, Ponte dos Frades, 40.403°N, 7.526°W, 672 m a.s.l., 22 Aug. 2023, leg. Ferreira, Benitez-Bosco, Padilha, Andrade & Stur, 1♀ (sequenced); • Seia, Rio Alva, Praia fluvial de Vila Cova a Coelheira, river, 40.379°N, 7.736°W, 312 m a.s.l., 23 Aug. 2023, leg. Ferreira, Benitez-Bosco & Padilha, 1♀ (sequenced); • Seia, Rio Alva, Nossa Senhora do Desterro, river, 40.395°N, 7.694°W, 791 m a.s.l., 23 Aug. 2023, leg. Ferreira, Benitez-Bosco & Padilha, 1 deutonymph (sequenced). **Bragança**: • Mirandela, Torre de Dona Chama, 41.665°N, 7.146°W, 256 m a.s.l., 13 Jul. 2023, leg. Ferreira & Padilha, 1 deutonymph (sequenced); • Vinhais, Gasparona, 41.85°N, 7.013°W, 693 m a.s.l., 6 Jul. 2023, leg. Ferreira & Padilha, 1 deutonymph (sequenced). **Porto** • Lousada, Moinho da Tapada, 41.263°N, 8.307°W, 178 m a.s.l., 1 Sep. 2023, Ferreira, Sousa, Cruz-Oliveira & Girão, 1♀ (sequenced).

###### Remarks.

The sequenced specimens from Portugal clustered within BOLD:ACS0974, which includes *porosa*-like specimens from different parts of Europe, except Fennoscandia, available in BOLD. In the phylogenetic tree, the BIN is positioned as a sister clade of *L.porosa* as defined by Tyukosova et al. (2022). The latter species was recently redefined by Tyukosova et al. (2022) based on the specimens from the type locality that were shown to belong to BOLD:ACQ9049. The taxonomic status of *L.porosa* like species of BOLD:ACS0974 needs to be clarified by resolving taxonomic status of numerous species listed as synonyms in Gerecke (2009). As emphasized by Gerecke et al. (2022), a more extensive study of *L.porosa* complex is needed to establish a stable taxonomy for this group.

###### Distribution.

As this species has been widely overlooked with other species of *L.porosa* complex, the geographical distribution of *L.porosa* like species of BOLD:ACS0974 can be defined only on the basis of the records available in BOLD.

##### ﻿ Lebertia (Pilolebertia) pilosa

Taxon classificationAnimaliaHydrachnidiaLebertiidae

Maglio, 1924

B4E5898B-7B28-537B-91C8-5CC94EFA070C

###### Material examined.

Portugal, **Beja**: • Odemira, Ribeira de Seixe, Zambujeira do Mar, river, 37.399°N, 8.723°W, 45 m a.s.l., 23 May 2023, leg. Ekrem & Benitez-Bosco, 4♀ (sequenced), 1♀ dissected and slide mounted (RMNH).

###### Remarks.

The sequenced specimens from Portugal were clustered within BOLD:AEJ2601, which, in addition to material from this study, include one unidentified *Lebertia* specimen from Spain. The *p*-distance between the latter BIN and its nearest neighbor, BOLD:ACS0974, which include *L.porosa* like specimens from different parts of Europe, was estimated at 12.01%.

###### Distribution.

Europe. In Portugal previously reported from Minho River (Cantallo et al. 2021), and from São Pedro da Torre in Valença municipality (Cantallo et al. 2022).

##### ﻿ Lebertia (Lebertia) pusilla

Taxon classificationAnimaliaHydrachnidiaLebertiidae

Koenike, 1911

C6EF2CBA-79B8-542D-AB21-CF7D77A2B07D

###### Material examined.

Portugal, **Porto**: • Lousada, Moinho da Tapada, 41.263°N, 8.307°W, 178 m a.s.l., 1 Sep. 2023, Ferreira, Sousa, Cruz-Oliveira & Girão, 1 ♂ (sequenced).

###### Remarks.

The sequenced specimen from Moinho da Tapada cluster together with specimens collected by Pešić et al. (2023b) from Santarém, Portugal, and morphologically assigned to *L.pusilla*. These specimens form a unique BIN (BOLD:AFW6961), with a *p*-distance of 10.43% to the nearest sequence (NLACA493-15) of *L.pusilla* from the Netherlands.

###### Distribution.

Europe.

#### ﻿Family Oxidae K. Viets, 1926

##### ﻿Genus Oxus

Taxon classificationAnimaliaHydrachnidiaOxidae

Kramer, 1877

6733003B-5B79-57E7-A0D2-825E014AA9F0

###### Note.

Four species known for Portugal: two of them, *Oxushastata* (Lundblad, 1954) and *O.lusitanicus* Lundblad, 1954, originally described from Portugal. *Oxusoblongus* Kramer, 1879, reported by Lundblad (1956) from Sintra, is a possible synonym of *O.strigatus* (Di Sabatino et al. 2010; Smit and Gerecke 2010).

##### ﻿ Oxus (Oxus) cf.angustipositus

Taxon classificationAnimaliaHydrachnidiaOxidae

K. Viets, 1908

09B3B135-ED7B-5F2F-9136-69371DF28061

###### Material examined.

Portugal, **Guarda**: • Manteigas, Casa do Cantoneiro, 40.418°N, 7.603°W, 1378 m a.s.l., 24 Aug. 2023, leg. Ferreira, Benitez-Bosco & Padilha, 2♂ (sequenced); • Manteigas, Poço do Inferno, 40.373°N, 7.516°W, 1078 m a.s.l., 21 Aug. 2023, leg. Ferreira, Benitez-Bosco & Padilha, 1♀ (sequenced).

###### Remarks.

The examined specimens cluster within BOLD:AET9442, which includes specimens from Portugal assigned by Pešić et al. (2023b) to Oxuscf.angustipositus. As emphasized by Pešić et al. (2023b) taxonomic revision of the *O.angustipositus* complex is required for identifying possibly undescribed cryptic species.

###### Distribution.

Europe. In Portugal previously reported from Porto (Pešić et al. 2023b).

##### ﻿ Oxus (Oxus) lusitanicus

Taxon classificationAnimaliaHydrachnidiaOxidae

Lundblad, 1954

028247DE-9BB5-5A78-B425-A678B817FD8E

###### Material examined.

Portugal, **Guarda**: • Gouveia, Rio Mondego, Casais de Folgosinho, 40.454°N, 7.493°W, 976 m a.s.l., 24 Aug. 2023, leg. Ferreira, Benitez-Bosco & Padilha, 1♀, 1 deutonymph (sequenced). **Beja** • Mértola, Moinho de Alferes 2, 37.503°N, 7.687°W, 19 May 2023, leg. Ferreira, Benitez-Bosco, Ekrem, Stur & Turaccio, 1♂, 1♀ (sequenced). **Bragança** • Mirandela, Torre de Dona Chama, 41.665°N, 7.146°W, 256 m a.s.l., 13 Jul. 2023, leg. Ferreira & Padilha, 1♂, 1♀ (sequenced). **Vila Real** • Murça, Noura stream, 41.409°N, 7.417°W, 421 m a.s.l., 12 Jul. 2023 leg. Ferreira & Padilha, 1♀ (sequenced).

###### Remarks.

The examined specimens match the description of *Oxuslusitanicus*, a species originally described by Lundblad (1954) based on a single male collected in Côa River in Portugal. The specimens from Portugal used in this study for molecular analysis form a unique BIN (BOLD:AFX3224) with the nearest neighboring BIN being BOLD:ACL5934, which includes specimens of unidentified *Oxus* sp. from Canada, with the *p*-distance estimated at 14.34%.

###### Distribution.

Portugal previously recorded from Beira Alta (Lundblad 1956).

##### ﻿ Oxus (Oxus) musculus

Taxon classificationAnimaliaHydrachnidiaOxidae

(Müller, 1776)

3DAC928B-874B-50EF-8E9E-5A4583AFB0A5

###### Material examined.

Portugal, **Beja** • Mértola, Moinho de Alferes 1, 37.502°N, 7.69°W, 19 May 2023, leg. Ferreira, Benitez-Bosco, Ekrem, Stur & Turaccio, 1♀ (sequenced).

###### Remarks.

The sequenced specimens from Portugal were clustered within BOLD:AFC2154, which includes specimens of *O.musculus* from Norway.

###### Distribution.

Palaearctic. Widespread in Europe, but here reported for the first time for Portugal.

##### ﻿ Oxus (Oxus) ovalis

Taxon classificationAnimaliaHydrachnidiaOxidae

(Müller, 1776)

225BB002-8575-507C-AC19-B8015140147B

###### Material examined.

Portugal, **Beja** • Odemira, Ribeira de Seixe, Zambujeira do Mar, 37.399°N, 8.723°W, 45 m a.s.l., 23 May 2023, leg. Ekrem & Benitez-Bosco, 1♂, 1♀ (sequenced).

###### Remarks.

The sequenced specimens from Zambujeira do Mar, keyed to *Oxusovalis* following Di Sabatino et al. (2010), form a unique BIN (BOLD:AFP5747).

###### Distribution.

Widespread in Europe, but here reported for the first time for Portugal.

##### ﻿ Oxus (Gnaphiscus) setosus

Taxon classificationAnimaliaHydrachnidiaOxidae

(Koenike, 1898)

0129B0C6-B522-59F2-84F9-73731795966A

###### Material examined.

Portugal, **Guarda**: • Gouveia, Rio Mondego, Casais de Folgosinho, 40.454°N, 7.493°W, 976 m a.s.l., 24 Aug. 2023, leg. Ferreira, Benitez-Bosco & Padilha, 1♀ (sequenced); • Manteigas, Mondego, Covão da ponte, 40.443°N, 7.514°W, 999 m a.s.l., 24 Aug. 2023, leg. Ferreira, Benitez-Bosco & Padilha, 1♀ (sequenced).

###### Remarks.

The sequenced specimens from Portugal clustered within BOLD:ACS0808, which includes specimens of *O.setosus* from the Netherlands.

###### Distribution.

Palaearctic. Widespread in Europe, but here reported for the first time for Portugal.

#### ﻿Family Sperchontidae Thor, 1900

##### ﻿Genus Sperchon

Taxon classificationAnimaliaHydrachnidiaSperchontidae

Kramer, 1877

CFCA4159-25F1-522C-BBCE-D5C3BF34DFD4

###### Note.

Five species known from Portugal.

##### ﻿ Sperchon (Hispidosperchon) algeriensis

Taxon classificationAnimaliaHydrachnidiaSperchontidae

Lundblad, 1942

F39CFB6C-5E57-546C-A116-6BBE121B7D0C

###### Material examined.

Portugal, **Faro** • Portimão, 37.237°N, 8.546°W, 23 May 2023, leg. Ferreira & Turaccio 1♀ (sequenced).

###### Remarks.

The single female from Portugal clusters within BOLD:AES2436, which includes one specimen of *S.algeriensis* recently collected from eastern Spain (López-Peña et al. 2022). The species was described from northern Africa (Lundblad 1942) and subsequently recorded from many sites in the central and western Mediterranean area (Di Sabatino et al. 2010). The species is considered as a characteristic species of warm Mediterranean streams that regularly dry up in the summer (Gerecke 1991). The hydrography of the sampling site where *S.algeriensis* was found in our study is characterized by summer drought.

The high genetic distance of 15.4% between Iberian populations of *S.algeriensis* and a specimen from Iran, attributed to *S.algeriensis*, suggests that the latter belongs to a further distinct species (Pešić et al. 2022a). It is likely that the latter species represents *S.beneckei* Bader & Sepasgosarian, 1982, a species proposed to be a synonym of *S.algeriensis* by Asadi et al. (2010). Therefore, the known populations of *S.algeriensis* from Eastern Mediterranean should be checked using molecular methods to see if they can be assigned to *S.beneckei*.

###### Distribution.

North Africa, west Mediterranean. New record for Portugal.

##### ﻿ Sperchon (Hispidosperchon) clupeifer

Taxon classificationAnimaliaHydrachnidiaSperchontidae

Piersig, 1896

39AE44BF-5138-52CA-B7E2-0A5A5F56F907

###### Material examined.

Portugal, **Guarda** • Gouveia, Rio Mondego, Casais de Folgosinho, 40.454°N, 7.493°W, 976 m a.s.l., 24 Aug. 2023, leg. Ferreira, Benitez-Bosco & Padilha, 1♂ (sequenced).

###### Remarks.

The single male from Portugal used in this study for molecular analysis matches the description of *S.clupeifer*. This specimen forms a unique BIN (BOLD:AFX0389). The *p*-distance to its nearest neighboring BIN (BOLD:ACS1100), which includes specimens of *S.clupeifer* from the Netherlands, Germany, Norway, Macedonia, Montenegro, Russia, Serbia, Austria, and Italy, was estimated at 7.06%.

###### Distribution.

Western Palaearctic. In Portugal previously reported from Fonte Fria in Mealhada (Buçaco Mountain; Lundblad 1956).

##### ﻿ Sperchon (Hispidosperchon) compactilis

Taxon classificationAnimaliaHydrachnidiaSperchontidae

Koenike, 1911

B6108B6F-7829-5ADD-9B27-0CC0821CEB16

###### Material examined.

Portugal, **Beja** • Faro, Portimão, 37.237°N, 8.546°W, 23 May 2023, leg. Ferreira & Turaccio 1♀ (sequenced). **Guarda** • Seia, Rio Alva, Praia fluvial de Vila Cova a Coelheira, river, 40.379°N, 7.736°W, 312 m a.s.l., 23 Aug. 2023, leg. Ferreira, Benitez-Bosco & Padilha, 1♀ (sequenced).

###### Remarks.

The sequences obtained from two Portuguese specimens, keyed to *S.compactilis* following Di Sabatino et al. (2010), clustered in BOLD:AER7687, which, in addition to specimens used in this study, includes two specimens from Austria and Spain.

###### Distribution.

Western Palaearctic. New record for Portugal.

#### ﻿Family Torrenticolidae Piersig, 1902

##### ﻿Genus Monatractides

Taxon classificationAnimaliaHydrachnidiaTorrenticolidae

K. Viets, 1926

86F8A74D-2CE7-5018-B283-6B33D933252F

###### Note.

So far, two species of the genus are known from Portugal.

##### ﻿ Monatractides (Monatractides) madritensis

Taxon classificationAnimaliaHydrachnidiaTorrenticolidae

(K. Viets, 1930)

4E4B1F51-4C16-5CE2-9080-A88FF173CDB6

###### Material examined.

Portugal, **Guarda**: • Seia, Rio Alva, Praia Fluvial de Sabugueiro, river, 40.401°N, 7.64°W, 1021 m a.s.l., 24 Aug. 2023, leg. Ferreira, Benitez-Bosco & Padilha, 1♂ (sequenced); • Manteigas, Poço do Inferno, 40.373°N, 7.516°W, 1078 m a.s.l., 21 Aug. 2023, leg. Ferreira, Benitez-Bosco & Padilha, 1♀ (sequenced); • Manteigas, Poio do Leão, 40.399°N, 7.541°W, 734 m a.s.l., 22 Aug. 2023, leg. Ferreira, Benitez-Bosco & Padilha, 2♀ (sequenced). **Bragança** • Vinhais, Gasparona, 41.85°N, 7.013°W, 693 m a.s.l., 6 Jul. 2023, leg. Ferreira & Padilha, 1♀ (sequenced).

###### Remarks.

The Portuguese specimens match the description of *M.madritensis*. The specimens clustered within BOLD:AED3803, which includes specimens of *M.madritensis* from Montenegro, Serbia and Italy, available in BOLD.

###### Distribution.

Europe. New record for Portugal.

##### ﻿ Monatractides (Monatractides) stadleri

Taxon classificationAnimaliaHydrachnidiaTorrenticolidae

(Walter, 1924)

AE6EB570-604A-5824-A015-7501B7896400

###### Material examined.

Portugal, **Faro** • Monchique, Ribeira de Seixe, Parque do Barranco dos Pisões, stream, 37.333°N, 8.567°W, 23 May 2023, leg. Ekrem & Benitez-Bosco, 1♀ (sequenced). **Guarda**: • Gouveia, Rio Mondego, Casais de Folgosinho, 40.454°N, 7.493°W, 976 m a.s.l., 24 Aug. 2023, leg. Ferreira, Benitez-Bosco & Padilha, 1♀ (sequenced); • Seia, Rio Alva, Central hidroelétrica de Ponte dos Jugais, river, 40.385°N, 7.706°W, 555 m a.s.l., 24 Aug. 2023, leg. Ferreira, Benitez-Bosco & Padilha, 1♀ (sequenced); • Seia, Rio Alva, Praia fluvial de Vila Cova a Coelheira, river, 40.379°N, 7.736°W, 312 m a.s.l., 23 Aug. 2023, leg. Ferreira, Benitez-Bosco & Padilha, 1♂ (sequenced); • Seia, Casa do Loureiro, 40.433°N, 7.701°W, 415 m a.s.l., 19 July 2023 leg. Ferreira & Padilha, 1♀ (juv.) (sequenced).

###### Remarks.

The females used in this study for molecular analysis were clustered within BOLD:AEU1504, which includes two specimens of *M.stadleri* from Belgium, one specimen from Spain (identified as *Torrenticola* sp., deposited in Taxus Medio Ambiente, Spain), and one specimen recently collected from the stream in Beja Province and assigned to *M.stadleri* by Pešić et al. (2023b).

###### Distribution.

Europe. In Portugal previously reported from Corgo da Ponte Quebrada, Beja (Pešić et al. 2023b).

##### ﻿ Monatractides (Monatractides)

Taxon classificationAnimaliaHydrachnidiaTorrenticolidae

s tenostomus (K. Viets, 1930)

ACE14194-0BC6-56A6-A93B-AFA25E3059B0

###### Material examined.

Portugal, Beja • Mértola, Corte do Pinto, 37.682°N, 7.512°W, 19 May 2023, leg. Ferreira, Benitez-Bosco, Ekrem, Stur & Turaccio, 1♂ (sequenced), dissected and slide mounted (RMNH),

###### Remarks.

The Portuguese specimen molecularly analyzed in this study matches the description of *M.stenostomus*. This individual forms a unique BIN (BOLD:AFU3082), with the nearest neighboring BIN being BOLD:ADZ9854, which includes three specimens of an unidentified *Monatractides* sp. from Morocco, with the *p*-distance estimated at 6.74%.

###### Distribution.

Spain, France. In Portugal previously reported from Beira Alta (Santa Comba Dão; Lundblad 1956).

##### ﻿Genus Torrenticola

Taxon classificationAnimaliaHydrachnidiaTorrenticolidae

Piersig, 1896

832858C4-6453-5862-99D3-C212191F9CF5

###### Note.

So far 12 species of the genus were reported from Portugal, nine of them known from Madeira Island and four species from the mainland

##### ﻿ Torrenticola (Torrenticola) elliptica

Taxon classificationAnimaliaHydrachnidiaTorrenticolidae

Maglio, 1909

706B0B78-AAE0-54F0-A8FE-3B8B046CAC58

###### Material examined.

Portugal, **Bragança** • Vinhais, Gasparona, 41.85°N, 7.013°W, 693 m a.s.l., 6 Jul. 2023, leg. Ferreira & Padilha, 1♀ (sequenced).

###### Remarks.

The single Portuguese specimen molecularly analyzed in this study was a juvenile female that does not allow for a confident identification to the species level. Molecular data, however, revealed that the DNA barcode of the Portuguese specimen falls into BOLD:AEI9183, which includes one specimen from Montenegro, morphologically assigned by the first author to *T.elliptica*.

###### Distribution.

Palaearctic. New for Portugal.

##### ﻿ Torrenticola (Torrenticola) soniae

Taxon classificationAnimaliaHydrachnidiaTorrenticolidae

Pešić sp. nov.

C59EC9CA-BCE5-5F8B-B629-7DB467323F88

https://zoobank.org/E0A41268-B54C-4366-9886-E2954F1BF00B

Figs 1 , 2 , 5A

###### Type material examined.

***Holotype*** • ♂, dissected and slide mounted (RMNH), Portugal, **Guarda**, Seia, Rio Alva, Praia Fluvial de Sabugueiro (Fig. 5D), river, 40.401°N, 7.64°W, 1021 m a.s.l., 24 Aug. 2023, leg. Ferreira, Benitez-Bosco & Padilha, sequenced (BOLD ID: BSNTN961-23). ***Paratypes***: • 2♂ (sequenced), Portugal, **Guarda**, Seia, Rio Alva, Praia fluvial de Vila Cova a Coelheira, river, 40.379°N, 7.736°W, 312 m a.s.l., 23 Aug. 2023, leg. Ferreira, Benitez-Bosco & Padilha; • 1♂ (sequenced); Manteigas, Zêzere, Ponte dos Frades, 40.403°N, 7.526°W, 672 m a.s.l., 22 Aug. 2023, leg. Ferreira, Benitez-Bosco, Padilha, Andrade & Stur; • 1♀ (sequenced), dissected and slide mounted (RMNH), Guarda, Gouveia, Rio Mondego, Casais de Folgosinho, 40.454°N, 7.493°W, 976 m a.s.l., 24 Aug. 2023, leg. Ferreira, Benitez-Bosco & Padilha; • 1♀ (juv.; sequenced), **Bragança**, Mirandela, Torre de Dona Chama, 41.665°N, 7.146°W, 256 m a.s.l., 13 Jul. 2023, leg. Ferreira & Padilha.

###### Diagnosis.

***Morphological***: Cx-I relatively short, anteriorly broad; suture lines of Cx-IV prominent, starting at right angle from genital field; ejaculatory complex with well-developed anterior keel and proximal arms; gnathosomal rostrum short, less than width of gnathosoma; P-3 with a subrectangular, apically serrated ventrodistal projection. ***Molecular***: this lineage represented by a unique BIN (BOLD:AFW5337) differs from *T.brevirostris* clade by 12.27% K2P for COI.

###### Description.

**Figure 1.**
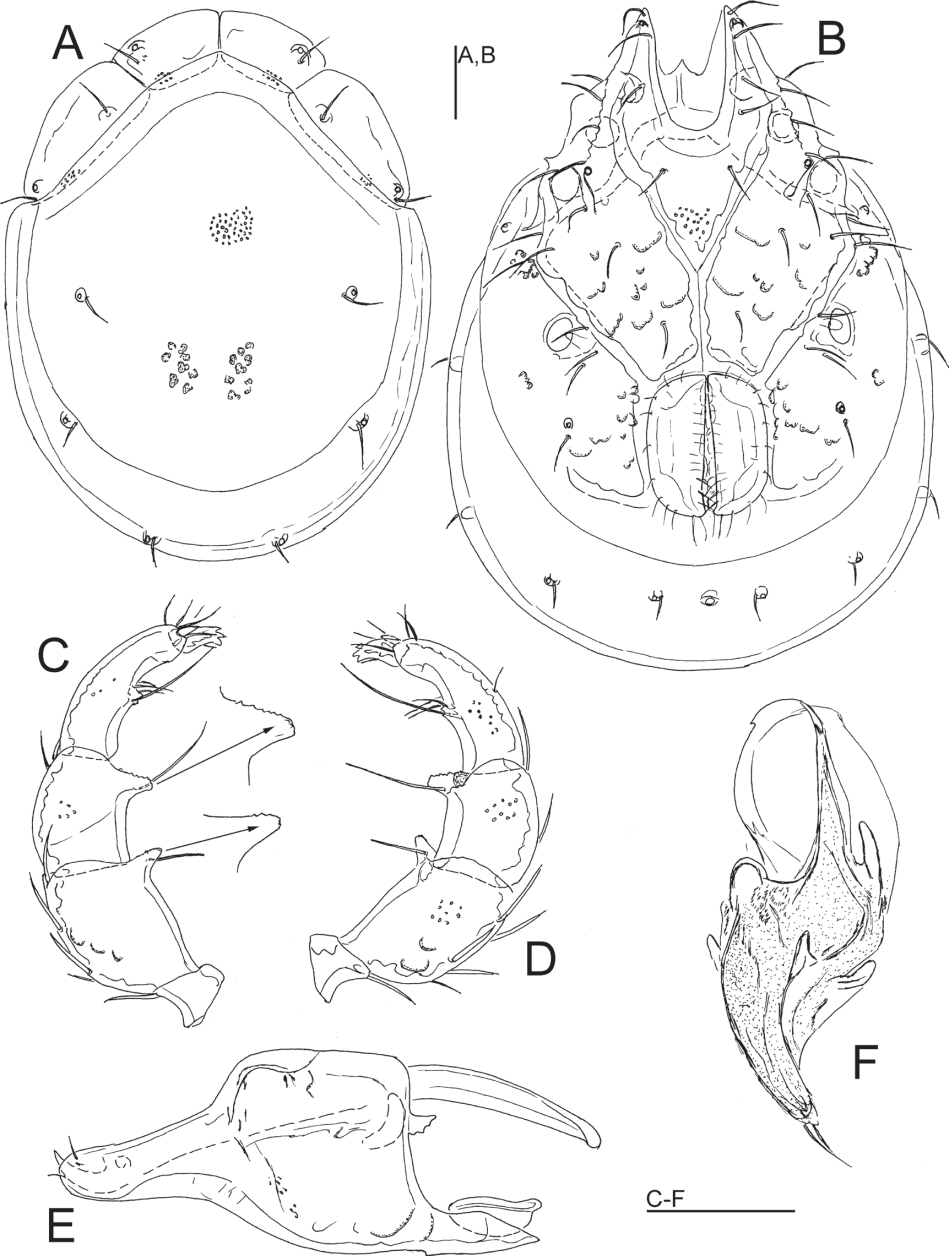
*Torrenticolasoniae* sp. nov., ♂ holotype **A** dorsal shield **B** ventral shield **C** palp, medial view (inset: ventrodistal projections of P-2 and P-3, enlarged 2×) **D** palp, lateral view **E** gnathosoma and chelicera **F** ejaculatory complex. Scale bars: 100 μm.

**Figure 2.**
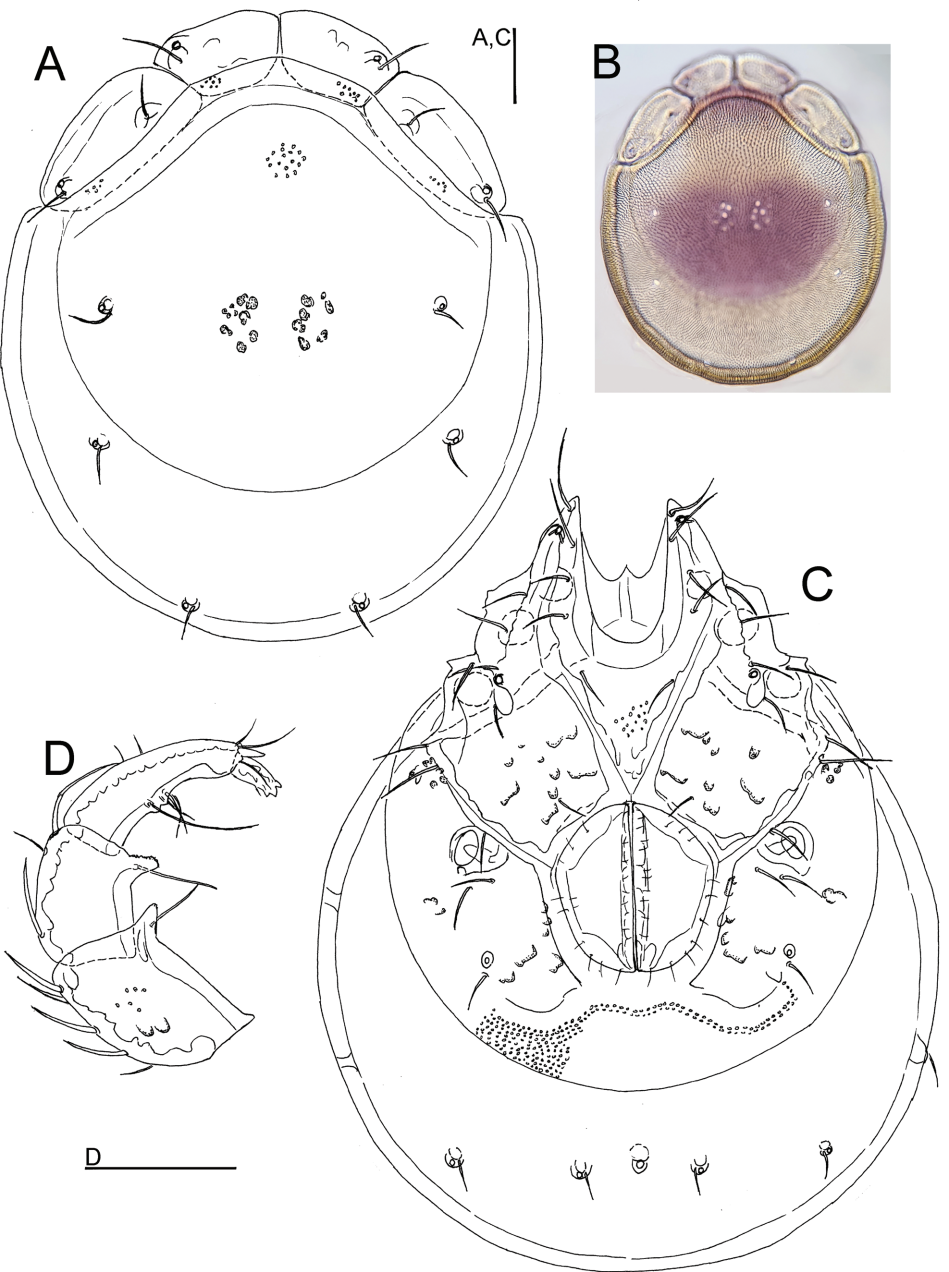
*Torrenticolasoniae* sp. nov., ♀ paratype **A** dorsal shield **B** photograph of dorsal shield **C** ventral shield **D** palp, medial view (P-1 lacking). Scale bars: 100 μm.

***General features.*** Idiosoma roundish; dorsal shield without a color pattern as photographed in Figs 2B, 5A; area of primary sclerotization of the dorsal plate with two dorsoglandularia (Fig. 1A); frontal platelets broad, relatively short; Cx-I relatively short, anteriorly broad; gnathosomal bay U-shaped, proximally rounded; Cxgl-4 subapical; medial suture line of Cx-II+III relatively short; postgenital area extended; excretory pore and Vgl-2 away from the line of primary sclerotization, excretory pore on the level of Vgl-2; gnathosomal rostrum short, less than depth of gnathosoma (Fig. 1E); P-2 ventral margin nearly straight or slightly convex, P-2 ventrodistal protrusion bluntly pointed, apically serrated, P-3 with a subrectangular, apically serrated ventrodistal projection, P-4 with a ventral tubercle bearing one long and three shorter setae (Fig. 1C, D). **Male.** Suture line of Cx-IV evident, medially starting from posterior margin of genital field in a right angle to the main idiosoma axis; genital field large, subrectangular; ejaculatory complex conventional in shape, anterior keel, proximal and distal arms well developed (Fig. 1F). **Female.** Genital field large and pentagonal in shape, suture lines of Cx-IV extending posteriorly beyond posterior margin of genital field, laterally curved.

###### Measurements.

**Male** (holotype). Idiosoma (ventral view: Fig. 1B) L 912, W 669; dorsal shield (Fig. 1A) L 756, W 581, L/W ratio 1.3; dorsal plate L 700; shoulder plate L 220–222, W 97, L/W ratio 2.27–2.29; frontal plate L 150–156, W 78–88, L/W ratio 1.8–1.9; shoulder/frontal plate L 1.42–1.47. Gnathosomal bay L 171, Cx-I total L 359, Cx-ImL 188, Cx-II+III mL 137; ratio Cx-I L/Cx-II+III mL 2.63; Cx-ImL/Cx-II+III mL 1.37. Genital field L/W 191/163, ratio 1.17; distance genital field-excretory pore 116, genital field-caudal idiosoma margin 209. Ejaculatory complex L 291.

Gnathosoma vL 331, chelicera L 375; palp total L 398, dL/H, dL/H ratio: P-1, 39/39, 1.0; P-2, 117/73, 1.59; P-3, 84/63, 1.35; P-4, 120/42, 2.86; P-5, 38/17, 2.18; L ratio P-2/P-4, 0.98. dL of I-L-4–6: 134, 150, 136; I-L-6 H 100; dL/H I-L-6 ratio 1.36.

**Female** (paratype from Casais de Folgosinho, BGE_00227_F02). Idiosoma (ventral view: Fig. 2C) L 1033, W 828; dorsal shield (Fig. 2A, B) L 844, W 725, L/W ratio 1.16; dorsal plate L 781; shoulder plate L 222–235, W 100–102, L/W ratio 2.2–2.3; frontal plate L 173–175, W 97–98, L/W ratio 1.79; shoulder/frontal plate L 1.28–1.34. Gnathosomal bay L 200, Cx-I total L 384, Cx-ImL 184, Cx-II+III mL 18; ratio Cx-I L/Cx-II+III mL 21.3; Cx-ImL/Cx-II+III mL 10.2. Genital field L/W 221/198, ratio 1.12; distance genital field-excretory pore 250, genital field-caudal idiosoma margin 391.

Gnathosoma vL 350, chelicera L 409; palp total L 435, dL/H, dL/H ratio: P-1, 44/41, 1.08; P-2, 127/76, 1.67; P-3, 93/65, 1.44; P-4, 134/42, 3.18; P-5, 37/18, 2.0; L ratio P-2/P-4, 0.94.

###### Etymology.

The species is dedicated to Sónia Ferreira (CIBIO, Portugal) for collecting numerous specimens used in this study and her enthusiastic support in the study of Portuguese water mites.

###### Species delimitation using DNA barcodes.

The final alignment for species delimitation using COI sequence data comprised 669 nucleotide positions (nps) of the 130 *Torrenticola* specimens listed in Suppl. material 2 and one outgroup, *Monatractidesmadritensis* from Portugal to root the tree. The NJ tree is presented in Fig. 6. The COI tree sequences retrieved from *Torrenticola* specimens from Portugal, here described as *T.soniae* sp. nov., appeared as a sister group to the cluster of sequences belonging to *T.brevirostris* (Halbert, 1911), a rhitrobiontic species widely distributed in Europe. The mean genetic distance between COI sequences of these two clusters was estimated at 12.27 ± 1.42% K2P. The genetic distance was considerably higher than the estimated barcode gap found by ASAP analyses (3–5%) of all studied *Torrenticola*, supporting the species-status of the new taxon. The mean intraspecific K2P-divergence within the cluster of the new species was 0.63 ± 0.19%.

###### Discussion.

With regards to the presence of an anteriorly broad and short Cx-I, a robust and compact palp, and a deep gnathosoma with a short rostrum, the new species resembles *T.brevirostris*. The latter species can be separated from *T.soniae* sp. nov. by only slightly protruding ventrodistal projections of P-2 and particularly of P-3.

###### Distribution.

Portugal (this study).

##### ﻿ Torrenticola (Torrenticola) elisabethae

Taxon classificationAnimaliaHydrachnidiaTorrenticolidae

Pešić sp. nov.

DF59C6E0-AC8B-5124-9C35-2001988D5B38

https://zoobank.org/354EB35B-1F5E-4FBB-9E8B-06B956A47467

Figs 3 , 4 , 5B, E

###### Type material examined.

***Holotype*** • ♂, dissected and slide mounted, Portugal, Guarda, Manteigas, Poço do Inferno (Fig. 5E), 40.373°N, 7.516°W, 1078 m a.s.l., 21 Aug. 2023, leg. Ferreira, Benitez-Bosco & Padilha, sequenced (BOLD ID: BSNTN984-23). ***Paratypes***: • 1♂, 1♀ (sequenced), Portugal, **Guarda**, Manteigas, Poio do Leão, 40.399°N, 7.541°W, 734 m a.s.l., 22 Aug. 2023, leg. Ferreira, Benitez-Bosco & Padilha, 1♀ dissected and slide mounted (RMNH).

###### Diagnosis.

***Morphological***: Shoulder platelets fused with dorsal plate; dorsal shield with color pattern as illustrated in Figs 4B, 5B; Cxgl–4 subapical; medial suture line of Cx-II+III in male relatively long; ejaculatory complex with poorly developed anterior keel and a relatively large proximal chamber. ***Molecular***: this lineage represent by a unique BIN (BOLD:AFW5336) differs from *T.lundbladi* clade by 9.8% K2P for COI.

###### Description.

**Figure 3.**
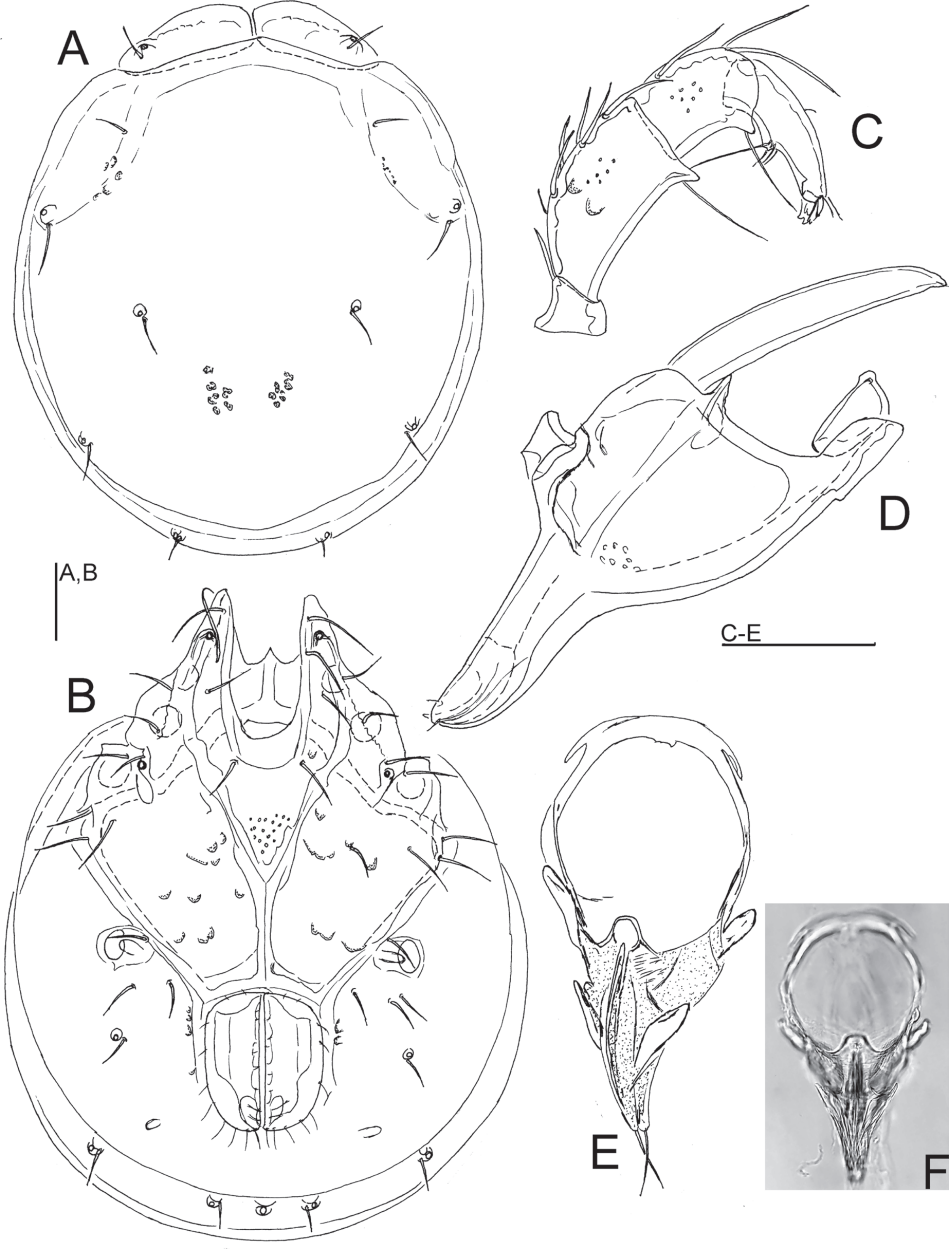
*Torrenticolaelisabethae* sp. nov., ♂ holotype **A** dorsal shield **B** ventral shield C palp, medial view **D** gnathosoma and chelicera **E** ejaculatory complex **F** photograph of ejaculatory complex. Scale bars: 100 μm.

**Figure 4.**
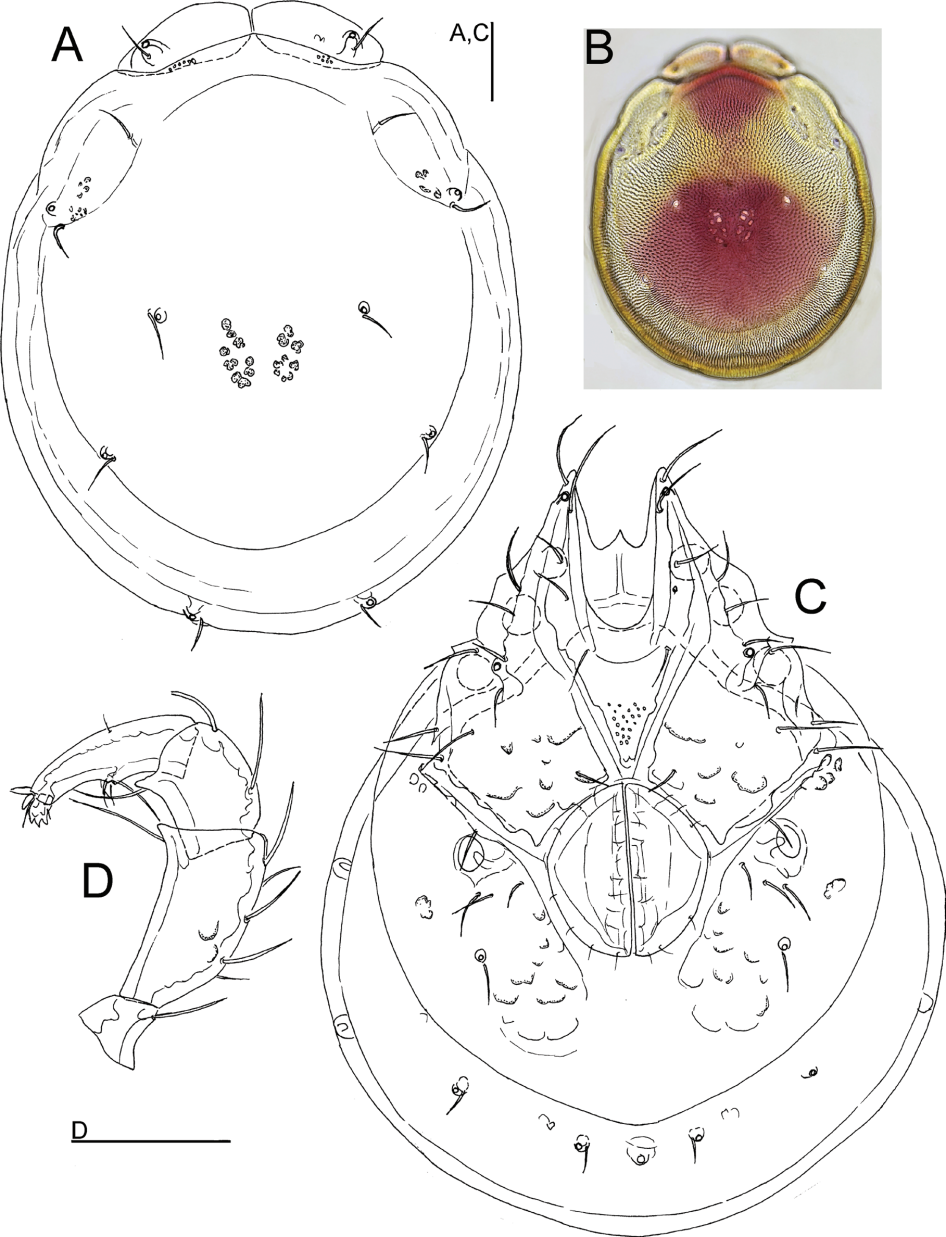
*Torrenticolaelisabethae* sp. nov., ♀ paratype **A** dorsal shield **B** photograph of dorsal shield **C** ventral shield **D** palp, medial view. Scale bars: 100 μm.

**Figure 5.**
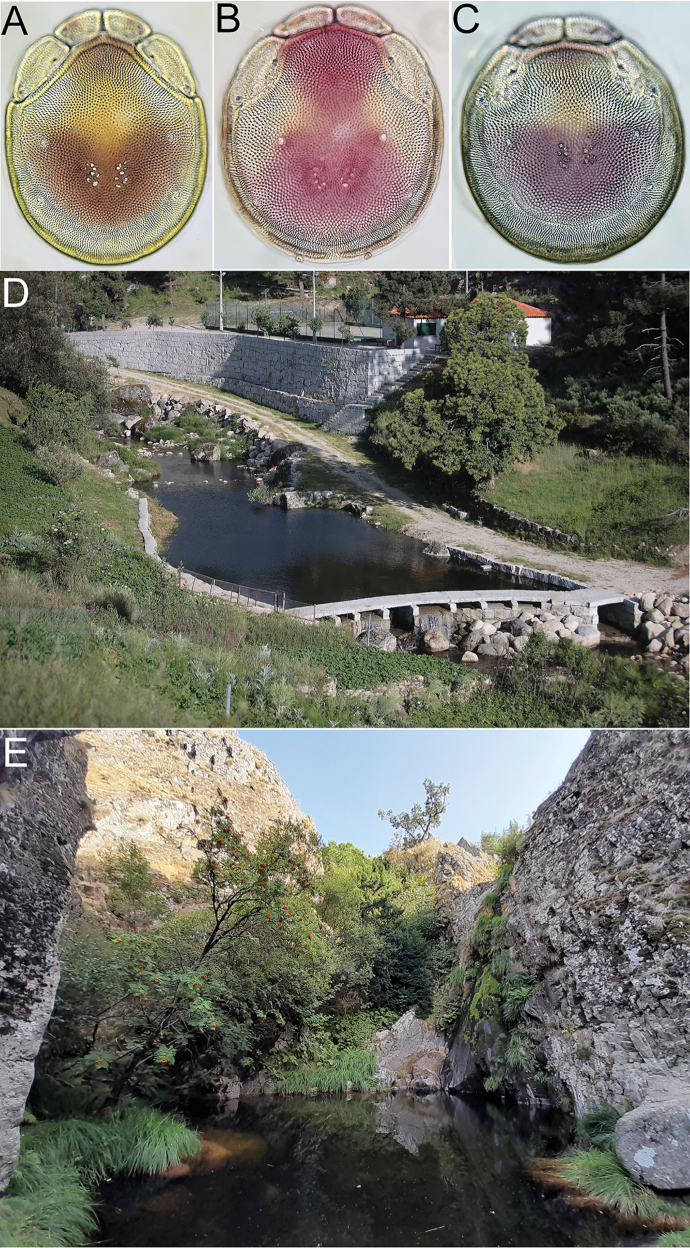
**A–C** Photographs of dorsal shield **A***Torrenticolasoniae* sp. nov., ♂ holotype **B***T.elisabethae* sp. nov., ♂ holotype **C***T.tenuipalpis*, ♀ (BGE_00227_F03) **D–E** Photographs of selected sampling sites **D** Praia Fluvial de Sabugueiro, *locus typicus* of *Torrenticolasoniae* sp. nov. **E** Poço do Inferno, type locality of *T.elisabethae* sp. nov. Photographs by JC (5D) and SF (5E).

***General features.*** Idiosoma oval; shoulder platelets fused to dorsal plate, but suture line visible; dorsal shield with a color pattern as illustrated in Figs 4B, 5B; area of primary sclerotization of the dorsal plate with four dorsoglandularia (Fig. 3A); gnathosomal bay U-shaped, proximally rounded; Cxgl–4 subapical; excretory pore and Vgl-2 on the the line of primary sclerotization, excretory pore on the level of Vgl-2; gnathosomal ventral margin curved, rostrum elongated (Fig. 3D); P-2 ventral margin nearly straight or slightly concave, P-2 and P-3 ventrodistal protrusions bluntly pointed, P-4 with a ventral tubercle bearing one long and three shorter setae (Figs 3C, 4D). **Male** — Medial suture line of Cx-II+III relatively long; genital field subrectangular; ejaculatory complex with poorly developed anterior keel, proximal chamber relatively large; Fig. 3E). **Female** — Genital field large and pentagonal in shape.

**Figure 6.**
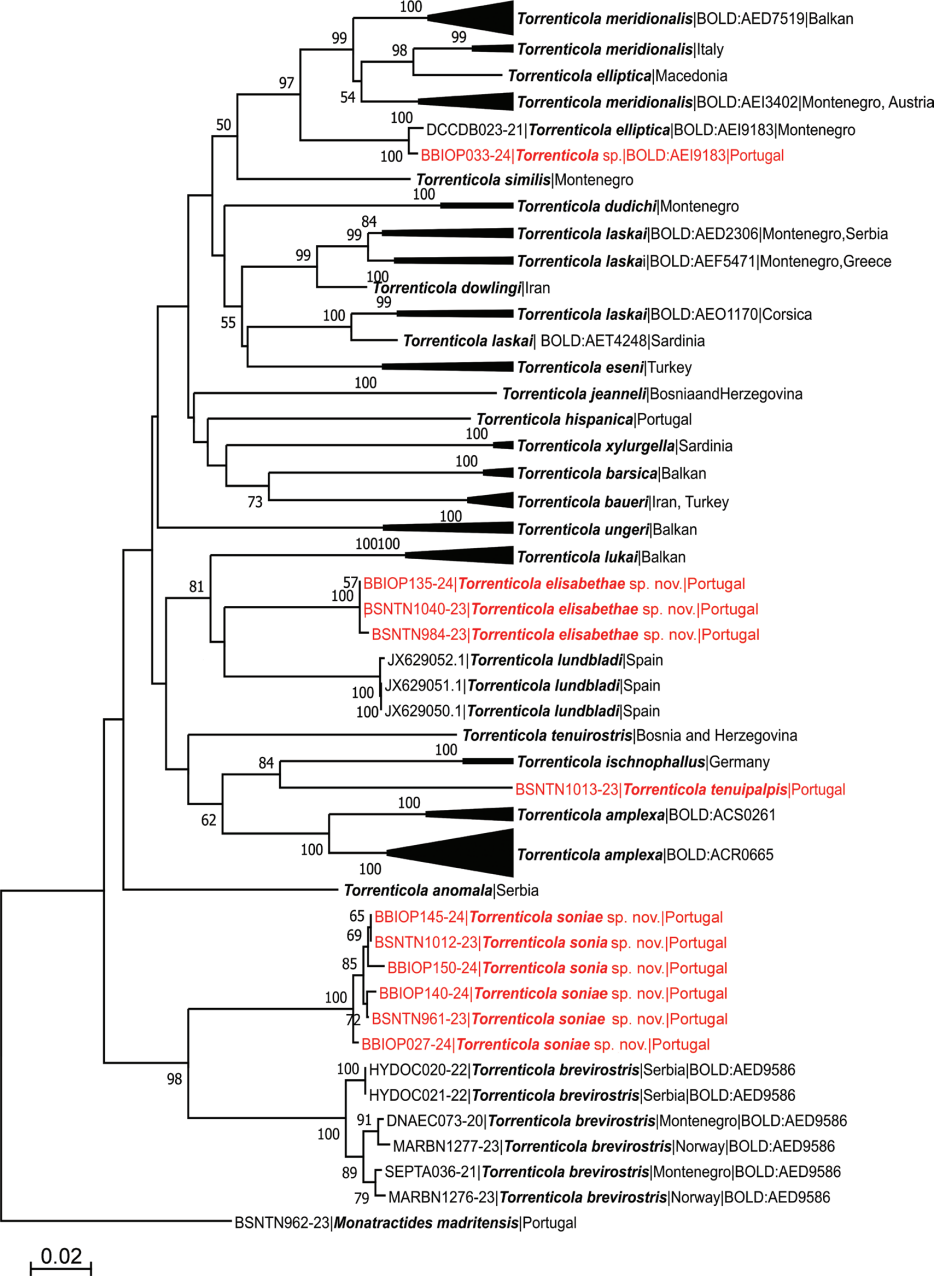
Neighbor-Joining tree of the genus *Torrenticola*, obtained from 130 nucleotide COI sequences. Bootstrap values > 50% from 1000 bootstrap replicates on branches.

###### Measurements.

**Male** (holotype). Idiosoma (ventral view: Fig. 3B) L 856, W 691; dorsal shield (Fig. 3A) L 731, W 619, L/W ratio 1.18; dorsal plate L 681; frontal plate L 173–183, W 64–66, L/W ratio 2.7–2.8. Gnathosomal bay L 194, Cx-I total L 383, Cx-ImL 188, Cx-II+III mL 131; ratio Cx-I L/Cx-II+III mL 2.92; Cx-ImL/Cx-II+III mL 1.43. Genital field L/W 183/150, ratio 1.22; distance genital field-excretory pore 103, genital field-caudal idiosoma margin 127. Ejaculatory complex L 275.

Gnathosoma vL 367, chelicera L 448; palp total L 390, dL/H, dL/H ratio: P-1, 44/38, 1.17; P-2, 133/64, 2.08; P-3, 79/58, 1.36; P-4, 112/38, 2.98; P-5, 22/14, 1.55; L ratio P-2/P-4, 1.19. dL of I-L-4–6: 145, 160, 131; I-L-6 H 46; dL/H I-L-6 ratio 2.85.

**Female** (paratype from Poio do Leão, BGE_00227_H06). Idiosoma (ventral view: Fig. 4C) L 975, W 794; dorsal shield (Fig. 4A, B) L 806, W 663, L/W ratio 1.22; dorsal plate L 766; frontal plate L 172–175, W 63–68, L/W ratio 2.6–2.75. Gnathosomal bay L 203, Cx-I total L 391, Cx-ImL 188, Cx-II+III mL 0. Genital field L/W 214/204, ratio 1.05; distance genital field-excretory pore 256, genital field-caudal idiosoma margin 347. Egg (*n* = 1) maximum diameter 227.

Gnathosoma vL 379, chelicera L 478; palp total L 389, dL/H, dL/H ratio: P-1, 41/36, 1.15; P-2, 130/64, 2.0; P-3, 80/59, 1.35; P-4, 116/40, 2.87; P-5, 22/14, 1.55; L ratio P-2/P-4, 1.13.

###### Etymology.

The new species is dedicated to Elisabeth Stur (NTNU University Museum Trondheim, Norway), who facilitated a number of barcoding projects on water mites in Europe.

###### Species delimitation using DNA barcodes.

The sequences retrieved from *Torrenticola* specimens from Portugal, here described as *T.elisabethae* sp. nov., appeared as a sister group to the cluster containing sequences of *T.lundbladi* (K. Viets, 1930), a rhitrobiontic species known from Spain (Lundblad 1956; Pešić et al. 2012). The mean K2P genetic distance between COI sequences of *T.elisabethae* sp. nov. and *T.lundbladi* was estimated at 9.8 ± 1.25%. The genetic distance was also here higher than the barcode gap found for *Torrenticola* in the ASAP analysis, supporting the species-status of the new taxon. The mean intraspecific divergence within the cluster of barcodes belonging to *T.elisabethae* was relatively low (0.2 ± 0.14% K2P).

###### Discussion.

The new species is most similar to *Torrenticolalundbladi* K. Viets, 1930, a species originally described from central Spain (K. Viets 1930). Both species have dorsal shield with the shoulder platelets partially fused with the dorsal plate, a similar color pattern of the dorsal shield, a Cxgl-4 situated subapically and a relatively long median suture line of Cx-II-III in male. *Torrenticolalundbladi* differs by the characteristic shape of the ejaculatory complex (proximal and distal arms short, proximal chamber large, proximal horns reduced, see Lundblad 1956: fig. 83E).

###### Distribution.

Portugal (this study).

##### ﻿ Torrenticola (Torrenticola) tenuipalpis

Taxon classificationAnimaliaHydrachnidiaTorrenticolidae

Lundblad, 1956

C5BED193-FAAB-5CA5-B8D6-547CB9B57832

Fig. 5C

###### Material examined.

Portugal, **Guarda** • Gouveia, Rio Mondego, Casais de Folgosinho, 40.454°N, 7.493°W, 976 m a.s.l., 24 Aug. 2023, leg. Ferreira, Benitez-Bosco & Padilha, 1♀ (sequenced), dissected and slide mounted (RMNH).

###### Remarks.

The Portuguese specimen matches the description of *Torrenticolatenuipalpis*, a species originally described by Lundblad (1956) as a “*variatio*” of *T.amplexa* based on a single female collected in a stream in Santa Comba Dão, Portugal. Recently, Cantallo et al. (2022) ranked the latter taxon as a distinct species. The sequenced specimen from Portugal forms a unique BIN (BOLD:AFV2021) with the nearest neighboring BIN (*p*-distance 12.2%) being BOLD:AFF4076, which consists of a single specimen of *T.ramini* from Iran.

###### Distribution.

Portugal; known from Beira Alta (Lundblad 1956).

#### ﻿Family Limnesiidae Thor, 1900

##### ﻿Genus Limnesia

Taxon classificationAnimaliaHydrachnidiaLimnesiidae

Koch, 1836

7A358908-2576-5B84-BDEC-E2CF540862F2

###### Note.

In Portugal, represented by eight species, seven of them known from the mainland and one (*L.atlantica* Lundblad, 1941) known only from Madeira.

##### ﻿ Limnesia (Limnesia) acuminata

Taxon classificationAnimaliaHydrachnidiaLimnesiidae

Walter, 1925

25443953-57F8-554D-B13E-55040C159661

###### Material examined.

Portugal, **Beja** • Mértola, Moinho de Alferes 1, 37.502°N, 7.69°W, 19 May 2023, leg. Ferreira, Benitez-Bosco, Ekrem, Stur & Turaccio, 1♂ (sequenced). **Bragança**: • Mirandela, Torre de Dona Chama, 41.665°N, 7.146°W, 256 m a.s.l., 13 Jul. 2023, leg. Ferreira & Padilha, 2♂, 1♀ (sequenced).

###### Remarks.

The Portuguese specimens match the description of *L.acuminata*. Genetic data indicate that specimens from Portugal form a unique cluster (BOLD:AFU7587).

###### Distribution.

Western Mediterranean (Iberian Peninsula, southern France, Sardinia, Sicily, north Africa). In Portugal previously reported from Beira Alta, Alentejo, and Estremadura (Lundblad 1956).

##### ﻿ Limnesia (Limnesia) iberica

Taxon classificationAnimaliaHydrachnidiaLimnesiidae

Lundblad, 1954

1C80227E-5DD4-57E9-A2A4-54F4098F6862

###### Material examined.

Portugal, **Beja** • Mértola, São João dos Caldeireiros, stream, 37.626°N, 7.81°W, 17 May 2023, leg. Ferreira, Benitez-Bosco, Ekrem, Stur & Turaccio, 3♂, 6♀ (sequenced).

###### Remarks.

The specimens from Portugal morphologically matches description of *Limnesiaiberica*, a species originally described by Lundblad (1956) from a stream in Beira Alta (Santa Comba Dão, Portugal). The Portuguese specimens form a distinct BIN (BOLD:AFN8367) with the closest BIN being BOLD:ACA9272, which includes specimens from Canada, United States, and Greece assigned to *L.undulata*, with the *p*-distance estimated at 14.29%.

###### Distribution.

Portugal (Lundblad 1954, 1956; this study).

##### ﻿ Limnesia (Limnesia) koenikei

Taxon classificationAnimaliaHydrachnidiaLimnesiidae

Piersig, 1894

065C2D53-D9CB-598C-991F-7F8630ECA192

###### Material examined.

Portugal, **Guarda**: • Seia, Covão do Forno, 40.369°N, 7.638°W, 1574 m a.s.l., 19 Aug. 2023, leg. Ferreira, Benitez-Bosco & Padilha, 1♂, 2♀ (sequenced); • Seia, Rio Alva, Central hidroelétrica de Ponte dos Jugais, river, 40.385°N, 7.706°W, 555 m a.s.l., 24 Aug. 2023, leg. Ferreira, Benitez-Bosco & Padilha, 1♂ (sequenced).

###### Remarks.

The sequences obtained from the specimens from Portugal cluster within BOLD:ADF6559, which includes one specimen from the Netherlands assigned to *L.koenikei*. The *p*-distance from the latter BIN and its nearest neighbor BOLD:ACS0816, which includes specimens of *L.koenikei* from Norway and the Netherlands, was estimated at 2.09%.

###### Distribution.

Holarctic; widely distributed in Europe but here reported for the first time for Portugal.

##### ﻿ Limnesia (Limnesia) maculata

Taxon classificationAnimaliaHydrachnidiaLimnesiidae

(Müller, 1776)

FC4C88D1-7F6A-5B00-9176-3A0A6398E9A7

###### Material examined.

Portugal, **Guarda**: • Seia, Rio Alva, Praia Fluvial de Sabugueiro, river, 40.401°N, 7.64°W, 1021 m a.s.l., 24 Aug. 2023, leg. Ferreira, Benitez-Bosco & Padilha, 1♀ (sequenced). **Beja** • Mértola, Moinho de Alferes 2, 37.503°N, 7.687°W, 19 May 2023, leg. Ferreira, Benitez-Bosco, Ekrem, Stur & Turaccio, 1♂ (juv.) (sequenced); • Mértola, Herdade de Alagães, pond, 37.673°N, 7.848°W, 18 May 2023, leg. Ferreira, Benitez-Bosco, Ekrem, Stur & Turaccio, 1♂, 1♀ (sequenced).

###### Remarks.

The Portuguese specimens molecularly analyzed in this study match the description of *L.maculata*. These individuals form a unique BIN (BOLD:AFW6935), with the nearest neighboring BIN being BOLD:ACS0248, which includes specimens of *L.maculata* from Norway, the Netherlands and France. The *p*-distance between these two clusters was estimated at 4.3%.

###### Distribution.

Holarctic. Widespread in Europe. In Portugal previously reported from Alentejo (Ribeira de Odivelas; Lundblad 1956).

##### ﻿ Limnesia (Limnesia) walteri

Taxon classificationAnimaliaHydrachnidiaLimnesiidae

Migot, 1926

95B31EFA-E9A6-5A47-8A07-7173155E6B10

###### Material examined.

Portugal, **Beja** • Mértola, Corte do Pinto, 37.682°N, 7.512°W, 19 May 2023, leg. Ferreira, Benitez-Bosco, Ekrem, Stur & Turaccio, 3♀ (sequenced). **Bragança** • Mirandela, Torre de Dona Chama, 41.665°N, 7.146°W, 256 m a.s.l., 13 Jul. 2023, leg. Ferreira & Padilha, 1 ♀ (sequenced).

###### Remarks.

The sequenced specimens from Portugal form a unique BIN (BOLD:AFO9873) with the nearest neighboring BIN being BOLD:ADZ9059 (*p*-distance 1.6%), which includes four unidentified *Limnesia* specimens from Morocco.

###### Distribution.

North Africa, including the Sahara desert, Russia, southwestern Europe from Portugal (Lundblad 1956; Valdecasas 1988) to Greece (Gerecke et al. 2016). In Portugal previously reported from Beira Alta (Lundblad 1956).

#### ﻿Family Hygrobatidae Koch, 1842

##### ﻿Genus Atractides

Taxon classificationAnimaliaHydrachnidiaHygrobatidae

Koch, 1837

1590E703-3669-5EFF-B277-2D95E27DEB52

###### Note.

Nine species known from Portugal, five of them endemic to Madeira, and *A.marizae* Pešić, 2023 endemic to mainland Portugal

##### ﻿ Atractides (Atractides) inflatus

Taxon classificationAnimaliaHydrachnidiaHygrobatidae

(Walter, 1925)

A36D900C-8524-5170-B6C2-14E394101AE7

###### Material examined.

Portugal, **Beja** • Odemira, Ribeira de Seixe, Herdade do Vale de Águia, river, 37.398°N, 8.68°W, 75 m a.s.l., 23 May 2023, leg. Ekrem & Benitez-Bosco, 2♀ (sequenced). **Vila Real** • Murça, Noura stream, 41.409°N, 7.417°W, 421 m a.s.l., 12 Jul. 2023 leg. Ferreira & Padilha, 1♀ (sequenced).

###### Remarks.

The specimens from Portugal used in this study match the description of *A.inflatus*, a species widely distributed in the Mediterranean region, often very frequent in intermittent streams (Pešić et al. 2023c). The Portuguese specimens were clustered within two BINs: BOLD:AFI9009, which includes two specimens of *A.inflatus* from Italy, and BOLD:ACB4677, which includes specimens of *A.inflatus* from Iran, Morocco, Montenegro, Turkey, Greece, France, and Italy. The *p*-distance between these two BINs was estimated at 6.06%.

###### Distribution.

Mediterranean, Iran. New for Portugal.

##### ﻿ Atractides (Atractides) marizae

Taxon classificationAnimaliaHydrachnidiaHygrobatidae

Pešić, 2023

A3DBEFBD-1A5B-5A6D-ADC3-C376AEE9DC71

###### Material examined.

Portugal, **Guarda**: • Gouveia, Rio Mondego, Casais de Folgosinho, 40.454°N, 7.493°W, 976 m a.s.l., 24 Aug. 2023, leg. Ferreira, Benitez-Bosco & Padilha, 1♀ (sequenced); • Seia, Rio Alva, Praia fluvial de Vila Cova a Coelheira, river, 40.379°N, 7.736°W, 312 m a.s.l., 23 Aug. 2023, leg. Ferreira, Benitez-Bosco & Padilha, 1♀ (sequenced). **Faro**: • Monchique, Caldas de Monchique, 37.287°N, 8.554°W, 23 May 2023, leg. Ekrem & Benitez-Bosco, 1♀ (sequenced); • Aljezur, Ribeira de Seixe, Odeceixe, Covão da Serva, 37.374°N, 8.642°W, 100 m a.s.l., 23 May 2023, leg. Ekrem & Benitez-Bosco 1♀ (sequenced); • Portimão, 37.237°N, 8.546°W, 23 May 2023, leg. Ferreira & Turaccio 1♂ (sequenced).

###### Remarks.

The specimens from Portugal clustered within BOLD:AER7878, which includes specimens of *Atractidesmarizae* Pešić, 2023, a species recently described by Pešić et al. (2023b) from Santarém, Portugal. Until now, this rhitrobiontic species was known only from the type locality (Caniceira stream), and the new findings presented in this study demonstrate that *A.marizae* is more widely distributed in Portugal.

###### Distribution.

Portugal.

##### ﻿ Atractides (Atractides) nodipalpis

Taxon classificationAnimaliaHydrachnidiaHygrobatidae

(Thor, 1899)

D34310F3-0C4F-5B23-B16B-9C7255EDD942

###### Material examined.

Portugal, **Guarda** • Seia, Rio Alva, Praia fluvial de Vila Cova a Coelheira, river, 40.379°N, 7.736°W, 312 m a.s.l., 23 Aug. 2023, leg. Ferreira, Benitez-Bosco & Padilha, 1♀ (sequenced).

###### Remarks.

The female, which keyed to *A.nodipalpis* following Gerecke et al. (2016), forms a unique BIN (BOLD:AFV2009). The BIN is placed as sister to BOLD:ACR0209, which includes > 200 specimens of *A.nodipalpis*, available in the BOLD database. The *p*-distance between these two BINs was estimated at 4.61%.

For a long time, *A.nodipalpis* has been considered the most common species of the genus in Europe. However, in the last years, genetic data revealed that the latter species consists of several distinct lineages, some of them present in the same areas (Gerecke et al. 2022; Pešić et al. 2023d). For example, Gerecke et al. (2022) mentioned that Norwegian specimens keyed as *A.nodipalpis* belong to two different lineages, both widely distributed in Norway. The taxonomic status of most of these lineages is still unclear as a number of species have been proposed as synonyms of *A.nodipalpis* in the past. Nevertheless, Gerecke and collaborators (Gerecke pers. comm. 2022) recently clarified the correct BIN assignment of the true *A.nodipalpis* lineage. They found that specimens of *A.nodipalpis* collected near its type locality in Norway belong to the BOLD:ACR0209 cluster. After that, Pešić et al. (2023d) examined specimens from the Netherlands belonging to BOLD:ACR0209 and found that *A.nodipalpis* can be defined primarily by the shape of male genital plate which has a distinct anteromedial peg-like fissure.

###### Distribution.

Based on the available records in BOLD, *A.nodipalpis* has a wide distribution, from SE Europe over the Fennoscandia up to Greenland. In Portugal previously reported from Beira Alta (Santa Comba Dão; Lundblad 1956).

##### ﻿ Atractides (Atractides) robustus

Taxon classificationAnimaliaHydrachnidiaHygrobatidae

(Sokolow, 1940)

35AC45CC-0750-52D2-8B57-8CD0F54EDC48

###### Material examined.

Portugal, **Guarda** • Manteigas, Zêzere, Covão da Ametade, 40.328°N, 7.587°W, 1431 m a.s.l., 21 Aug. 2023, leg. Ferreira, Benitez-Bosco & Padilha, 1♀ (sequenced).

###### Remarks.

The Portuguese specimen matches the description of *A.robustus*. The specimen clusters within BOLD:AFF2463, which includes specimens from Italy, Albania and Poland. The *p*-distance from the closest neighboring BIN being BOLD:ADZ9348, which consists of specimens of *A.robustus* from Germany, Austria, Montenegro, Romania, Italy, Bosnia and Herzegovina, Albania, and Greece, was estimated at 3.15%.

Recently, Pešić et al. (2023d) showed that *A.robustus*, a species originally described from the Caucasus (the affluents of the Kuban River), consists of two distinct lineages, one which includes populations from eastern Turkey and northern Iran, and which is likely conspecific with *A.robustus*, and the second lineage, which includes *A.robustus* like specimens from central and southern Europe. The latter lineage, to which BOLD:ADZ9348 and BOLD:AFF2463 belong, possible represents a cryptic species new to science. The final decision on the taxonomic status of *A.robustus* lineages has been postponed until material of the latter species from the Caucasus is available (Pešić et al. 2023d).

###### Distribution.

Europe. New for Portugal.

##### ﻿Genus Hygrobates

Taxon classificationAnimaliaHydrachnidiaHygrobatidae

Koch, 1837

A67D6A75-8E04-51ED-B7FC-B549106BDDAF

###### Note.

Five species known from mainland part of Portugal.

##### ﻿ Hygrobates balcanicus

Taxon classificationAnimaliaHydrachnidiaHygrobatidae

Pešić, 2020

F91BA968-76FD-5138-81C7-F8D6EA33343B

###### Material examined.

Portugal, **Faro** • Portimão, stream, 37.237°N, 8.546°W, 23 May 2023, leg. Ferreira & Turaccio, 2♂, 1♀ (sequenced). **Porto** • Lousada, Moinho da Tapada, 41.263°N, 8.307°W, 176 m a.s.l., 1 Sep. 2023, Ferreira, Sousa, Cruz-Oliveira & Girão, 1♂ (sequenced); • Lousada, Parque Molinológico e Florestal de Pias, 41.268°N, 8.256°W, 170 m a.s.l., 1 Sep. 2023, leg. Ferreira, Sousa, Cruz-Oliveira & Girão, 1♂ (sequenced); • Vila do Conde, Rio Este, 41.378°N, 8.695°W, 15 m a.s.l., 7 Sep. 2023, leg. Ferreira, Cruz-Oliveira & Girão, 3♂, 1 deutonymph (sequenced).

###### Remarks.

The specimens from Portugal morphologically match the description of *Hygrobatesbalcanicus*. This species was originally described by Pešić et al. (2020) from Bulgaria, and later on reported from eastern Serbia (Pešić et al. 2023a). The sequenced specimens from Portugal cluster within BOLD:AEG3198, which, in addition to the specimens used in this study, includes specimens from Bulgaria, Serbia and Italy morphologically assigned to *H.balcanicus*.

###### Distribution.

Balkans, Italy. New for Portugal.

##### ﻿ Hygrobates fluviatilis

Taxon classificationAnimaliaHydrachnidiaHygrobatidae

(Ström, 1768)

AA965F92-BFAD-5593-BA59-A12C335FC6B6

###### Material examined.

Portugal, **Guarda**: • Manteigas, Casa do Cantoneiro, 40.418°N, 7.603°W, 1378 m a.s.l., 24 Aug. 2023, leg. Ferreira, Benitez-Bosco & Padilha, 1♂ (sequenced); • Gouveia, Rio Mondego, Casais de Folgosinho, 40.454°N, 7.493°W, 976 m a.s.l., 24 Aug. 2023, leg. Ferreira, Benitez-Bosco & Padilha, 1♀ (sequenced); • Seia, Rio Alva, Praia fluvial de Vila Cova a Coelheira, river, 40.379°N, 7.736°W, 312 m a.s.l., 23 Aug. 2023, leg. Ferreira, Benitez-Bosco & Padilha, 2♂ (sequenced); • Seia, Rio Alva, Praia Fluvial de Sabugueiro, river, 40.401°N, 7.64°W, 1021 m a.s.l., 24 Aug. 2023, leg. Ferreira, Benitez-Bosco & Padilha, 1♂ (sequenced).

###### Remarks.

Genetic data indicate that Portuguese specimens belong to BOLD:ACB4846, which includes more than 300 specimens of *H.fluviatilis*, available in BOLD. The latter species was recently revised using molecular and morphological data (Pešić et al. 2017).

###### Distribution.

Central, western, and southern Europe. In Portugal previously reported from Santa Comba Dão (Beira Alta; Lundblad 1956).

##### ﻿ Hygrobates longiporus

Taxon classificationAnimaliaHydrachnidiaHygrobatidae

Thor, 1898 complex

B9105C9F-1D02-5007-A1F4-C88F5C49A642

Fig. 7

###### Material examined.

Portugal, **Guarda**: • Seia, Rio Alva, Praia fluvial de Vila Cova a Coelheira, river, 40.379°N, 7.736°W, 312 m a.s.l., 23 Aug. 2023, leg. Ferreira, Benitez-Bosco & Padilha, 1♀, 1 deutonymph (sequenced); • Seia, Rio Alva, Praia Fluvial de Sabugueiro, river, 40.401°N, 7.64°W, 1021 m a.s.l., 24 Aug. 2023, leg. Ferreira, Benitez-Bosco & Padilha, 2♂, 1♀, 1 deutonymph (sequenced); • Manteigas, Poço do Inferno, 40.373°N, 7.516°W, 1078 m a.s.l., 21 Aug. 2023, leg. Ferreira, Benitez-Bosco & Padilha, 1♂, 1♀ (sequenced); • Manteigas, Mondego, Covão da ponte, 40.443°N, 7.514°W, 999 m a.s.l., 24 Aug. 2023, leg. Ferreira, Benitez-Bosco & Padilha, 1♂, 3♀, 2 deutonymph (sequenced); • Gouveia, Rio Mondego, Casais de Folgosinho, 40.454°N, 7.493°W, 976 m a.s.l., 24 Aug. 2023, leg. Ferreira, Benitez-Bosco & Padilha, 1♂, 1♀ (sequenced); • Manteigas, Poio do Leão, 40.399°N, 7.541°W, 734 m a.s.l., 22 Aug. 2023, leg. Ferreira, Benitez-Bosco & Padilha, 1♂ (sequenced); • Manteigas, Zêzere, Ponte dos Frades, 40.403°N, 7.526°W, 672 m a.s.l., 22 Aug. 2023, leg. Ferreira, Benitez-Bosco, Padilha, Andrade & Stur 1♂ (sequenced); • Seia, Rio Alva, Central hidroelétrica de Ponte dos Jugais, river, 40.385°N, 7.706°W, 555 m a.s.l., 23 Aug. 2023, leg. Ferreira, Benitez-Bosco & Padilha, 1♀ (sequenced); • Rio Alva, Nossa Senhora do Desterro, river, 40.395°N, 7.694°W, 791 m a.s.l., 23 Aug. 2023, leg. Ferreira, Benitez-Bosco & Padilha, 1♀ (sequenced). **Bragança**: • Mirandela, Torre de Dona Chama, 41.665°N, 7.146°W, 256 m a.s.l., 13 Jul. 2023, leg. Ferreira & Padilha, 1 ♀ (sequenced). **Vila Real** • Murça, Noura stream, 41.409°N, 7.417°W, 421 m a.s.l., 12 Jul. 2023 leg. Ferreira & Padilha, 1♂ (sequenced).

###### Remarks.

**Figure 7.**
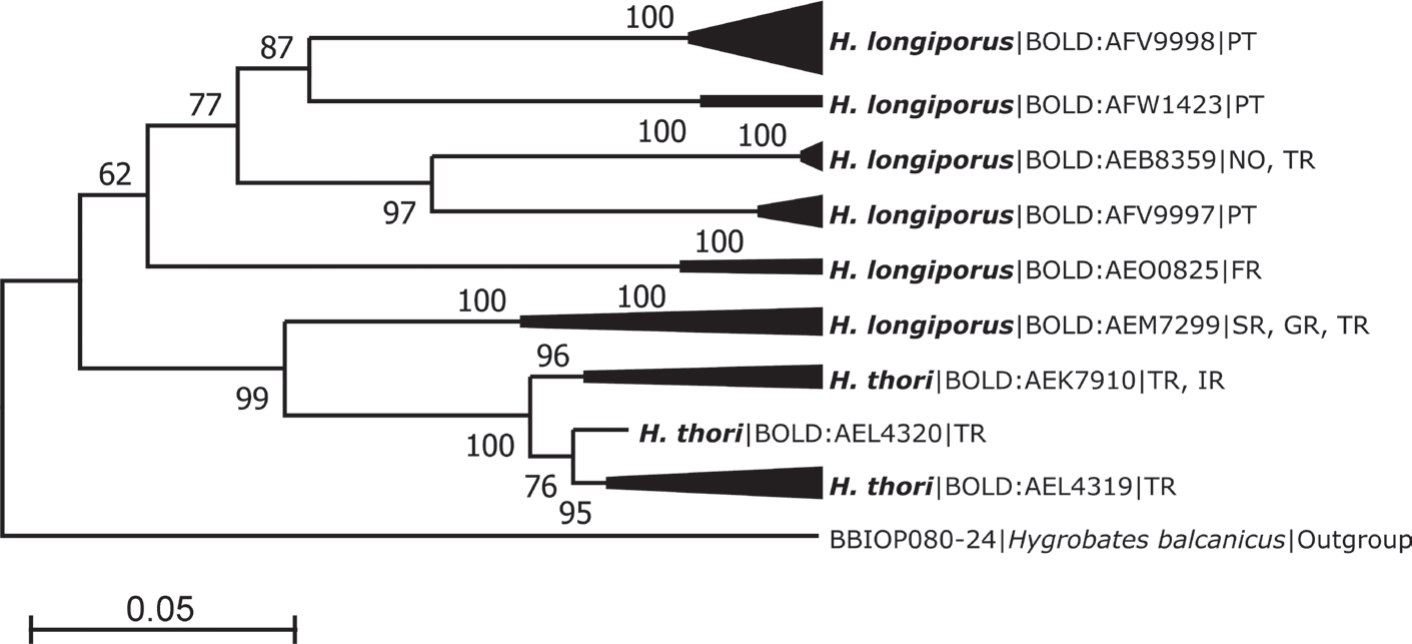
Neighbor-Joining tree of the *Hygrobateslongiporus* complex obtained from 53 nucleotide COI sequences; 23 sequences were taken from Pešić et al. (2022a, 2023f), 26 sequences from Portugal are newly generated in this study, and four private sequences (HYDIR005-23, HYDIR006-23, HYDIR019-23, HYDIR020-23) from Turkey were directly taken from the BOLD. *Hygrobatesbalcanicus* from Portugal was used as outgroup. BINs are based on the barcode analysis from 16 May 2024. Country codes: FR = France, GR = Greece, IR = Iran, NO = Norway, PT = Portugal, RS = Serbia, TR = Turkey. Bootstrap values > 50% from 1000 bootstrap replicates on branches.

In this study, specimens keying to *H.longiporus* in Gerecke et al. (2016) have DNA barcodes that cluster in three unique BINs (BOLD:AFV9997, BOLD:AFW1423, BOLD:AFV9998) (Fig. 7). The *p*-distance to the nearest neigbour ranged between 11.38–13.62%, far exceeding the species thresholds (6.08% K2P distance) obtained in the study on the *H.longiporus* complex by Pešić et al. (2022a).

The identity of *H.longiporus* was recently questioned by Pešić et al. (2021b, 2022a) who found that DNA barcodes of the specimens assigned to the latter species in Europe and Turkey cluster within four distinct genetic lineages. The first cluster (BOLD:AEB8359) comprises a large number of *longiporus*-like specimens from different parts of Europe, including Norway, from where the species was originally described (Thor 1898). The second cluster (BOLD:AEM7299) is restricted to SE Europe and E Turkey, while the third cluster (BOLD:AEO0825) is known only from Corsica. Most likely the latter two clusters represent a cryptic species new to science. The fourth clade, known from northern Iran and eastern Turkey, recently was described by Pešić et al. (2022a) as *H.thori* Pešić & Smit, 2022.

As emphasized by Pešić et al. (2022a), a larger-scale study of *H.longiporus* complex is needed to establish a stable taxonomy for this group. The true identity of *H.longiporus* should be morphologically redefined with material from Norway. Morphological analysis revealed that in regard to the shape of palp and genital field, Portuguese specimens match the description of *H.falcilaminatus*, a species originally described from Azrou, Morocco on the basis of a single female (Walter 1926) and later reported from a wide area in the western Mediterranean (Spain and France). The species was synonymized with *H.longiporus* by Lundblad (1956) (see also Gerecke 1991 for a discussion about this species). Further research, including a redescription of *H.falcilaminatus*, is needed to clarify taxonomic status of the Portuguese lineages and relationship with other members of *H.longiporus* complex.

#### ﻿Family Unionicolidae Oudemans, 1909

##### ﻿Genus Neumania

Taxon classificationAnimaliaHydrachnidiaUnionicolidae

Lebert, 1879

4F77AE79-785B-596D-B69A-9FF73B7C3871

###### Note.

Three species so far reported from Portugal.

##### ﻿ Neumania (Neumania) elliptica

Taxon classificationAnimaliaHydrachnidiaUnionicolidae

Walter, 1925

CA9CC06C-8ADC-5B23-8014-52CE48905F6B

###### Material examined.

Portugal, **Guarda**: • Manteigas, Poço do Inferno, 40.373°N, 7.516°W, 1078 m a.s.l., 21 Aug. 2023, leg. Ferreira, Benitez-Bosco & Padilha, 1♂ (sequenced); • Seia, Rio Alva, Nossa Senhora do Desterro, river, 40.395°N, 7.694°W, 791 m a.s.l., 23 Aug. 2023, leg. Ferreira, Benitez-Bosco & Padilha, 1♀ (sequenced).

###### Remarks.

The specimens from Portugal morphologically match the description of *N.elliptica*. This species was originally described from Algeria (Walter 1925) and later on reported by Pešić et al. (2007) from Corsica (France) and Italy (Sardinia and Sicily). *Neumaniaelliptica* is a characteristic colonizer of pools in summer-warm lowland streams with silty substrate (Pešić et al. 2007).

The sequenced specimens from Portugal form a unique BIN (BOLD:AFU2122), with the nearest neighboring BIN being BOLD:ACR9513 (*p*-distance 19.16%), which includes specimens of *N.deltoides* from the Netherlands, Macedonia and Turkey, available in BOLD.

###### Distribution.

SW-Mediterranean. New for Portugal.

##### ﻿ Neumania (Neumania) imitata

Taxon classificationAnimaliaHydrachnidiaUnionicolidae

Koenike, 1908

BBB5777D-24A1-510A-BB9B-290120C20250

###### Material examined.

Portugal, **Porto** • Lousada, Parque Molinológico e Florestal de Pias, 41.268°N, 8.256°W, 170 m a.s.l., 1 Sep. 2023, leg. Ferreira, Sousa, Cruz-Oliveira & Girão, 1♂ (sequenced).

###### Remarks.

The examined male in our study keyed to *Neumaniaimitata* following Gerecke et al. (2016) and forms a unique BIN (BOLD:AFV0268). The *p*-distance between this BIN and its nearest neighbour, BOLD:ADF7924, which includes specimens of *N.imitata* from the Netherlands, was estimated at 8.65%, indicating the need for taxonomic revision of this species.

###### Distribution.

Europe; rare, reported from France, Italy, Germany, the Netherlands, Poland, and Montenegro. New for Portugal.

##### ﻿ Neumania (Neumania) limosa

Taxon classificationAnimaliaHydrachnidiaUnionicolidae

(Koch, 1836)

17D772B1-C314-5A0B-AE53-D0EFA7EF428B

###### Material examined.

Portugal, **Beja** • Mértola, Herdade de Alagães, pond, 37.673°N, 7.848°W, 18 May 2023, leg. Ferreira, Benitez-Bosco, Ekrem, Stur & Turaccio, 4♀ (sequenced); **Guarda**: • Manteigas, Serra de Baixo, Lagoa, 40.35°N, 7.549°W, 1431 m a.s.l., 21 Aug. 2023, leg. Ferreira, Benitez-Bosco & Padilha, 1♂, 3♀ (sequenced); • Gouveia, Ribeira do Covão do Urso, Barragem do Lagoacho, 40.385°N, 7.618°W, 1438 m a.s.l., 22 Aug. 2023, leg. Ferreira, Benitez-Bosco & Padilha, 1♂ (juv.) (sequenced).

###### Remarks.

The specimens from Portugal cluster within BOLD:ACS0551, which includes specimens of *N.limosa* from the Netherlands. The *p*-distance between this BIN and its nearest neighbor BOLD:AEF5902, which includes specimens from Montenegro assigned to *N.limosa*, was estimated at 3.21%.

###### Distribution.

Palaearctic. New for Portugal.

##### ﻿ Neumania (Neumania) uncinata

Taxon classificationAnimaliaHydrachnidiaUnionicolidae

Walter, 1927

ACDFCD3B-C2E8-57B2-AF20-57A057B48BE3

###### Material examined.

Portugal, **Faro** • Monchique, Caldas de Monchique, 37.287°N, 8.554°W, 23 May 2023, leg. Ferreira & Turaccio, 1♀ (sequenced). **Porto** • Vila do Conde, Rio Este, 41.378°N, 8.695°W, 15 m a.s.l., 7 Sep. 2023, leg. Ferreira, Cruz-Oliveira & Girão, 1♀ (sequenced).

###### Remarks.

The sequenced specimens from Portugal keyed to *N.uncinata* following Gerecke et al. (2016), and cluster within two unique BINs (BOLD:AFV0253, BOLD:AFV0269). The *p*-distance between Portuguese BINs and their closest neighbor, BOLD:AER9267, which includes specimens of *N.uncinata* from Sardinia (see Pešić and Goldschmidt 2023), was estimated at 8.78% and 13.14%, respectively, indicating the need for taxonomic revision of *N.uncinata* complex from a wider geographical area.

###### Distribution.

Western Palaearctic. In Portugal known from Estremadura (Lundblad 1956). *Neumaniaatlantida* Lundblad, 1962, originally described from Madeira by Lundblad (1962), was synonymized by Pešić et al. (2007) with *N.uncinata*.

##### ﻿ Neumania (Soarella) papillosa

Taxon classificationAnimaliaHydrachnidiaUnionicolidae

(Soar, 1902)

97B76798-4A9A-5F97-B073-4BC50EC68AE2

###### Material examined.

Portugal, **Beja** • Mértola, Corte do Pinto, 37.682°N, 7.512°W, 19 May 2023, leg. Ferreira, Benitez-Bosco, Ekrem, Stur & Turaccio, 1♂, 2♀ (sequenced). **Guarda** • Seia, Rio Alva, Praia Fluvial de Sabugueiro, river, 40.401°N, 7.64°W, 1021 m a.s.l., 24 Aug. 2023, leg. Ferreira, Benitez-Bosco & Padilha, 1♀ (sequenced).

###### Remarks.

The Portuguese specimens match the description of *N.papillosa*, forming a unique BIN (BOLD:AFO2116), with the nearest neighboring BIN being BOLD:ADS6560, which consists of 31 specimens of and unidentified *Neumania* sp. from South Africa. The *p*-distance between these two BINs was estimated at 14.26%.

###### Distribution.

Europe. In Portugal previously reported from Côa River near Santa Comba Dão (Beira Alta; Lundblad 1956).

##### ﻿Genus Unionicola

Taxon classificationAnimaliaHydrachnidiaUnionicolidae

Haldeman, 1842

52C3AEF8-0A93-5F80-B243-56262A4880B4

###### Note.

Three species so far reported from Portugal.

##### ﻿ Unionicola (Hexatax) minor

Taxon classificationAnimaliaHydrachnidiaUnionicolidae

(Soar, 1900)

DEA57F1D-1E51-56DA-9830-020EBA476700

###### Material examined.

Portugal, **Beja**: • Mértola, São João dos Caldeireiros, stream, 37.626°N, 7.81°W, 17 May 2023, leg. Ferreira, Benitez-Bosco, Ekrem, Stur & Turaccio, 1♀ (one palp dissected and slide mounted, RMNH); • Mértola, Moinho de Alferes 2, 37.503°N, 7.687°W, 19 May 2023, leg. Ferreira, Benitez-Bosco, Ekrem, Stur & Turaccio, 1♀ (sequenced).

###### Remarks.

Specimens keying to *U.minor* in Gerecke et al. (2016) have DNA barcodes that cluster in unique BIN (BOLD:AFO2171).

###### Distribution.

Widespread in Europe, here reported for the first time for Portugal.

#### ﻿Family Pionidae Thor, 1900

**Subfamily Foreliinae Thor, 1923**

##### ﻿Genus Forelia

Taxon classificationAnimaliaHydrachnidiaPionidae

Haller, 1882

D2DCC453-D626-58AA-8A82-0C8A788AA6BA

###### Note.

Only one species reported from Portugal.

##### ﻿ Forelia longipalpis

Taxon classificationAnimaliaHydrachnidiaPionidae

Maglio, 1924

CD97C38F-6914-550C-82C5-D6EA3DE0F38E

###### Material examined.

Portugal, **Guarda**: • Gouveia, Ribeira da Fervença, Barragem do Vale do Rossim, 40.4°N, 7.589°W, 1418 m a.s.l., 22 Aug. 2023 leg. Ferreira, Benitez-Bosco, Padilha, Andrade & Stur, 3♀, 1 deutonymph (sequenced); • Seia, Rio Alva, Nossa Senhora do Desterro, river, 40.395°N, 7.694°W, 791 m a.s.l., 23 Aug. 2023, leg. Ferreira, Benitez-Bosco & Padilha, 1♀ (sequenced); • Manteigas, Mondego, Covão da Ponte, 40.443°N, 7.514°W, 999 m a.s.l., 24 Aug. 2023, leg. Ferreira, Benitez-Bosco & Padilha, 1♀ (sequenced).

###### Remarks.

All barcoded specimens in our study were females. The shape of the genital field of specimens from Portugal morphologically match the description of *F.longipalpis* following Gerecke et al. (2016). The sequenced specimens cluster into two unique BINs (BOLD:AFX2876, BOLD:AFV3893), indicating the need for further taxonomic revision of this species. However, this revision should be postponed until males are available.

###### Distribution.

Widespread in Europe; new record for Portugal.

##### ﻿ Forelia variegator

Taxon classificationAnimaliaHydrachnidiaPionidae

(Koch, 1837)

0883E172-763A-5EC5-88D1-1EB976342552

###### Material examined.

Portugal, **Beja** • Mértola, São João dos Caldeireiros, stream, 37.625°N, 7.81°W, 17 May 2023, leg. Ferreira, Benitez-Bosco, Ekrem, Stur & Turaccio, 1♀ (sequenced). **Porto**: • Lousada, Parque Molinológico e Florestal de Pias, 41.268°N, 8.256°W, 170 m a.s.l., 1 Sep. 2023, leg. Ferreira, Sousa, Cruz-Oliveira & Girão, 2♀ (sequenced); • Vila do Conde, Rio Este, 41.378°N, 8.695°W, 15 m a.s.l., 7 Sep. 2023, leg. Ferreira, Sousa, Cruz-Oliveira & Girão, 1♂ (sequenced). **Guarda** • Rio Alva, Nossa Senhora do Desterro, river, 40.395°N, 7.694°W, 791 m a.s.l., 23 Aug. 2023, leg. Ferreira, Benitez-Bosco & Padilha, 1♀ (sequenced); • Seia, Rio Alva, Central hidroelétrica de Ponte dos Jugais, river, 40.385°N, 7.706°W, 555 m a.s.l., 23 Aug. 2023, leg. Ferreira, Benitez-Bosco & Padilha, 1♀ (sequenced).

###### Remarks.

Regarding the shape of the genital field, the specimens from Portugal morphologically match the description of *F.variegator* following Gerecke et al. (2016). The sequenced specimens from Portugal form a unique BIN (BOLD:AFU5459), with the nearest neighboring BIN being BOLD:ACS0537, which includes specimens of *F.variegator* from the Netherlands, Norway, North Macedonia, and Russia. The *p*-distance between these two BINs is estimated at 12.82%.

###### Distribution.

Palaearctic. In Portugal previously reported from Beira Alta and Estremadura (Lundblad 1956).

#### ﻿Subfamily Hydrochoreutinae K. Viets, 1942

##### ﻿Genus Hydrochoreutes

Taxon classificationAnimaliaHydrachnidiaPionidae

Koch, 1837

1D9E4B1C-B0CA-5882-AE2E-B42CD139218D

###### Note.

New genus for Portugal.

##### ﻿ Hydrochoreutes krameri

Taxon classificationAnimaliaHydrachnidiaPionidae

Piersig, 1896

31F87280-A6FF-560F-8090-90BB466D1701

###### Material examined.

Portugal, **Beja** • Mértola, Herdade de Alagães, pond, 37.673°N, 7.848°W, 18 May 2023, leg. Ferreira, Benitez-Bosco, Ekrem, Stur & Turaccio, 1♀ (sequenced). **Guarda**: • Seia, Covão do Forno, 40.369°N, 7.638°W, 1574 m a.s.l., 19 Aug. 2023, leg. Ferreira, Benitez-Bosco & Padilha, 1♂ (sequenced).

###### Remarks.

The sequenced specimens from Portugal cluster within BOLD:ACR9737. In addition to the specimen from Portugal, the BIN includes specimens of *Hydrochoreuteskrameri* from Norway, the Netherlands, and North Macedonia, available in BOLD. The *p*-distance between the latter BIN and its nearest neighbor, BOLD:ADZ1025, which includes specimens of *H.ungulates*, is estimated at 15.22%.

###### Distribution.

Palaearctic. New for Portugal.

#### ﻿Subfamily Pioninae Thor, 1900

##### ﻿Genus Nautarachna

Taxon classificationAnimaliaHydrachnidiaPionidae

Moniez, 1888

78233157-F2CD-571C-9DC1-67F500145A04

###### Note.

New genus for Portugal.

##### ﻿ Nautarachna crassa

Taxon classificationAnimaliaHydrachnidiaPionidae

(Koenike, 1908)

10F174D9-ACA6-5FB5-9FE0-44592D1E79D4

###### Material examined.

Portugal, **Guarda** • Manteigas, Casa do Cantoneiro, 40.418°N, 7.603°W, 1378 m a.s.l., 24 Aug. 2023, leg. Ferreira, Benitez-Bosco & Padilha, 1♀ (sequenced).

###### Remarks.

The sequenced female of *N.crassa* from Portugal forms a distinct BIN (BOLD:AFV0462). The *p*-distance between the specimen from Portugal and the barcode of a *N.crassa* specimen (MMHYD270-20) collected in Norway, is estimated at 16.7%, indicating the need for a taxonomic revision of *N.crassa* complex to identify possible undescribed cryptic species.

###### Distribution.

Europe; widespread but here reported for the first time for the water mite fauna of Portugal.

##### ﻿Genus Piona

Taxon classificationAnimaliaHydrachnidiaPionidae

Koch, 1842

43A109DA-B301-5812-9975-CCD3C6D43846

###### Note.

So far, two species of the genus have been reported from Portugal (Pešić et al. 2023b).

##### ﻿ Piona carnea

Taxon classificationAnimaliaHydrachnidiaPionidae

(Müller, 1776)

6605F964-646F-5177-84A9-31743A5D54A1

###### Material examined.

Portugal, **Beja**: • Mértola, São Sebastião dos Carros, 37.598°N, 7.754°W, 21 May 2023, leg. Ferreira, Benitez-Bosco, Ekrem, Stur & Turaccio, 2♂, 2♀, 3 deutonymphs (sequenced); • Mértola, Herdade de Alagães, dry stream site 2, 37.678°N, 7.848°W, 18 May 2023, leg. Ferreira, Benitez-Bosco, Ekrem, Stur & Turaccio, 1 deutonymph (sequenced). **Vila Real** • Murça, Noura stream, 41.409°N, 7.417°W, 421 m a.s.l., 12 Jul. 2023 leg. Ferreira S, Padilha 1 deutonymph (sequenced). **Guarda**: • Manteigas, Serra de Baixo, Lagoa, 40.35°N, 7.549°W, 1431 m a.s.l., 21 Aug. 2023, leg. Ferreira, Benitez-Bosco & Padilha, 2 deutonymphs (sequenced).

###### Remarks.

The specimens from Portugal morphologically match the description of *Pionacarnea*. These specimens cluster within two BINs: BOLD:ACM0527, which includes three unidentified specimens from Canada and three specimens from the Netherlands assigned to *P.carnea*, and BOLD:ACS0622, which includes specimens of *P.carnea* from Norway, Finland, the Netherlands, and Germany.

###### Distribution.

Holarctic. New record for Portugal.

##### ﻿ Piona variabilis

Taxon classificationAnimaliaHydrachnidiaPionidae

(Koch, 1836)

7D798821-4174-51F6-A452-729A8E09D4FC

###### Material examined.

Portugal, **Beja** • Mértola, São Sebastião dos Carros, 37.598°N, 7.754°W, 21 May 2023, leg. Ferreira, Benitez-Bosco, Ekrem, Stur & Turaccio, 1 deutonymph (sequenced; Table 1).

###### Remarks.

The single deutonymph from Portugal cluster within BOLD:AAU0701, which includes 11 specimens from Sweden, Norway, and the Netherlands assigned to *P.variabilis*, available in the BOLD database.

###### Distribution.

Europe. New record for Portugal.

#### ﻿Subfamily Tiphyinae Oudemans, 1941

##### ﻿Genus Pionopsis

Taxon classificationAnimaliaHydrachnidiaPionidae

Piersig, 1894

41A51D00-DE73-56E9-A22E-F8C7D559B4EE

###### Note.

One species of the genus reported from Portugal

##### ﻿ Pionopsis lutescens

Taxon classificationAnimaliaHydrachnidiaPionidae

(Hermann, 1804)

C741DEC2-727E-5121-9A7A-86C7256AB5FB

###### Material examined.

Portugal, **Porto** • Lousada, Parque Torre de Vilar, 41.287°N, 8.21°W, 274 m a.s.l., 1 Sep. 2023, leg. Ferreira, Sousa & Girão 1♀ (sequenced). **Guarda** • Seia, Cise, 40.419°N, 7.709°W, fountain, 505 m a.s.l., 25 Aug. 2023, leg. Ferreira, Benitez-Bosco & Padilha, 1♂, 1♀, 1 deutonymph (sequenced).

###### Remarks.

The examined male keyed to *Pionopsislutescens* following Gerecke et al. (2016). The Portuguese specimens form a unique BIN (BOLD:AFV3897). The *p*-distance between the latter BIN and its nearest neighbor, BOLD:AET1848, which includes specimens of *P.lutescens* from Montenegro, was estimated at 12.34%, indicating the need for taxonomic revision of *P.lutescens* complex to identify possible undescribed cryptic species.

###### Distribution.

Holarctic. In Portugal previously reported from Sintra-Monserrate Park and Palace, Estremadura (Lundblad 1956).

##### ﻿Genus Tiphys

Taxon classificationAnimaliaHydrachnidiaPionidae

Koch, 1836

0D1DD487-40E1-549C-9BA0-6F54749968CA

###### Note.

Only one species reported from Portugal.

##### ﻿ Tiphys torris

Taxon classificationAnimaliaHydrachnidiaPionidae

(Müller, 1776)

1FDFC7D0-90C8-529E-9159-1AACA93BAE4F

###### Material examined.

Portugal, **Beja** • Odemira, Ribeira de Seixe, Zambujeira do Mar, 37.399°N, 8.723°W, 45 m a.s.l., 23 May 2023, leg. Ekrem & Benitez-Bosco, 1♀ (sequenced). Guarda • Seia, Rio Alva, Nossa Senhora do Desterro, river, 40.395°N, 7.694°W, 791 m a.s.l., 23 Aug. 2023, leg. Ferreira, Benitez-Bosco & Padilha, 1♂ (sequenced); • Seia, Rio Alva, Praia Fluvial de Sabugueiro, river, 40.401°N, 7.64°W, 1021 m a.s.l., 24 Aug. 2023, leg. Ferreira, Benitez-Bosco & Padilha, 1♀ (sequenced).

###### Remarks.

The sequenced specimens from Portugal form a unique BIN (BOLD:AFP3352) with the nearest neighboring BIN being BOLD:ACR9977 (*p*-distance 1.92%), which includes one specimen from the Netherlands assigned to *T.torris* and three specimens from Norway assigned to *T.lapponicus*.

###### Distribution.

Europe. In Portugal previously reported from Estremadura (Lundblad 1956).

#### ﻿Family Aturidae Thor, 1900

##### ﻿Genus Aturus

Taxon classificationAnimaliaHydrachnidiaAturidae

Kramer, 1875

FCD4CA81-6810-5756-8C1B-9E097CE4A4D2

###### Note.

New genus record for Portugal.

##### ﻿ Aturus scaber

Taxon classificationAnimaliaHydrachnidiaAturidae

Kramer, 1875

5029C01E-1A0D-547C-875F-4E48828E6153

###### Material examined.

Portugal, **Porto**: • Lousada, Moinho da Tapada, 41.263°N, 8.307°W, 176 m a.s.l., 1 Sep. 2023, Ferreira, Sousa, Cruz-Oliveira & Girão, 1♂ (sequenced).

###### Remarks.

The specimen from Portugal clusters within BOLD:ACQ9097, which includes > 80 specimens of *A.scaber* from Norway and Germany in BOLD.

###### Distribution.

Western Palaearctic. New record for Portugal.

#### ﻿Family Mideopsidae Koenike, 1910

##### ﻿Genus Mideopsis

Taxon classificationAnimaliaHydrachnidiaMideopsidae

Koenike, 1910

DB69CC2A-BCEB-58C3-9665-0BAC57053EBF

###### Note.

Family and genus both new for Portugal.

##### ﻿ Mideopsis roztoczensis

Taxon classificationAnimaliaHydrachnidiaMideopsidae

Biesiadka & Kowalik, 1979

AE5CB231-F98D-5E26-8E1A-E4FDB632EDC6

Fig. 8

###### Material examined.

Portugal, **Beja**: • Mértola, Moinho de Alferes 1, 37.502°N, 7.69°W, 19 May 2023, leg. Ferreira, Benitez-Bosco, Ekrem, Stur & Turaccio, 1♀ (sequenced); Mértola, Pulo do Lobo, 37.805°N, 7.633°W, 18 May 2023, leg. Ferreira, Benitez-Bosco, Ekrem, Stur & Turaccio, 5♀, 1 deutonymph (sequenced); • Odemira, Ribeira de Seixe, Zambujeira do Mar, 37.398°N, 8.68°W, 75 m a.s.l., 23 May 2023, leg. Ekrem & Benitez-Bosco, 1♂ (sequenced). **Bragança** • Vinhais, Gasparona, 41.85°N, 7.013°W, 683 m a.s.l., 6 Jul. 2023, leg. Ferreira & Padilha, 1♀, 1 deutonymph (sequenced). **Guarda**: • Seia, Casa do Loureiro, 40.433°N, 7.701°W, 415 m a.s.l., 19 Jul. 2023 leg. Ferreira & Padilha, 1♂, 3♀, 1 deutonymph (sequenced); • Manteigas, Zêzere, Covão da Ametade, 40.328°N, 7.587°W, 1431 m a.s.l., 21 Aug. 2023, leg. Ferreira, Benitez-Bosco & Padilha, 1♂, 1♀, 1 deutonymph (sequenced); • Manteigas, Zêzere, Ponte dos Frades, 40.403°N, 7.526°W, 672 m a.s.l., 22 Aug. 2023, leg. Ferreira, Benitez-Bosco, Padilha, Andrade & Stur 1♀ (sequenced); • Manteigas, Poço do Inferno, 40.373°N, 7.516°W, 1078 m a.s.l., 21 Aug. 2023, leg. Ferreira, Benitez-Bosco & Padilha, 2♂, 1♀ (juv.), 1 deutonymph, 1♂ dissected and slide mounted (RMNH); • Gouveia, Rio Mondego, Casais de Folgosinho, 40.454°N, 7.493°W, 976 m a.s.l., 24 Aug. 2023, leg. Ferreira, Benitez-Bosco & Padilha, 1♂, 1♀ (sequenced); • Manteigas, Poio do Leão, 40.399°N, 7.541°W, 734 m a.s.l., 22 Aug. 2023, leg. Ferreira, Benitez-Bosco & Padilha, 2♂ (sequenced); • Seia, Rio Alva, Central hidroelétrica de Ponte dos Jugais, river, 40.385°N, 7.706°W, 555 m a.s.l., 23 Aug. 2023, leg. Ferreira, Benitez-Bosco & Padilha, 1♂, 2♀, 2 deutonymph (sequenced), 1♂ dissected and slide mounted (RMNH); • Rio Alva, Nossa Senhora do Desterro, river, 40.395°N, 7.694°W, 791 m a.s.l., 23 Aug. 2023, leg. Ferreira, Benitez-Bosco & Padilha, 2♀ (sequenced); • Manteigas, Mondego, Covão da ponte, 40.443°N, 7.514°W, 999 m a.s.l., 24 Aug. 2023, leg. Ferreira, Benitez-Bosco & Padilha, 1♂ (sequenced); • Seia, Rio Alva, Praia Fluvial de Sabugueiro, river, 40.401°N, 7.64°W, 1021 m a.s.l., 24 Aug. 2023, leg. Ferreira, Benitez-Bosco & Padilha, 2♀ (sequenced); • Manteigas, Casa do Cantoneiro, 40.418°N, 7.603°W, 1378 m a.s.l., 24 Aug. 2023, leg. Ferreira, Benitez-Bosco & Padilha, 2♂, 1♀ (sequenced). **Vila Real** • Murça, Noura stream, 41.409°N, 7.417°W, 421 m a.s.l., 12 Jul. 2023 leg. Ferreira S, Padilha 1♀ (sequenced).

###### Remarks.

**Figure 8.**
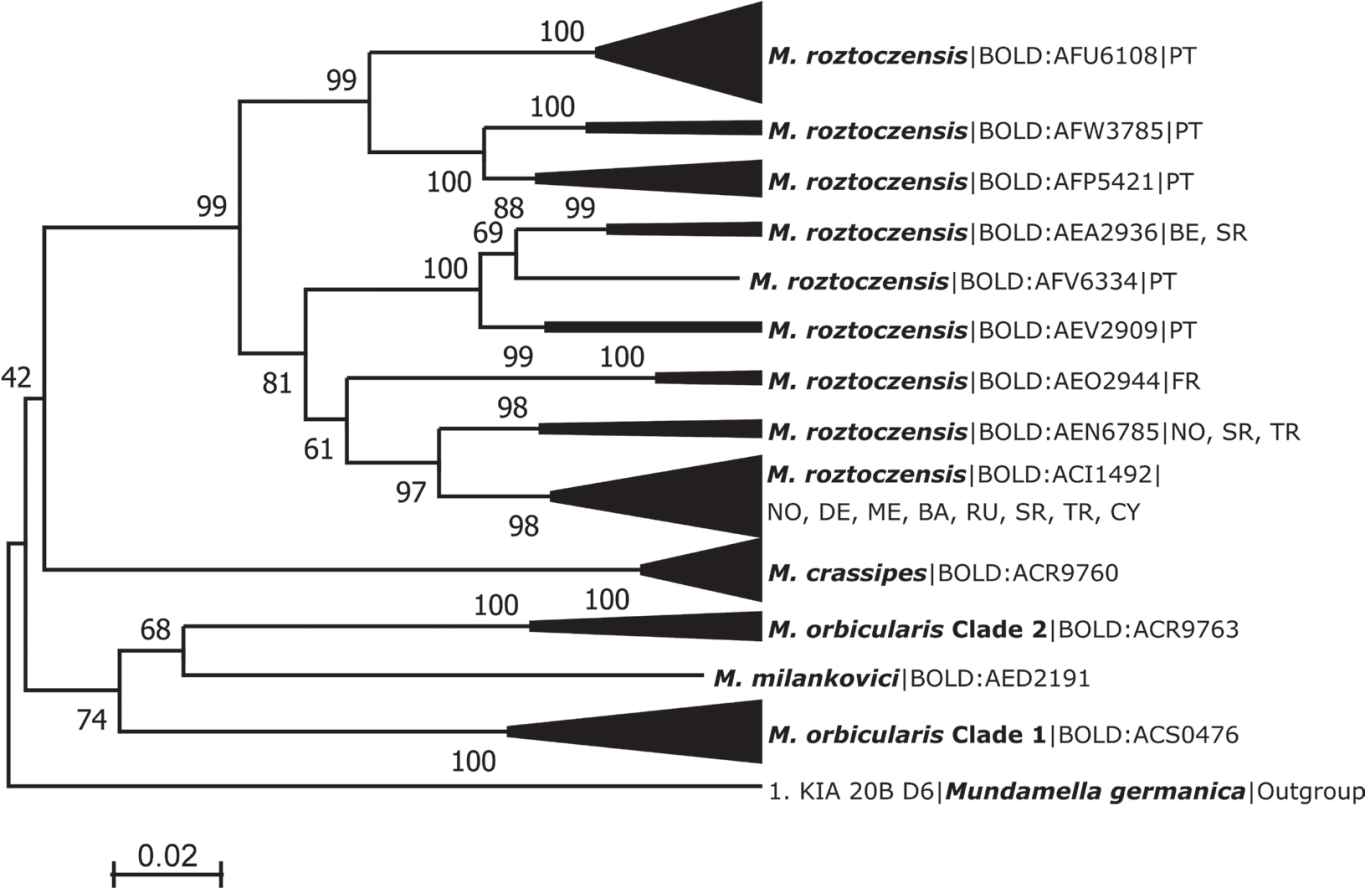
Neighbor-Joining tree of the genus *Mideopsis* obtained from 112 nucleotide COI sequences; 71 sequences were taken from Pešić et al. (2023a) and 41 sequences from Portugal are newly generated in this study. *Mundamellagermanica* from Montenegro was used as outgroup. BINs are based on the barcode analysis from 16 May 2024. Country codes: BA = Bosnia and Herzegovina, BE = Belgium, CY = Cyprus, DE = Germany, FR = France, ME = Montenegro, PT = Portugal, RS = Serbia, RU = Russia, TR = Turkey. Bootstrap values > 50% from 1000 bootstrap replicates on branches.

The specimens examined in our study match the description of *Mideopsisroztoczensis*, when following Biesiadka and Kowalik (1979). *Mideopsisroztoczensis* is characterized by a more elevated dorsal shield with distinctly visible anteriorly diverging lines of particularly faint fine porosity, and by the the shape of the male ejaculatory complex with the wedge-shaped anterior ramus being wider and with a characteristic arrow-shaped delimited area. Recently, Pešić et al. (2023e) showed that *M.roztoczensis* is a genetically variable species, comprising four BINs (BOLD:ACI1492, BOLD:AEN6785, BOLD:AEA2936, BOLD:AEO2944) widely distributed in Europe. In this study, we detected an additional five unique BINs (BOLD:AFU6108, BOLD:AFP5421, BOLD:AFW3785, BOLD:AFV6334, BOLD:AEV2909) within the Portuguese *M.roztoczensis*-like specimens (Fig. 8), all of them unique, and some of them present at the same sites (e.g., BOLD:AFU6108 and BOLD:AFW3785 in Casa do Loureiro, BOLD:AFU6108 and BOLD:AFV6334 in Casa do Cantoneiro).

###### Distribution.

Europe, Turkey. New record for Portugal.

#### ﻿Family Momoniidae K. Viets, 1926

##### ﻿Genus Momonia

Taxon classificationAnimaliaHydrachnidiaMomoniidae

Halbert, 1906

2491AE7A-DB1F-5209-B5C7-3F622E19EE35

###### Note.

Genus and family both new for Portugal.

##### ﻿ Momonia (Momonia) falcipalpis

Taxon classificationAnimaliaHydrachnidiaMomoniidae

Halbert, 1906

E406273A-205F-5980-9DD6-32B5636B0189

###### Material examined.

Portugal, **Guarda** • Manteigas, Poço do Inferno, 40.373°N, 7.516°W, 1078 m a.s.l., 21 Aug. 2023, leg. Ferreira, Benitez-Bosco & Padilha, 1♀ (sequenced).

###### Remarks.

The examined specimen in our study keyed to *Momoniafalcipalpis* and forms a unique BIN (BOLD:AFX3396).

###### Distribution.

Europe; rare, known from Ireland, France, Italy, and Russia. New record for Portugal.

#### ﻿Family Arrenuridae Thor, 1900

##### ﻿Genus Arrenurus

Taxon classificationAnimaliaHydrachnidiaMomoniidae

Dugès, 1834

C59B26C4-90F9-5EA5-9D92-FEBDDB4C61A2

###### Note.

Seven species are reported from Portugal; one of them (*Arrenurusautochthonus* Lundblad, 1941) is endemic to Madeira.

##### ﻿ Arrenurus (Arrenurus) albator

Taxon classificationAnimaliaHydrachnidiaMomoniidae

(Müller, 1776)

3CB9C6D8-58F6-5FF1-991E-AEEF1D04534B

###### Material examined.

Portugal, **Guarda**: • Gouveia, Ribeira da Fervença, Barragem do Vale do Rossim, 40.4°N, 7.589°W, 1418 m a.s.l., 22 Aug. 2023 leg. Ferreira, Benitez-Bosco, Padilha, Andrade & Stur, 2♂, 2♀ (sequenced); • Gouveia, Ribeira do Covão do Urso, Barragem do Lagoacho, 40.385°N, 7.618°W, 1438 m a.s.l., 22 Aug. 2023 leg. Ferreira, Benitez-Bosco, Padilha, Andrade & Stur, 1♀ (sequenced).

###### Remarks.

The examined specimens from Portugal cluster within BOLD:ACR9639, which includes one specimen of *Arrenurusalbator* from the Netherlands, available in the BOLD database.

###### Distribution.

Western Palaearctic. In Portugal previously reported from Beira Alta (Lundblad 1956).

##### ﻿ Arrenurus (Arrenurus) szalayi

Taxon classificationAnimaliaHydrachnidiaMomoniidae

Lundblad, 1954

B480D81F-BCFF-5B27-8428-1B2873C26B20

###### Material examined.

Portugal, **Beja** • Mértola, Moinho de Alferes 1, 37.502°N, 7.69°W, 19 May 2023, leg. Ferreira, Benitez-Bosco, Ekrem, Stur & Turaccio, 2♂ incl. one juv., (both sequenced).

###### Remarks.

The examined males from Moinho de Alferes match the description of *A.szalayi*, a species originally described from Ribeira d’Odivelas in Portugal (Lundblad 1956). The sequenced specimens from Portugal clustered within one BIN (BOLD:ACS0403), which in addition to the specimens from Portugal, includes a number of specimens collected from a large geographic area, from Norway to South Africa and from the Netherlands to Kyrgyzstan, and that are morphologically assigned to *A.bicuspidator* Berlese, 1885, or to *A.radiatus* Piersig, 1894. From both species, *A.szalayi*, at least morphologically, can be easily separated by the characteristic shape of the male petiole (see Lundblad 1956: fig. 161A). Further research is needed to understand the factors behind this grouping and the implication of this lack of genetic differentiation between three morphological different species.

###### Distribution.

Portugal; previously reported from Ribeira de Odivelas, Alentejo (Lundblad 1954, 1956).

##### ﻿ Arrenurus (Arrenurus) leuckarti

Taxon classificationAnimaliaHydrachnidiaMomoniidae

Piersig, 1894

F04C6010-C37C-5BF2-B853-A700840F5AC8

###### Material examined.

Portugal, **Guarda**: • Manteigas, Poço do Inferno, 40.373°N, 7.516°W, 1078 m a.s.l., 21 Aug. 2023, leg. Ferreira, Benitez-Bosco & Padilha, 1♂ (sequenced); Manteigas, Casa do Cantoneiro, 40.418°N, 7.603°W, 1378 m a.s.l., 24 Aug. 2023, leg. Ferreira, Benitez-Bosco & Padilha, 1♀ (sequenced).

###### Remarks.

The examined specimens from Portugal cluster within BOLD:ACR9670, which includes specimens of *Arrenurusleuckarti* from the Netherlands.

###### Distribution.

Western, eastern, and central Europe. New record for Portugal.

##### ﻿ Arrenurus (Arrenurus) neumani

Taxon classificationAnimaliaHydrachnidiaMomoniidae

Piersig, 1895

0245FD41-6B87-5ACC-88A9-13BFFD2EFF70

###### Material examined.

Portugal. Beja • Mértola, Moinho de Alferes 1, 37.502°N, 7.69°W, 19 May 2023, leg. Ferreira, Benitez-Bosco, Ekrem, Stur & Turaccio, 3♂, 1♀ (sequenced).

###### Remarks.

The sequenced specimens from Portugal form a unique BIN (BOLD:AFP6143) with the nearest neighboring BIN being BOLD:ACR9801, which includes specimens of *A.neumani*, from the Netherlands, Norway and Poland in BOLD. The *p*-distance between these two BINs was estimated at 5.9%.

###### Distribution.

Palaearctic. New record for Portugal.

##### ﻿ Arrenurus (Arrenurus) cf.tricuspidator

Taxon classificationAnimaliaHydrachnidiaMomoniidae

(Müller, 1776)

DDBD35F1-4726-5219-BD28-BC30A41A586A

###### Material examined.

Portugal, **Beja** • Mértola, Moinho de Alferes 1, 37.502°N, 7.69°W, 19 May 2023, leg. Ferreira, Benitez-Bosco, Ekrem, Stur & Turaccio, 1 deutonymph (sequenced).

###### Remarks.

One deutonymph from Portugal, used in this study for molecular analysis, forms a unique cluster BOLD:AFU3639, with a *p*-distance estimated at 3.21% to the closest neigbouring BIN, BOLD:ACS0825, which includes specimens of *A.tricuspidator* from the Netherlands, Norway, and Germany.

###### Distribution.

Palaearctic. New record for Portugal.

##### ﻿ Arrenurus (Megaluracarus) globator

Taxon classificationAnimaliaHydrachnidiaMomoniidae

(Müller, 1776)

177E68F7-7233-506E-BE1F-FD1EF162A6FB

###### Material examined.

Portugal: **Beja**: • Mértola, São Sebastião dos Carros, 37.598°N, 7.754°W, 21 May 2023, leg. Ferreira, Benitez-Bosco, Ekrem, Stur & Turaccio, 2♂, 1♀ (sequenced); • Mértola, Ribeira de Carreiras, São Miguel do Pinheiro, 37.552°N, 7.85°W, 21 May 2023, leg. Ferreira, Benitez-Bosco, Ekrem, Stur & Turaccio, 1♂, 2♀ (sequenced); • Moinho de Alferes 1, 37.502°N, 7.69°W, 19 May 2023, leg. Ferreira, Benitez-Bosco, Ekrem, Stur & Turaccio, 1♀ (sequenced).

###### Remarks.

The sequenced specimens from Portugal form a unique BIN (BOLD:AFO3503) with the nearest neighboring BIN being BOLD:ACS0765, which includes 34 specimens from the Netherlands, Norway, Poland and North Macedonia partially assigned to *A.globator* or *A.tubulator*. The *p*-distance between these two BINs was estimated at 5.45%.

###### Distribution.

Palaearctic. In Portugal previously reported from Alentejo (Lundblad 1956).

##### ﻿ Arrenurus (Megaluracarus) cf.zachariae

Taxon classificationAnimaliaHydrachnidiaMomoniidae

Koenike, 1886

B52E803F-A56C-5526-953E-5932167C291C

###### Material examined.

Portugal, Bragança • Vinhais, Gasparona, 41.85°N, 7.013°W, 693 m a.s.l., 6 Jul. 2023, leg. Ferreira & Padilha, 1 deutonymph (sequenced).

###### Remarks.

One deutonymph from Portugal forms a unique cluster BOLD:AFU0319, with a *p*-distance to the closest neighboring BIN, BOLD:ADF7386, which includes mostly specimens assigned to *A.zachariae*, estimated at 2.56%.

###### Distribution.

Europe. New record for Portugal.

## ﻿Discussion

Our study provides the first DNA barcode reference library for Portuguese water mites. Our findings confirm the presence of 29 of the 101 previously recorded species; however, it also added 36 new species for the water mite fauna of Portugal, two of which are described as new to science. We found that 47.4% of the Portuguese water mites having sequences collected in Portugal were represented in BOLD. Intraspecific distances for some species were high indicating the incongruence between morphology and DNA barcodes, and therefore the need for further taxonomic revision of these species to identify possible undescribed cryptic diversity. The identity of some species in the absence of available males, for example *Foreliavariegator* and *F.longipalpis*, remains questionable and needs further morphological evaluation.

Our research provided 75 BINs, 38 of which were new to BOLD. Seven species in our study were represented by multiple BINs: *Mideopsisroztoczensis* (five BINS), *Hygrobateslongiporus* (three BINS), *Lebertiainsignis*, *Atractidesinflatus*, *Neumaniauncinata*, *Forelialongipalpis*, and *Pionacarnea*, each with two BINs. Furthermore, examining molecular diversity at a continental scale, we found more such cases. For example two BINs were detected within *Limnocharesaquatica* (BOLD:AFV0270, BOLD:ACS0438), a species considered to be widely distributed in the Holarctic. Three BINs were also detected within *Neumaniauncinata* (BOLD:AFV0253, BOLD:AFV0269, BOLD:AER9267), a species widely distributed in the Mediterranean region, and *N.imitata* (BOLD:AFV0268, BOLD:ADF7924, BOLD:AED4073), a species that is less common, but with a wide distribution from Sweden to Portugal. A high genetic divergence (16.7% *p*-distance) was found between the specimen of *Nautarachnacrassa* from Portugal and a specimen of this species collected in Norway. Our study, based on the available data in BOLD suggests also that *Pionopsislutescens*, a species common in many types of standing water in the Holarctic, includes at least four BINs in Europe. In this study we retrieved one, BOLD:AFV3897, which includes only specimens from Portugal. Two others are present in the central and northern parts of Europe, BOLD:ACS0644 in the Netherlands and Norway and BOLD:ACR9955, so far found only in the Netherlands. Finally, the fourth BIN, BOLD:AET1848, is known from Montenegro. Further research is needed to understand the taxonomic implications of these genetic divergences.

Our results show the usefulness of using BINs to detect possible cryptic species and to investigate the distribution patterns of water mite species whose presence in certain geographical areas would be difficult to confirm without molecular evidence. In this study we confirmed the presence of *Hygrobatesfluviatilis* in Portugal, which represent the southernmost record of this widely distributed species. We also added the first record of *H.balcanicus*. The latter rhitrobiontic species, so far recorded from Serbia and Bulgaria, was probably previously confused with *H.fluviatilis*, but new findings from Portugal indicate that this species is widespread in the Mediterranean region.

Furthermore, our results demonstrate the efficiency of using DNA barcoding to identify preadult stages, particularly deutonymphs, whose identification to the species-level is often not possible without accompanying adult stages when using morphology. In this study, two individuals of *Arrenurus* deutonymhs were assigned to *A.tricuspidator* and *A.zachariae*, respectively, based on matching their DNA barcodes with the BINs of these species available in BOLD. Based on morphology alone, identification of these preadult stages would be difficult, if not impossible.

In summary, this study exemplifies the high molecular diversity of Portuguese water mites as well as the need to intensify international cooperation in the generation and curation of DNA barcode reference libraries.

## Supplementary Material

XML Treatment for Eylais

XML Treatment for Eylais tantilla

XML Treatment for Limnochares

XML Treatment for Limnochares aquatica

XML Treatment for Hydrodroma

XML Treatment for Hydrodroma despiciens

XML Treatment for Protzia

XML Treatment for Protzia annularis

XML Treatment for Lebertia

XML Treatment for Lebertia (Lebertia) fimbriata

XML Treatment for Lebertia (Lebertia) sparsicapillata

XML Treatment for Lebertia (Lebertia) variolata

XML Treatment for Lebertia (Pilolebertia) gibbosa

XML Treatment for Lebertia (Pilolebertia) algeriensis

XML Treatment for Lebertia (Pilolebertia) insignis

XML Treatment for Lebertia (Pilolebertia) porosa

XML Treatment for Lebertia (Pilolebertia) porosa

XML Treatment for Lebertia (Pilolebertia) pilosa

XML Treatment for Lebertia (Lebertia) pusilla

XML Treatment for Oxus

XML Treatment for Oxus (Oxus) cf.angustipositus

XML Treatment for Oxus (Oxus) lusitanicus

XML Treatment for Oxus (Oxus) musculus

XML Treatment for Oxus (Oxus) ovalis

XML Treatment for Oxus (Gnaphiscus) setosus

XML Treatment for Sperchon

XML Treatment for Sperchon (Hispidosperchon) algeriensis

XML Treatment for Sperchon (Hispidosperchon) clupeifer

XML Treatment for Sperchon (Hispidosperchon) compactilis

XML Treatment for Monatractides

XML Treatment for Monatractides (Monatractides) madritensis

XML Treatment for Monatractides (Monatractides) stadleri

XML Treatment for Monatractides (Monatractides)

XML Treatment for Torrenticola

XML Treatment for Torrenticola (Torrenticola) elliptica

XML Treatment for Torrenticola (Torrenticola) soniae

XML Treatment for Torrenticola (Torrenticola) elisabethae

XML Treatment for Torrenticola (Torrenticola) tenuipalpis

XML Treatment for Limnesia

XML Treatment for Limnesia (Limnesia) acuminata

XML Treatment for Limnesia (Limnesia) iberica

XML Treatment for Limnesia (Limnesia) koenikei

XML Treatment for Limnesia (Limnesia) maculata

XML Treatment for Limnesia (Limnesia) walteri

XML Treatment for Atractides

XML Treatment for Atractides (Atractides) inflatus

XML Treatment for Atractides (Atractides) marizae

XML Treatment for Atractides (Atractides) nodipalpis

XML Treatment for Atractides (Atractides) robustus

XML Treatment for Hygrobates

XML Treatment for Hygrobates balcanicus

XML Treatment for Hygrobates fluviatilis

XML Treatment for Hygrobates longiporus

XML Treatment for Neumania

XML Treatment for Neumania (Neumania) elliptica

XML Treatment for Neumania (Neumania) imitata

XML Treatment for Neumania (Neumania) limosa

XML Treatment for Neumania (Neumania) uncinata

XML Treatment for Neumania (Soarella) papillosa

XML Treatment for Unionicola

XML Treatment for Unionicola (Hexatax) minor

XML Treatment for Forelia

XML Treatment for Forelia longipalpis

XML Treatment for Forelia variegator

XML Treatment for Hydrochoreutes

XML Treatment for Hydrochoreutes krameri

XML Treatment for Nautarachna

XML Treatment for Nautarachna crassa

XML Treatment for Piona

XML Treatment for Piona carnea

XML Treatment for Piona variabilis

XML Treatment for Pionopsis

XML Treatment for Pionopsis lutescens

XML Treatment for Tiphys

XML Treatment for Tiphys torris

XML Treatment for Aturus

XML Treatment for Aturus scaber

XML Treatment for Mideopsis

XML Treatment for Mideopsis roztoczensis

XML Treatment for Momonia

XML Treatment for Momonia (Momonia) falcipalpis

XML Treatment for Arrenurus

XML Treatment for Arrenurus (Arrenurus) albator

XML Treatment for Arrenurus (Arrenurus) szalayi

XML Treatment for Arrenurus (Arrenurus) leuckarti

XML Treatment for Arrenurus (Arrenurus) neumani

XML Treatment for Arrenurus (Arrenurus) cf.tricuspidator

XML Treatment for Arrenurus (Megaluracarus) globator

XML Treatment for Arrenurus (Megaluracarus) cf.zachariae

## ﻿Additional information

### Author ORCIDs

Vladimir Pešić https://orcid.org/0000-0002-9724-345X

Andrzej Zawal https://orcid.org/0000-0002-5838-6060

Sónia Ferreira https://orcid.org/0000-0002-6884-3966

Laura Benitez-Bosco https://orcid.org/0000-0002-8863-8353

Dinis Girão https://orcid.org/0009-0008-4842-0721

Paolo Turaccio https://orcid.org/0009-0000-8627-6718

Samantha Rossini https://orcid.org/0009-0009-6881-2527

Giorgia Staffoni https://orcid.org/0009-0003-5916-1757

Torbjørn Ekrem https://orcid.org/0000-0003-3469-9211

### Data availability

All of the data that support the findings of this study are available in the main text or Supplementary Information.

## Supplementary materials

Supplementary material 1 BOLD TaxonID Tree Data type pdf Explanation note Compact Neighbor-Joining tree of all analyzed water mite species based on Kimura 2-parameter distances. BINs are based on the barcode analysis from 16 May 2024. The analyses involved all 307 COI nucleotide sequences. https://binary.pensoft.net/file/1164873This dataset is made available under the Open Database License (http://opendatacommons.org/licenses/odbl/1.0/). The Open Database License (ODbL) is a license agreement intended to allow users to freely share, modify, and use this Dataset while maintaining this same freedom for others, provided that the original source and author(s) are credited.Vladimir Pešić, Andrzej Zawal, Sónia Ferreira, Laura Benitez-Bosco, Ana Cruz-Oliveira, Dinis Girão, Adriana Padilha, Paolo Turaccio, Samantha Rossini, Lorenzo Ballini, Giorgia Staffoni, Sara Fratini, Claudio Ciofi, Alessio Iannucci, Torbjørn Ekrem, Elisabeth Stur

Supplementary material 2 List of *Torrenticola* specimens Data type doc Explanation note List of *Torrenticola* specimens used for building the Neighbor-Joining (NJ) tree (Fig. 6). Details on the specimens from Portugal are given in Table 1. GenBank numbers are indicated by *. BINs are based on the barcode analysis from 16 May 2024. Country codes (alpha-2 code): AT = Austria, BA = Bosnia and Herzegovina, CR = Croatia, DE = Germany, ES = Spain, FR = France, GR = Greece, IR = Iran, IT = Italy, NO = Norway, NL = the Netherlands, ME = Montenegro, MK = North Macedonia, PT = Portugal, RS = Serbia, TR = Turkey. https://binary.pensoft.net/file/1164874This dataset is made available under the Open Database License (http://opendatacommons.org/licenses/odbl/1.0/). The Open Database License (ODbL) is a license agreement intended to allow users to freely share, modify, and use this Dataset while maintaining this same freedom for others, provided that the original source and author(s) are credited.Vladimir Pešić, Andrzej Zawal, Sónia Ferreira, Laura Benitez-Bosco, Ana Cruz-Oliveira, Dinis Girão, Adriana Padilha, Paolo Turaccio, Samantha Rossini, Lorenzo Ballini, Giorgia Staffoni, Sara Fratini, Claudio Ciofi, Alessio Iannucci, Torbjørn Ekrem, Elisabeth Stur
