# Supplementary material for: ﻿DNA barcode library of Portuguese water mites, with the descriptions of two new species (Acari, Hydrachnidia)
Source: Zookeys. 2024 Oct 31;1217:119–71. doi: 10.3897/zookeys.1217.131730 (PMC11544308; doi:10.3897/zookeys.1217.131730)
Supplement: Supplementary material 1 — BOLD TaxonID Tree [file zookeys-1217-119_article-131730__-s001.pdf]

BOLD TaxonID Tree

Title	:	Tree Result - DS-PGHYD (307 records selected)
Date	:	16-May-2024
Data Type	:	Nucleotide
Distance Model	:	Kimura 2 Parameter
Marker	:	COI-5P
Colourization	:	[blue]=Stop Codons [red]=Contamination or misidentification

Label	:	Sample ID
Label	:	Process ID
Label	:	Taxonomy Notes
Label	:	Taxon
Label	:	Province/State
Label	:	Barcode Cluster (BIN)

Sequence Count	:	307
Species count	:	49
Genus count	:	24
Family count	:	16
Unidentified	:	55

BIN Count	:	76

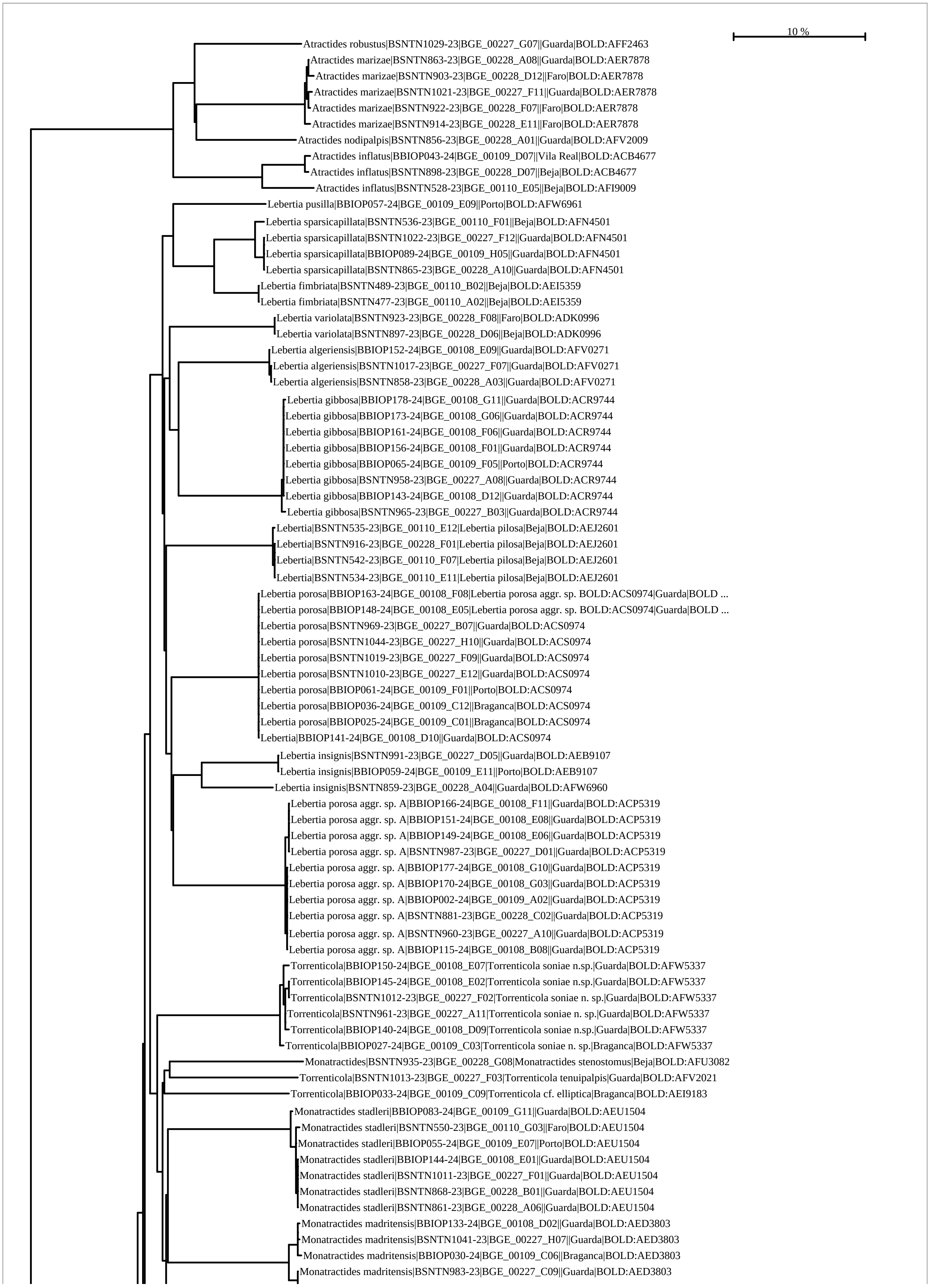
10 %
Atractides robustus|BSNTN1029-23|BGE_00227_G07||Guarda|BOLD:AFF2463
Atractides marizae|BSNTN863-23|BGE_00228_A08||Guarda|BOLD:AER7878
Atractides marizae|BSNTN903-23|BGE_00228_D12||Faro|BOLD:AER7878
Atractides marizae|BSNTN1021-23|BGE_00227_F11||Guarda|BOLD:AER7878
Atractides marizae|BSNTN922-23|BGE_00228_F07||Faro|BOLD:AER7878
Atractides marizae|BSNTN914-23|BGE_00228_E11||Faro|BOLD:AER7878
Atractides nodipalpis|BSNTN856-23|BGE_00228_A01||Guarda|BOLD:AFV2009
Atractides inflatus|BBIOP043-24|BGE_00109_D07||Vila Real|BOLD:ACB4677
Atractides inflatus|BSNTN898-23|BGE_00228_D07||Beja|BOLD:ACB4677
Atractides inflatus|BSNTN528-23|BGE_00110_E05||Beja|BOLD:AFI9009
Lebertia pusilla|BBIOP057-24|BGE_00109_E09||Porto|BOLD:AFW6961
Lebertia sparsicapillata|BSNTN536-23|BGE_00110_F01||Beja|BOLD:AFN4501
Lebertia sparsicapillata|BSNTN1022-23|BGE_00227_F12||Guarda|BOLD:AFN4501
Lebertia sparsicapillata|BBIOP089-24|BGE_00109_H05||Guarda|BOLD:AFN4501
Lebertia sparsicapillata|BSNTN865-23|BGE_00228_A10||Guarda|BOLD:AFN4501
Lebertia fimbriata|BSNTN489-23|BGE_00110_B02||Beja|BOLD:AEI5359
Lebertia fimbriata|BSNTN477-23|BGE_00110_A02||Beja|BOLD:AEI5359
Lebertia variolata|BSNTN923-23|BGE_00228_F08||Faro|BOLD:ADK0996
Lebertia variolata|BSNTN897-23|BGE_00228_D06||Beja|BOLD:ADK0996
Lebertia algeriensis|BBIOP152-24|BGE_00108_E09||Guarda|BOLD:AFV0271
Lebertia algeriensis|BSNTN1017-23|BGE_00227_F07||Guarda|BOLD:AFV0271
Lebertia algeriensis|BSNTN858-23|BGE_00228_A03||Guarda|BOLD:AFV0271
Lebertia gibbosa|BBIOP178-24|BGE_00108_G11||Guarda|BOLD:ACR9744
Lebertia gibbosa|BBIOP173-24|BGE_00108_G06||Guarda|BOLD:ACR9744
Lebertia gibbosa|BBIOP161-24|BGE_00108_F06||Guarda|BOLD:ACR9744
Lebertia gibbosa|BBIOP156-24|BGE_00108_F01||Guarda|BOLD:ACR9744
Lebertia gibbosa|BBIOP065-24|BGE_00109_F05||Porto|BOLD:ACR9744
Lebertia gibbosa|BSNTN958-23|BGE_00227_A08||Guarda|BOLD:ACR9744
Lebertia gibbosa|BBIOP143-24|BGE_00108_D12||Guarda|BOLD:ACR9744
Lebertia gibbosa|BSNTN965-23|BGE_00227_B03||Guarda|BOLD:ACR9744
Lebertia|BSNTN535-23|BGE_00110_E12|Lebertia pilosa|Beja|BOLD:AEJ2601
Lebertia|BSNTN916-23|BGE_00228_F01|Lebertia pilosa|Beja|BOLD:AEJ2601
Lebertia|BSNTN542-23|BGE_00110_F07|Lebertia pilosa|Beja|BOLD:AEJ2601
Lebertia|BSNTN534-23|BGE_00110_E11|Lebertia pilosa|Beja|BOLD:AEJ2601
Lebertia porosa|BBIOP163-24|BGE_00108_F08|Lebertia porosa aggr. sp. BOLD:ACS0974|Guarda|BOLD ...
Lebertia porosa|BBIOP148-24|BGE_00108_E05|Lebertia porosa aggr. sp. BOLD:ACS0974|Guarda|BOLD ...
Lebertia porosa|BSNTN969-23|BGE_00227_B07||Guarda|BOLD:ACS0974
Lebertia porosa|BSNTN1044-23|BGE_00227_H10||Guarda|BOLD:ACS0974
Lebertia porosa|BSNTN1019-23|BGE_00227_F09||Guarda|BOLD:ACS0974
Lebertia porosa|BSNTN1010-23|BGE_00227_E12||Guarda|BOLD:ACS0974
Lebertia porosa|BBIOP061-24|BGE_00109_F01||Porto|BOLD:ACS0974
Lebertia porosa|BBIOP036-24|BGE_00109_C12||Braganca|BOLD:ACS0974
Lebertia porosa|BBIOP025-24|BGE_00109_C01||Braganca|BOLD:ACS0974
Lebertia|BBIOP141-24|BGE_00108_D10||Guarda|BOLD:ACS0974
Lebertia insignis|BSNTN991-23|BGE_00227_D05||Guarda|BOLD:AEB9107
Lebertia insignis|BBIOP059-24|BGE_00109_E11||Porto|BOLD:AEB9107
Lebertia insignis|BSNTN859-23|BGE_00228_A04||Guarda|BOLD:AFW6960
Lebertia porosa aggr. sp. A|BBIOP166-24|BGE_00108_F11||Guarda|BOLD:ACP5319
Lebertia porosa aggr. sp. A|BBIOP151-24|BGE_00108_E08||Guarda|BOLD:ACP5319
Lebertia porosa aggr. sp. A|BBIOP149-24|BGE_00108_E06||Guarda|BOLD:ACP5319
Lebertia porosa aggr. sp. A|BSNTN987-23|BGE_00227_D01||Guarda|BOLD:ACP5319
Lebertia porosa aggr. sp. A|BBIOP177-24|BGE_00108_G10||Guarda|BOLD:ACP5319
Lebertia porosa aggr. sp. A|BBIOP170-24|BGE_00108_G03||Guarda|BOLD:ACP5319
Lebertia porosa aggr. sp. A|BBIOP002-24|BGE_00109_A02||Guarda|BOLD:ACP5319
Lebertia porosa aggr. sp. A|BSNTN881-23|BGE_00228_C02||Guarda|BOLD:ACP5319
Lebertia porosa aggr. sp. A|BSNTN960-23|BGE_00227_A10||Guarda|BOLD:ACP5319
Lebertia porosa aggr. sp. A|BBIOP115-24|BGE_00108_B08||Guarda|BOLD:ACP5319
Torrenticola|BBIOP150-24|BGE_00108_E07|Torrenticola soniae n.sp.|Guarda|BOLD:AFW5337
Torrenticola|BBIOP145-24|BGE_00108_E02|Torrenticola soniae n.sp.|Guarda|BOLD:AFW5337
Torrenticola|BSNTN1012-23|BGE_00227_F02|Torrenticola soniae n. sp.|Guarda|BOLD:AFW5337
Torrenticola|BSNTN961-23|BGE_00227_A11|Torrenticola soniae n. sp.|Guarda|BOLD:AFW5337
Torrenticola|BBIOP140-24|BGE_00108_D09|Torrenticola soniae n.sp.|Guarda|BOLD:AFW5337
Torrenticola|BBIOP027-24|BGE_00109_C03|Torrenticola soniae n. sp.|Braganca|BOLD:AFW5337
Monatractides|BSNTN935-23|BGE_00228_G08|Monatractides stenostomus|Beja|BOLD:AFU3082
Torrenticola|BSNTN1013-23|BGE_00227_F03|Torrenticola tenuipalpis|Guarda|BOLD:AFV2021
Torrenticola|BBIOP033-24|BGE_00109_C09|Torrenticola cf. elliptica|Braganca|BOLD:AEI9183
Monatractides stadleri|BBIOP083-24|BGE_00109_G11||Guarda|BOLD:AEU1504
Monatractides stadleri|BSNTN550-23|BGE_00110_G03||Faro|BOLD:AEU1504
Monatractides stadleri|BBIOP055-24|BGE_00109_E07||Porto|BOLD:AEU1504
Monatractides stadleri|BBIOP144-24|BGE_00108_E01||Guarda|BOLD:AEU1504
Monatractides stadleri|BSNTN1011-23|BGE_00227_F01||Guarda|BOLD:AEU1504
Monatractides stadleri|BSNTN868-23|BGE_00228_B01||Guarda|BOLD:AEU1504
Monatractides stadleri|BSNTN861-23|BGE_00228_A06||Guarda|BOLD:AEU1504
Monatractides madritensis|BBIOP133-24|BGE_00108_D02||Guarda|BOLD:AED3803
Monatractides madritensis|BSNTN1041-23|BGE_00227_H07||Guarda|BOLD:AED3803
Monatractides madritensis|BBIOP030-24|BGE_00109_C06||Braganca|BOLD:AED3803
Monatractides madritensis|BSNTN983-23|BGE_00227_C09||Guarda|BOLD:AED3803

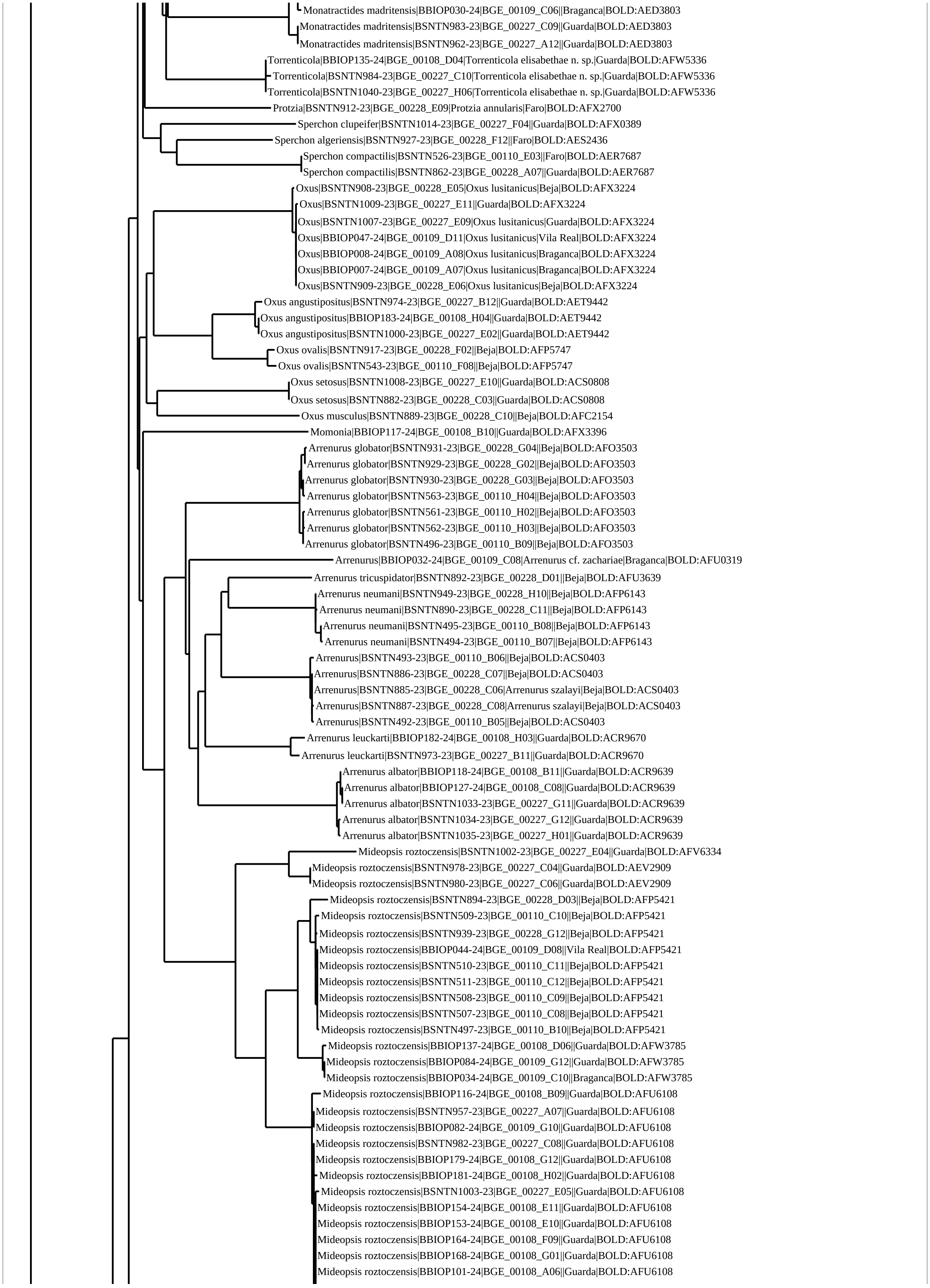
Monatractides madritensis|BBIOP030-24|BGE_00109_C06||Braganca|BOLD:AED3803
Monatractides madritensis|BSNTN983-23|BGE_00227_C09||Guarda|BOLD:AED3803
Monatractides madritensis|BSNTN962-23|BGE_00227_A12||Guarda|BOLD:AED3803
Torrenticola|BBIOP135-24|BGE_00108_D04|Torrenticola elisabethae n. sp.|Guarda|BOLD:AFW5336
Torrenticola|BSNTN984-23|BGE_00227_C10|Torrenticola elisabethae n. sp.|Guarda|BOLD:AFW5336
Torrenticola|BSNTN1040-23|BGE_00227_H06|Torrenticola elisabethae n. sp.|Guarda|BOLD:AFW5336
Protzia|BSNTN912-23|BGE_00228_E09|Protzia annularis|Faro|BOLD:AFX2700
Sperchon clupeifer|BSNTN1014-23|BGE_00227_F04||Guarda|BOLD:AFX0389
Sperchon algeriensis|BSNTN927-23|BGE_00228_F12||Faro|BOLD:AES2436
Sperchon compactilis|BSNTN526-23|BGE_00110_E03||Faro|BOLD:AER7687
Sperchon compactilis|BSNTN862-23|BGE_00228_A07||Guarda|BOLD:AER7687
Oxus|BSNTN908-23|BGE_00228_E05|Oxus lusitanicus|Beja|BOLD:AFX3224
Oxus|BSNTN1009-23|BGE_00227_E11||Guarda|BOLD:AFX3224
Oxus|BSNTN1007-23|BGE_00227_E09|Oxus lusitanicus|Guarda|BOLD:AFX3224
Oxus|BBIOP047-24|BGE_00109_D11|Oxus lusitanicus|Vila Real|BOLD:AFX3224
Oxus|BBIOP008-24|BGE_00109_A08|Oxus lusitanicus|Braganca|BOLD:AFX3224
Oxus|BBIOP007-24|BGE_00109_A07|Oxus lusitanicus|Braganca|BOLD:AFX3224
Oxus|BSNTN909-23|BGE_00228_E06|Oxus lusitanicus|Beja|BOLD:AFX3224
Oxus angustipositus|BSNTN974-23|BGE_00227_B12||Guarda|BOLD:AET9442
Oxus angustipositus|BBIOP183-24|BGE_00108_H04||Guarda|BOLD:AET9442
Oxus angustipositus|BSNTN1000-23|BGE_00227_E02||Guarda|BOLD:AET9442
Oxus ovalis|BSNTN917-23|BGE_00228_F02||Beja|BOLD:AFP5747
Oxus ovalis|BSNTN543-23|BGE_00110_F08||Beja|BOLD:AFP5747
Oxus setosus|BSNTN1008-23|BGE_00227_E10||Guarda|BOLD:ACS0808
Oxus setosus|BSNTN882-23|BGE_00228_C03||Guarda|BOLD:ACS0808
Oxus musculus|BSNTN889-23|BGE_00228_C10||Beja|BOLD:AFC2154
Momonia|BBIOP117-24|BGE_00108_B10||Guarda|BOLD:AFX3396
Arrenurus globator|BSNTN931-23|BGE_00228_G04||Beja|BOLD:AFO3503
Arrenurus globator|BSNTN929-23|BGE_00228_G02||Beja|BOLD:AFO3503
Arrenurus globator|BSNTN930-23|BGE_00228_G03||Beja|BOLD:AFO3503
Arrenurus globator|BSNTN563-23|BGE_00110_H04||Beja|BOLD:AFO3503
Arrenurus globator|BSNTN561-23|BGE_00110_H02||Beja|BOLD:AFO3503
Arrenurus globator|BSNTN562-23|BGE_00110_H03||Beja|BOLD:AFO3503
Arrenurus globator|BSNTN496-23|BGE_00110_B09||Beja|BOLD:AFO3503
Arrenurus|BBIOP032-24|BGE_00109_C08|Arrenurus cf. zachariae|Braganca|BOLD:AFU0319
Arrenurus tricuspidator|BSNTN892-23|BGE_00228_D01||Beja|BOLD:AFU3639
Arrenurus neumani|BSNTN949-23|BGE_00228_H10||Beja|BOLD:AFP6143
Arrenurus neumani|BSNTN890-23|BGE_00228_C11||Beja|BOLD:AFP6143
Arrenurus neumani|BSNTN495-23|BGE_00110_B08||Beja|BOLD:AFP6143
Arrenurus neumani|BSNTN494-23|BGE_00110_B07||Beja|BOLD:AFP6143
Arrenurus|BSNTN493-23|BGE_00110_B06||Beja|BOLD:ACS0403
Arrenurus|BSNTN886-23|BGE_00228_C07||Beja|BOLD:ACS0403
Arrenurus|BSNTN885-23|BGE_00228_C06|Arrenurus szalayi|Beja|BOLD:ACS0403
Arrenurus|BSNTN887-23|BGE_00228_C08|Arrenurus szalayi|Beja|BOLD:ACS0403
Arrenurus|BSNTN492-23|BGE_00110_B05||Beja|BOLD:ACS0403
Arrenurus leuckarti|BBIOP182-24|BGE_00108_H03||Guarda|BOLD:ACR9670
Arrenurus leuckarti|BSNTN973-23|BGE_00227_B11||Guarda|BOLD:ACR9670
Arrenurus albator|BBIOP118-24|BGE_00108_B11||Guarda|BOLD:ACR9639
Arrenurus albator|BBIOP127-24|BGE_00108_C08||Guarda|BOLD:ACR9639
Arrenurus albator|BSNTN1033-23|BGE_00227_G11||Guarda|BOLD:ACR9639
Arrenurus albator|BSNTN1034-23|BGE_00227_G12||Guarda|BOLD:ACR9639
Arrenurus albator|BSNTN1035-23|BGE_00227_H01||Guarda|BOLD:ACR9639
Mideopsis roztoczensis|BSNTN1002-23|BGE_00227_E04||Guarda|BOLD:AFV6334
Mideopsis roztoczensis|BSNTN978-23|BGE_00227_C04||Guarda|BOLD:AEV2909
Mideopsis roztoczensis|BSNTN980-23|BGE_00227_C06||Guarda|BOLD:AEV2909
Mideopsis roztoczensis|BSNTN894-23|BGE_00228_D03||Beja|BOLD:AFP5421
Mideopsis roztoczensis|BSNTN509-23|BGE_00110_C10||Beja|BOLD:AFP5421
Mideopsis roztoczensis|BSNTN939-23|BGE_00228_G12||Beja|BOLD:AFP5421
Mideopsis roztoczensis|BBIOP044-24|BGE_00109_D08||Vila Real|BOLD:AFP5421
Mideopsis roztoczensis|BSNTN510-23|BGE_00110_C11||Beja|BOLD:AFP5421
Mideopsis roztoczensis|BSNTN511-23|BGE_00110_C12||Beja|BOLD:AFP5421
Mideopsis roztoczensis|BSNTN508-23|BGE_00110_C09||Beja|BOLD:AFP5421
Mideopsis roztoczensis|BSNTN507-23|BGE_00110_C08||Beja|BOLD:AFP5421
Mideopsis roztoczensis|BSNTN497-23|BGE_00110_B10||Beja|BOLD:AFP5421
Mideopsis roztoczensis|BBIOP137-24|BGE_00108_D06||Guarda|BOLD:AFW3785
Mideopsis roztoczensis|BBIOP084-24|BGE_00109_G12||Guarda|BOLD:AFW3785
Mideopsis roztoczensis|BBIOP034-24|BGE_00109_C10||Braganca|BOLD:AFW3785
Mideopsis roztoczensis|BBIOP116-24|BGE_00108_B09||Guarda|BOLD:AFU6108
Mideopsis roztoczensis|BSNTN957-23|BGE_00227_A07||Guarda|BOLD:AFU6108
Mideopsis roztoczensis|BBIOP082-24|BGE_00109_G10||Guarda|BOLD:AFU6108
Mideopsis roztoczensis|BSNTN982-23|BGE_00227_C08||Guarda|BOLD:AFU6108
Mideopsis roztoczensis|BBIOP179-24|BGE_00108_G12||Guarda|BOLD:AFU6108
Mideopsis roztoczensis|BBIOP181-24|BGE_00108_H02||Guarda|BOLD:AFU6108
Mideopsis roztoczensis|BSNTN1003-23|BGE_00227_E05||Guarda|BOLD:AFU6108
Mideopsis roztoczensis|BBIOP154-24|BGE_00108_E11||Guarda|BOLD:AFU6108
Mideopsis roztoczensis|BBIOP153-24|BGE_00108_E10||Guarda|BOLD:AFU6108
Mideopsis roztoczensis|BBIOP164-24|BGE_00108_F09||Guarda|BOLD:AFU6108
Mideopsis roztoczensis|BBIOP168-24|BGE_00108_G01||Guarda|BOLD:AFU6108
Mideopsis roztoczensis|BBIOP101-24|BGE_00108_A06||Guarda|BOLD:AFU6108

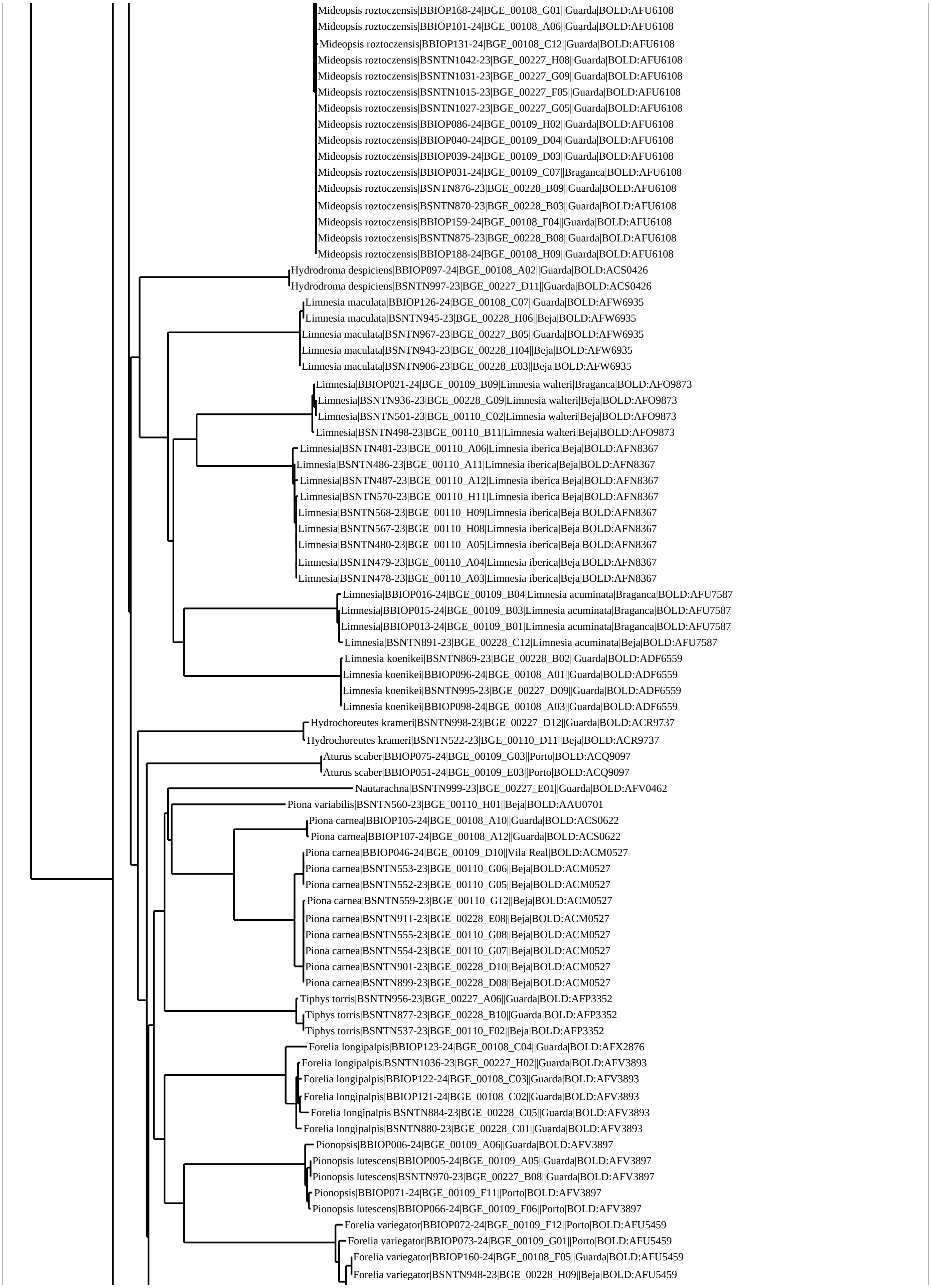
Mideopsis roztoczensis|BBIOP168-24|BGE_00108_G01||Guarda|BOLD:AFU6108
Mideopsis roztoczensis|BBIOP101-24|BGE_00108_A06||Guarda|BOLD:AFU6108
Mideopsis roztoczensis|BBIOP131-24|BGE_00108_C12||Guarda|BOLD:AFU6108
Mideopsis roztoczensis|BSNTN1042-23|BGE_00227_H08||Guarda|BOLD:AFU6108
Mideopsis roztoczensis|BSNTN1031-23|BGE_00227_G09||Guarda|BOLD:AFU6108
Mideopsis roztoczensis|BSNTN1015-23|BGE_00227_F05||Guarda|BOLD:AFU6108
Mideopsis roztoczensis|BSNTN1027-23|BGE_00227_G05||Guarda|BOLD:AFU6108
Mideopsis roztoczensis|BBIOP086-24|BGE_00109_H02||Guarda|BOLD:AFU6108
Mideopsis roztoczensis|BBIOP040-24|BGE_00109_D04||Guarda|BOLD:AFU6108
Mideopsis roztoczensis|BBIOP039-24|BGE_00109_D03||Guarda|BOLD:AFU6108
Mideopsis roztoczensis|BBIOP031-24|BGE_00109_C07||Braganca|BOLD:AFU6108
Mideopsis roztoczensis|BSNTN876-23|BGE_00228_B09||Guarda|BOLD:AFU6108
Mideopsis roztoczensis|BSNTN870-23|BGE_00228_B03||Guarda|BOLD:AFU6108
Mideopsis roztoczensis|BBIOP159-24|BGE_00108_F04||Guarda|BOLD:AFU6108
Mideopsis roztoczensis|BSNTN875-23|BGE_00228_B08||Guarda|BOLD:AFU6108
Mideopsis roztoczensis|BBIOP188-24|BGE_00108_H09||Guarda|BOLD:AFU6108
Hydrodroma despiciens|BBIOP097-24|BGE_00108_A02||Guarda|BOLD:ACS0426
Hydrodroma despiciens|BSNTN997-23|BGE_00227_D11||Guarda|BOLD:ACS0426
Limnesia maculata|BBIOP126-24|BGE_00108_C07||Guarda|BOLD:AFW6935
Limnesia maculata|BSNTN945-23|BGE_00228_H06||Beja|BOLD:AFW6935
Limnesia maculata|BSNTN967-23|BGE_00227_B05||Guarda|BOLD:AFW6935
Limnesia maculata|BSNTN943-23|BGE_00228_H04||Beja|BOLD:AFW6935
Limnesia maculata|BSNTN906-23|BGE_00228_E03||Beja|BOLD:AFW6935
Limnesia|BBIOP021-24|BGE_00109_B09|Limnesia walteri|Braganca|BOLD:AFO9873
Limnesia|BSNTN936-23|BGE_00228_G09|Limnesia walteri|Beja|BOLD:AFO9873
Limnesia|BSNTN501-23|BGE_00110_C02|Limnesia walteri|Beja|BOLD:AFO9873
Limnesia|BSNTN498-23|BGE_00110_B11|Limnesia walteri|Beja|BOLD:AFO9873
Limnesia|BSNTN481-23|BGE_00110_A06|Limnesia iberica|Beja|BOLD:AFN8367
Limnesia|BSNTN486-23|BGE_00110_A11|Limnesia iberica|Beja|BOLD:AFN8367
Limnesia|BSNTN487-23|BGE_00110_A12|Limnesia iberica|Beja|BOLD:AFN8367
Limnesia|BSNTN570-23|BGE_00110_H11|Limnesia iberica|Beja|BOLD:AFN8367
Limnesia|BSNTN568-23|BGE_00110_H09|Limnesia iberica|Beja|BOLD:AFN8367
Limnesia|BSNTN567-23|BGE_00110_H08|Limnesia iberica|Beja|BOLD:AFN8367
Limnesia|BSNTN480-23|BGE_00110_A05|Limnesia iberica|Beja|BOLD:AFN8367
Limnesia|BSNTN479-23|BGE_00110_A04|Limnesia iberica|Beja|BOLD:AFN8367
Limnesia|BSNTN478-23|BGE_00110_A03|Limnesia iberica|Beja|BOLD:AFN8367
Limnesia|BBIOP016-24|BGE_00109_B04|Limnesia acuminata|Braganca|BOLD:AFU7587
Limnesia|BBIOP015-24|BGE_00109_B03|Limnesia acuminata|Braganca|BOLD:AFU7587
Limnesia|BBIOP013-24|BGE_00109_B01|Limnesia acuminata|Braganca|BOLD:AFU7587
Limnesia|BSNTN891-23|BGE_00228_C12|Limnesia acuminata|Beja|BOLD:AFU7587
Limnesia koenikei|BSNTN869-23|BGE_00228_B02||Guarda|BOLD:ADF6559
Limnesia koenikei|BBIOP096-24|BGE_00108_A01||Guarda|BOLD:ADF6559
Limnesia koenikei|BSNTN995-23|BGE_00227_D09||Guarda|BOLD:ADF6559
Limnesia koenikei|BBIOP098-24|BGE_00108_A03||Guarda|BOLD:ADF6559
Hydrochoreutes krameri|BSNTN998-23|BGE_00227_D12||Guarda|BOLD:ACR9737
Hydrochoreutes krameri|BSNTN522-23|BGE_00110_D11||Beja|BOLD:ACR9737
Aturus scaber|BBIOP075-24|BGE_00109_G03||Porto|BOLD:ACQ9097
Aturus scaber|BBIOP051-24|BGE_00109_E03||Porto|BOLD:ACQ9097
Nautarachna|BSNTN999-23|BGE_00227_E01||Guarda|BOLD:AFV0462
Piona variabilis|BSNTN560-23|BGE_00110_H01||Beja|BOLD:AAU0701
Piona carnea|BBIOP105-24|BGE_00108_A10||Guarda|BOLD:ACS0622
Piona carnea|BBIOP107-24|BGE_00108_A12||Guarda|BOLD:ACS0622
Piona carnea|BBIOP046-24|BGE_00109_D10||Vila Real|BOLD:ACM0527
Piona carnea|BSNTN553-23|BGE_00110_G06||Beja|BOLD:ACM0527
Piona carnea|BSNTN552-23|BGE_00110_G05||Beja|BOLD:ACM0527
Piona carnea|BSNTN559-23|BGE_00110_G12||Beja|BOLD:ACM0527
Piona carnea|BSNTN911-23|BGE_00228_E08||Beja|BOLD:ACM0527
Piona carnea|BSNTN555-23|BGE_00110_G08||Beja|BOLD:ACM0527
Piona carnea|BSNTN554-23|BGE_00110_G07||Beja|BOLD:ACM0527
Piona carnea|BSNTN901-23|BGE_00228_D10||Beja|BOLD:ACM0527
Piona carnea|BSNTN899-23|BGE_00228_D08||Beja|BOLD:ACM0527
Tiphys torris|BSNTN956-23|BGE_00227_A06||Guarda|BOLD:AFP3352
Tiphys torris|BSNTN877-23|BGE_00228_B10||Guarda|BOLD:AFP3352
Tiphys torris|BSNTN537-23|BGE_00110_F02||Beja|BOLD:AFP3352
Forelia longipalpis|BBIOP123-24|BGE_00108_C04||Guarda|BOLD:AFX2876
Forelia longipalpis|BSNTN1036-23|BGE_00227_H02||Guarda|BOLD:AFV3893
Forelia longipalpis|BBIOP122-24|BGE_00108_C03||Guarda|BOLD:AFV3893
Forelia longipalpis|BBIOP121-24|BGE_00108_C02||Guarda|BOLD:AFV3893
Forelia longipalpis|BSNTN884-23|BGE_00228_C05||Guarda|BOLD:AFV3893
Forelia longipalpis|BSNTN880-23|BGE_00228_C01||Guarda|BOLD:AFV3893
Pionopsis|BBIOP006-24|BGE_00109_A06||Guarda|BOLD:AFV3897
Pionopsis lutescens|BBIOP005-24|BGE_00109_A05||Guarda|BOLD:AFV3897
Pionopsis lutescens|BSNTN970-23|BGE_00227_B08||Guarda|BOLD:AFV3897
Pionopsis|BBIOP071-24|BGE_00109_F11||Porto|BOLD:AFV3897
Pionopsis lutescens|BBIOP066-24|BGE_00109_F06||Porto|BOLD:AFV3897
Forelia variegator|BBIOP072-24|BGE_00109_F12||Porto|BOLD:AFU5459
Forelia variegator|BBIOP073-24|BGE_00109_G01||Porto|BOLD:AFU5459
Forelia variegator|BBIOP160-24|BGE_00108_F05||Guarda|BOLD:AFU5459
Forelia variegator|BSNTN948-23|BGE_00228_H09||Beja|BOLD:AFU5459

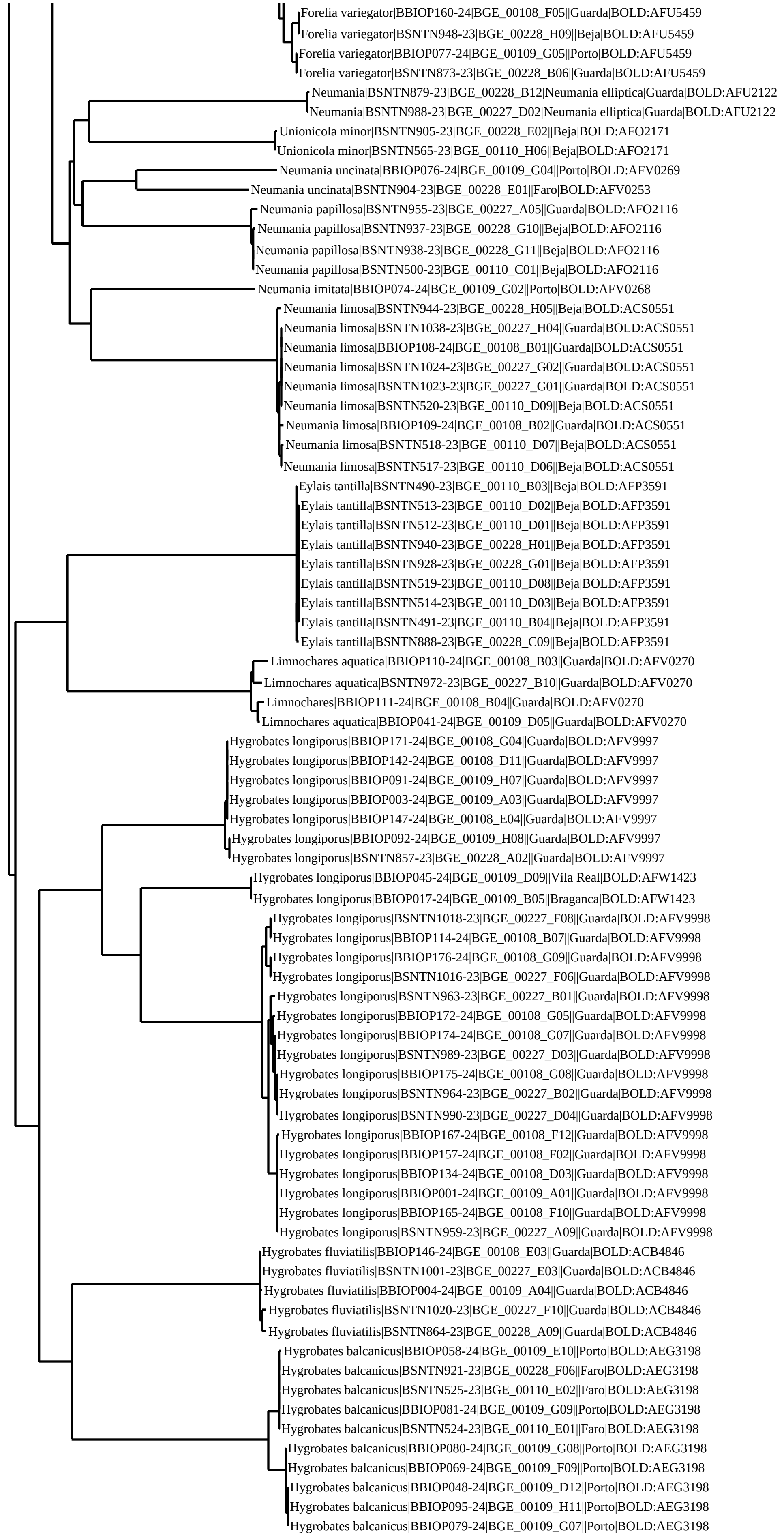
Forelia variegator|BBIOP160-24|BGE_00108_F05||Guarda|BOLD:AFU5459
Forelia variegator|BSNTN948-23|BGE_00228_H09||Beja|BOLD:AFU5459
Forelia variegator|BBIOP077-24|BGE_00109_G05||Porto|BOLD:AFU5459
Forelia variegator|BSNTN873-23|BGE_00228_B06||Guarda|BOLD:AFU5459
Neumania|BSNTN879-23|BGE_00228_B12|Neumania elliptica|Guarda|BOLD:AFU2122
Neumania|BSNTN988-23|BGE_00227_D02|Neumania elliptica|Guarda|BOLD:AFU2122
Unionicola minor|BSNTN905-23|BGE_00228_E02||Beja|BOLD:AFO2171
Unionicola minor|BSNTN565-23|BGE_00110_H06||Beja|BOLD:AFO2171
Neumania uncinata|BBIOP076-24|BGE_00109_G04||Porto|BOLD:AFV0269
Neumania uncinata|BSNTN904-23|BGE_00228_E01||Faro|BOLD:AFV0253
Neumania papillosa|BSNTN955-23|BGE_00227_A05||Guarda|BOLD:AFO2116
Neumania papillosa|BSNTN937-23|BGE_00228_G10||Beja|BOLD:AFO2116
Neumania papillosa|BSNTN938-23|BGE_00228_G11||Beja|BOLD:AFO2116
Neumania papillosa|BSNTN500-23|BGE_00110_C01||Beja|BOLD:AFO2116
Neumania imitata|BBIOP074-24|BGE_00109_G02||Porto|BOLD:AFV0268
Neumania limosa|BSNTN944-23|BGE_00228_H05||Beja|BOLD:ACS0551
Neumania limosa|BSNTN1038-23|BGE_00227_H04||Guarda|BOLD:ACS0551
Neumania limosa|BBIOP108-24|BGE_00108_B01||Guarda|BOLD:ACS0551
Neumania limosa|BSNTN1024-23|BGE_00227_G02||Guarda|BOLD:ACS0551
Neumania limosa|BSNTN1023-23|BGE_00227_G01||Guarda|BOLD:ACS0551
Neumania limosa|BSNTN520-23|BGE_00110_D09||Beja|BOLD:ACS0551
Neumania limosa|BBIOP109-24|BGE_00108_B02||Guarda|BOLD:ACS0551
Neumania limosa|BSNTN518-23|BGE_00110_D07||Beja|BOLD:ACS0551
Neumania limosa|BSNTN517-23|BGE_00110_D06||Beja|BOLD:ACS0551
Eylais tantilla|BSNTN490-23|BGE_00110_B03||Beja|BOLD:AFP3591
Eylais tantilla|BSNTN513-23|BGE_00110_D02||Beja|BOLD:AFP3591
Eylais tantilla|BSNTN512-23|BGE_00110_D01||Beja|BOLD:AFP3591
Eylais tantilla|BSNTN940-23|BGE_00228_H01||Beja|BOLD:AFP3591
Eylais tantilla|BSNTN928-23|BGE_00228_G01||Beja|BOLD:AFP3591
Eylais tantilla|BSNTN519-23|BGE_00110_D08||Beja|BOLD:AFP3591
Eylais tantilla|BSNTN514-23|BGE_00110_D03||Beja|BOLD:AFP3591
Eylais tantilla|BSNTN491-23|BGE_00110_B04||Beja|BOLD:AFP3591
Eylais tantilla|BSNTN888-23|BGE_00228_C09||Beja|BOLD:AFP3591
Limnochares aquatica|BBIOP110-24|BGE_00108_B03||Guarda|BOLD:AFV0270
Limnochares aquatica|BSNTN972-23|BGE_00227_B10||Guarda|BOLD:AFV0270
Limnochares|BBIOP111-24|BGE_00108_B04||Guarda|BOLD:AFV0270
Limnochares aquatica|BBIOP041-24|BGE_00109_D05||Guarda|BOLD:AFV0270
Hygrobates longiporus|BBIOP171-24|BGE_00108_G04||Guarda|BOLD:AFV9997
Hygrobates longiporus|BBIOP142-24|BGE_00108_D11||Guarda|BOLD:AFV9997
Hygrobates longiporus|BBIOP091-24|BGE_00109_H07||Guarda|BOLD:AFV9997
Hygrobates longiporus|BBIOP003-24|BGE_00109_A03||Guarda|BOLD:AFV9997
Hygrobates longiporus|BBIOP147-24|BGE_00108_E04||Guarda|BOLD:AFV9997
Hygrobates longiporus|BBIOP092-24|BGE_00109_H08||Guarda|BOLD:AFV9997
Hygrobates longiporus|BSNTN857-23|BGE_00228_A02||Guarda|BOLD:AFV9997
Hygrobates longiporus|BBIOP045-24|BGE_00109_D09||Vila Real|BOLD:AFW1423
Hygrobates longiporus|BBIOP017-24|BGE_00109_B05||Braganca|BOLD:AFW1423
Hygrobates longiporus|BSNTN1018-23|BGE_00227_F08||Guarda|BOLD:AFV9998
Hygrobates longiporus|BBIOP114-24|BGE_00108_B07||Guarda|BOLD:AFV9998
Hygrobates longiporus|BBIOP176-24|BGE_00108_G09||Guarda|BOLD:AFV9998
Hygrobates longiporus|BSNTN1016-23|BGE_00227_F06||Guarda|BOLD:AFV9998
Hygrobates longiporus|BSNTN963-23|BGE_00227_B01||Guarda|BOLD:AFV9998
Hygrobates longiporus|BBIOP172-24|BGE_00108_G05||Guarda|BOLD:AFV9998
Hygrobates longiporus|BBIOP174-24|BGE_00108_G07||Guarda|BOLD:AFV9998
Hygrobates longiporus|BSNTN989-23|BGE_00227_D03||Guarda|BOLD:AFV9998
Hygrobates longiporus|BBIOP175-24|BGE_00108_G08||Guarda|BOLD:AFV9998
Hygrobates longiporus|BSNTN964-23|BGE_00227_B02||Guarda|BOLD:AFV9998
Hygrobates longiporus|BSNTN990-23|BGE_00227_D04||Guarda|BOLD:AFV9998
Hygrobates longiporus|BBIOP167-24|BGE_00108_F12||Guarda|BOLD:AFV9998
Hygrobates longiporus|BBIOP157-24|BGE_00108_F02||Guarda|BOLD:AFV9998
Hygrobates longiporus|BBIOP134-24|BGE_00108_D03||Guarda|BOLD:AFV9998
Hygrobates longiporus|BBIOP001-24|BGE_00109_A01||Guarda|BOLD:AFV9998
Hygrobates longiporus|BBIOP165-24|BGE_00108_F10||Guarda|BOLD:AFV9998
Hygrobates longiporus|BSNTN959-23|BGE_00227_A09||Guarda|BOLD:AFV9998
Hygrobates fluviatilis|BBIOP146-24|BGE_00108_E03||Guarda|BOLD:ACB4846
Hygrobates fluviatilis|BSNTN1001-23|BGE_00227_E03||Guarda|BOLD:ACB4846
Hygrobates fluviatilis|BBIOP004-24|BGE_00109_A04||Guarda|BOLD:ACB4846
Hygrobates fluviatilis|BSNTN1020-23|BGE_00227_F10||Guarda|BOLD:ACB4846
Hygrobates fluviatilis|BSNTN864-23|BGE_00228_A09||Guarda|BOLD:ACB4846
Hygrobates balcanicus|BBIOP058-24|BGE_00109_E10||Porto|BOLD:AEG3198
Hygrobates balcanicus|BSNTN921-23|BGE_00228_F06||Faro|BOLD:AEG3198
Hygrobates balcanicus|BSNTN525-23|BGE_00110_E02||Faro|BOLD:AEG3198
Hygrobates balcanicus|BBIOP081-24|BGE_00109_G09||Porto|BOLD:AEG3198
Hygrobates balcanicus|BSNTN524-23|BGE_00110_E01||Faro|BOLD:AEG3198
Hygrobates balcanicus|BBIOP080-24|BGE_00109_G08||Porto|BOLD:AEG3198
Hygrobates balcanicus|BBIOP069-24|BGE_00109_F09||Porto|BOLD:AEG3198
Hygrobates balcanicus|BBIOP048-24|BGE_00109_D12||Porto|BOLD:AEG3198
Hygrobates balcanicus|BBIOP095-24|BGE_00109_H11||Porto|BOLD:AEG3198
Hygrobates balcanicus|BBIOP079-24|BGE_00109_G07||Porto|BOLD:AEG3198
