# Supplementary material for: ﻿DNA barcode library of Portuguese water mites, with the descriptions of two new species (Acari, Hydrachnidia)
Source: Zookeys. 2024 Oct 31;1217:119–71. doi: 10.3897/zookeys.1217.131730 (PMC11544308; doi:10.3897/zookeys.1217.131730)
Supplement: Supplementary material 2 — List of Torrenticola specimens [file zookeys-1217-119_article-131730__-s002.doc]

**Supplementary material 2.** List of *Torrenticola* specimens used for building the Neighbour-Joining (NJ) tree (Fig. 6). Details on the specimens from Portugal are given in Table 1. GenBank numbers are indicated by *. BINs are based on the barcode analysis from 8 May 2024. Country codes (alpha-2 code): AT = Austria, BA = Bosnia and Herzegovina, CR = Croatia, DE = Germany, ES = Spain, FR = France, GR = Greece, IR = Iran, IT = Italy, NO = Norway, NL = Netherlands, ME = Montenegro, MK = North Macedonia, PT = Portugal, RS = Serbia, TR = Turkey.

| **Taxon** | **BOLD IDs** | **Voucher Code** | **Country** | **BIN BOLD** |
| --- | --- | --- | --- | --- |
| *T. amplexa* | NLACA081-15 | RMNH.ACA.1113 | NL | ACS0261 |
|  | NLACA454-15 | RMNH.ACA.883 | NL |  |
|  | NLACA080-15 | RMNH.ACA.1112 | NL |  |
|  | NLACA082-15 | RMNH.ACA.1114 | NL |  |
|  | NLACA453-15 | RMNH.ACA.881 | NL |  |
|  | SEPTB081-21 | CCDB 38362 G09 | CR |  |
|  | HYDOC081-22 | CCDB 44300 G09 | CR | ACR0665 |
|  | HYDOC083-22 | CCDB 44300 G11 | CR |  |
|  | HYDOC079-22 | CCDB 44300 G07 | CR |  |
|  | DNCBD068-20 | CCDB-3867-F08 | ME |  |
|  | DNCBD069-20 | CCDB-3867-F09 | ME |  |
|  | DCCDB076-21 | CCDB38233 G04 | ME |  |
|  | DCBDJ064-21 | CCDB 38392 F04 | DE |  |
|  | MARBN611-23 | MARB UIB 694 | NO |  |
|  | HYDCA109-18 | HYDCA109 | NO |  |
|  | HYDAL028-23 | CCDB-44301-C04 | NO |  |
|  | MARBN630-23 | MARB UIB 713 | NO |  |
|  | MARBN646-23 | MARB UIB 729 | NO |  |
|  | MARBN615-23 | MARB UIB 698 | NO |  |
|  | MARBN616-23 | MARB UIB 699 | NO |  |
|  | MARBN626-23 | MARB UIB 709 | NO |  |
|  | MARBN627-23 | MARB UIB 710 | NO |  |
|  | MARBN640-23 | MARB UIB 723 | NO |  |
|  | MARBN641-23 | MARB UIB 724 | NO |  |
|  | MARBN648-23 | MARB UIB 731 | NO |  |
|  | MARBN631-23 | MARB UIB 714 | NO |  |
|  | MARBN1144-23 | MARB UIB 752 | NO |  |
|  | MARBN647-23 | MARB UIB 730 | NO |  |
|  | MARBN1242-23 | MARB UIB 850 | NO |  |
| *T. anomala* | SEPTA059-21 | CCDB 38363 E11 | RS | ACI0434 |
| *T. barsica* | DNCBD064-20 | CCDB-3867-F04 | ME | AEF1219 |
|  | SEPTA054-21 | CCDB 38363 E06 | RS |  |
|  | DNCBD057-20 | CCDB-3867-E09 | ME |  |
|  | HYDOC006-22 | CCDB 44300 A06 | RS |  |
| *T. baueri* | HYDIR040-23 | CCDB 39399 D04 | IR | AFG4655 |
|  | HYDIR023-23 | CCDB 39399 B11 | TR |  |
|  | HYDIR024-23 | CCDB 39399 B12 | TR |  |
|  | HYDIR049-23 | CCDB 39399 E01 | IR |  |
|  | HYDIR042-23 | CCDB 39399 D06 | IR |  |
|  | HYDIR039-23 | CCDB 39399 D03 | IR |  |
|  | HYDIR043-23 | CCDB 39399 D07 | IR |  |
| *T. brevirostris* | HYDOC020-22 | CCDB 44300 B08 | RS | AED9586 |
|  | HYDOC021-22 | CCDB 44300 B09 | RS |  |
|  | DNAEC073-20 | 42. M19 29A 5 G6 | ME |  |
|  | SEPTA036-21 | CCDB 38363 C12 | ME |  |
|  | MARBN1277-23 | MARB UIB 885 | NO |  |
|  | MARBN1276-23 | MARB UIB 884 | NO |  |
| *T. dowlingi* | IRANM003-20 | 7. IR1 2017 H1 | IR | AED4882 |
| *T. dudichi* | DNAEC074-20 | 43. M19 16A 4 G7 | ME | AED7520 |
|  | DCCDB047-21 | CCDB38233 D11 | ME |  |
| *T. elliptica* Macedonia | *OHHKK150-23 | PP545639 | MK |  |
| *T. elliptica* Montenegro | DCCDB023-21 | CCDB38233 B11 | ME | AEI9183 |
| *T. eseni* | HYDIR011-23 | CCDB 39399 A11 | TR | AFG1657 |
|  | HYDIR012-23 | CCDB 39399 A12 | TR |  |
|  | HYDIR018-23 | CCDB 39399 B06 | TR |  |
|  | HYDIR022-23 | CCDB 39399 B10 | TR |  |
| *T. hispanica* | HYDAS022-22 | CCDB 39397 B10 | PT | AES2742 |
| *T. meridionalis* | *OHHKK148-23 | PP545739 | MK | AED7519 |
|  | *OHHKK149-23 | PP545797 | MK |  |
|  | *OHHKK144-23 | PP545573 | MK |  |
|  | *OHHKK151-23 | PP545782 | MK |  |
|  | *OHHKK147-23 | PP545710 | MK |  |
|  | *OHHKK146-23 | PP545663 | MK |  |
|  | DNAEC023-20 | 18. CG2019 7 C1 | MK |  |
|  | DNCBD049-20 | CCDB-3867-E01 | ME |  |
|  | DNCBD051-20 | CCDB-3867-E03 | ME |  |
|  | DNAEC022-20 | 15. CG2019 7 B12 | MK |  |
|  | HYDBH026-22 | CCDB 41824 C02 | BA |  |
|  | DCDDJ038-21 | CCDB 41824 B04 | ME |  |
|  | HYDBH016-22 | CCDB-3867-B09 | BA |  |
|  | DNCBD021-20 | CCDB-3867-B09 | ME |  |
|  | DNCBD073-20 | CCDB-3867-G01 | ME |  |
|  | DNCBD074-20 | CCDB-3867-G02 | ME |  |
|  | DCBDJ041-21 | CCDB 38392 D05 | IT | AEK9662 |
|  | DCBDJ067-21 | CCDB 38392 F07 | IT |  |
|  | DCBDJ062-21 | CCDB 38392 F02 | IT |  |
|  | HYDME089-22 | CCDB 41823 H05 | ME | AEI3402 |
|  | DCDDJ020-21 | CCDB 38361 B08 | ME |  |
|  | HYDBH002-22 | CCDB 41824 A02 | ME |  |
|  | DCCDB022-21 | CCDB38233 B10 | ME |  |
|  | HYDAS060-22 | CCDB 39397 E12 | AT |  |
|  | HYDAS056-22 | CCDB 39397 E08 | AT |  |
|  | DCCDB048-21 | CCDB38233 D12 | ME |  |
|  | DCDDJ037-21 | CCDB 38361 D01 | ME |  |
| *T. similis* | DCDDJ021-21 | CCDB 38361 B09 | ME | AEK9661 |
| *T. ichnophallus* | DCBDJ061-21 | CCDB 38392 F01 | DE | ACI0619 |
|  | HYDBH089-22 | CCDB 41824 H05 | DE |  |
| *T.* *jeanneli* | HYDAL041-23 | CCDB-44301-D05 | BA | AFB8977 |
| *T. laskai* | DNAEC037-20 | 43. SR CRNOVRSKA D4 | RS | AED2306 |
|  | HYDOC008-22 | CCDB 44300 A08 | RS |  |
|  | DNAEC036-20 | 42. SR CRNOVRSKA D3 | RS |  |
|  | DNCBD059-20 | CCDB-3867-E11 | ME |  |
|  | SEPTA094-21 | CCDB 38363 H10 | GR | AEF5471 |
|  | DNCBD022-20 | CCDB-3867-B10 | ME |  |
|  | DNCBD078-20 | CCDB-3867-G06 | ME |  |
|  | NOVMB067-21 | CCDB 38559 F07 | FR | AEO1170 |
|  | NOVMB083-21 | CCDB 38559 G11 | FR |  |
|  | HYDBH060-22 | CCDB 41824 E12 | GR |  |
|  | HYDAL095-23 | CCDB-44301-H11 | IT | AET4248 |
| *T. lukai* | HYDBH031-22 | CCDB 41824 C07 | BA | ACH9685 |
|  | HYDBH036-22 | CCDB 41824 C12 | BA |  |
|  | HYDBH029-22 | CCDB 41824 C05 | BA |  |
|  | HYDBH023-22 | CCDB 41824 B11 | BA |  |
|  | HYDBH022-22 | CCDB 41824 B10 | BA |  |
|  | HYDBH025-22 | CCDB 41824 C01 | BA |  |
|  | HYDBH030-22 | CCDB 41824 C06 | BA |  |
|  | DCDDJ036-21 | CCDB 38361 C12 | ME |  |
| *T. lundbladi* | *JX629052.1 | AV0001-13 | ES |  |
|  | *JX629051.1 | AV0001-12 | ES |  |
|  | *JX629050.1 | AV0001-3 | ES |  |
| *T. similis* | DCDDJ021-21 | CCDB 38361 B09 | ME | AEK9661 |
| *T. ungeri* | SEPTA051-21 | CCDB 38363 E03 | RS | AED2307 |
|  | DNCBD044-20 | CCDB-3867-D08 | ME |  |
|  | DNCBD079-20 | CCDB-3867-G07 | ME |  |
|  | DNAEC057-20 | 19. M19 24 6 E10 | ME |  |
|  | DNAEC058-20 | 20. M19 24 6 E11 | ME |  |
| *T. xylurgella* | HYDBH056-22 | CCDB 41824 E08 | IT | AET4249 |
|  | HYDBH058-22 | CCDB 41824 E10 | IT |  |
|  | HYDBH059-22 | CCDB 41824 E11 | IT |  |
